# A Genome-Wide Association Study of Pulmonary Function Measures in the Framingham Heart Study

**DOI:** 10.1371/journal.pgen.1000429

**Published:** 2009-03-20

**Authors:** Jemma B. Wilk, Ting-hsu Chen, Daniel J. Gottlieb, Robert E. Walter, Michael W. Nagle, Brian J. Brandler, Richard H. Myers, Ingrid B. Borecki, Edwin K. Silverman, Scott T. Weiss, George T. O'Connor

**Affiliations:** 1 Department of Neurology, Boston University School of Medicine, Boston, Massachusetts, United States of America; 2 Pulmonary Center, Department of Medicine, Boston University School of Medicine, Boston, Massachusetts, United States of America; 3 The National Heart, Lung, and Blood Institute's Framingham Heart Study, Framingham, Massachusetts, United States of America; 4 Veteran's Affairs Boston Healthcare System, Boston, Massachusetts, United States of America; 5 Division of Statistical Genomics, Washington University School of Medicine, St. Louis, Missouri, United States of America; 6 Channing Laboratory, Brigham and Women's Hospital, Harvard Medical School, Boston, Massachusetts, United States of America; University of Oxford, United Kingdom

## Abstract

The ratio of forced expiratory volume in one second to forced vital capacity (FEV_1_/FVC) is a measure used to diagnose airflow obstruction and is highly heritable. We performed a genome-wide association study in 7,691 Framingham Heart Study participants to identify single-nucleotide polymorphisms (SNPs) associated with the FEV_1_/FVC ratio, analyzed as a percent of the predicted value. Identified SNPs were examined in an independent set of 835 Family Heart Study participants enriched for airflow obstruction. Four SNPs in tight linkage disequilibrium on chromosome 4q31 were associated with the percent predicted FEV_1_/FVC ratio with *p*-values of genome-wide significance in the Framingham sample (best *p*-value = 3.6e-09). One of the four chromosome 4q31 SNPs (rs13147758; *p*-value 2.3e-08 in Framingham) was genotyped in the Family Heart Study and produced evidence of association with the same phenotype, percent predicted FEV_1_/FVC (*p*-value = 2.0e-04). The effect estimates for association in the Framingham and Family Heart studies were in the same direction, with the minor allele (G) associated with higher FEV_1_/FVC ratio levels. Results from the Family Heart Study demonstrated that the association extended to FEV_1_ and dichotomous airflow obstruction phenotypes, particularly among smokers. The SNP rs13147758 was associated with the percent predicted FEV_1_/FVC ratio in independent samples from the Framingham and Family Heart Studies producing a combined *p*-value of 8.3e-11, and this region of chromosome 4 around 145.68 megabases was associated with COPD in three additional populations reported in the accompanying manuscript. The associated SNPs do not lie within a gene transcript but are near the hedgehog-interacting protein (*HHIP*) gene and several expressed sequence tags cloned from fetal lung. Though it is unclear what gene or regulatory effect explains the association, the region warrants further investigation.

## Author Summary

Cigarette smoking is the primary risk factor for impaired lung function, yet only 20% of smokers develop chronic obstructive pulmonary disease (COPD). This observation, along with family studies of lung function and COPD, suggests that genetic factors influence susceptibility to cigarette smoke. We examined the relationship between common genetic variants and measures of lung function in a sample of 7,691 participants from the Framingham Heart Study and confirmed our observations in 835 participants from the Family Heart Study selected to include cases of airflow obstruction. We identified a variant on chromosome 4 that was strongly associated with FEV_1_/FVC in the Framingham Study and confirmed the association in the Family Heart Study. The accompanying manuscript identified the same region to be associated with COPD. Several interesting genes are present in the region that we identified, including a gene (*HHIP*) interacting with a biological pathway involved in lung development, but it is not yet clear which gene in the region explains the association. Our results identified a region of chromosome 4 that warrants further study to understand the genetic effects influencing lung function.

## Introduction

Chronic obstructive pulmonary disease (COPD) is the fourth leading cause of death in the US and one of the most prevalent disabling diseases of adults. One of the defining features of COPD is airflow obstruction that is not fully reversible when contrasting measured spirometry before and after administration of a bronchodilator medication. A diagnosis of airflow obstruction is made utilizing the ratio of the spirometric measures forced expiratory volume in the first second (FEV_1_) to forced vital capacity (FVC). The FEV_1_/FVC ratio is reduced in obstructive lung diseases, most notably COPD and asthma. FEV_1_, a measure of airflow, is commonly used to predict clinical outcomes, grade severity and follow the natural history of the disease in staging systems such as the one employed by The Global Initiative for Chronic Obstructive Lung Disease [1]. Low FEV_1_ can be due to restrictive or obstructive lung disease, whereas low FEV_1_/FVC is a more specific indicator of airflow obstruction.

Tobacco smoking is the major environmental cause of COPD, but genetic factors also influence the risk of developing this condition. COPD aggregates in families [2], and spirometric measurements of pulmonary function are heritable [3],[4]. A severe deficiency of alpha-1-antitrypsin, caused by mutations of the *SERPINA1* (*AAT*) gene, causes premature and severe emphysema (OMIM 107400); however, *SERPINA1* mutations explain only a small proportion of cases of COPD. The association of *SERPINE2* SNPs with risk of COPD has been successfully replicated in independent samples [5], but the functional role of *SERPINE2* in the development of COPD remains to be characterized [6]. A number of other candidate gene associations reported in the literature have failed to replicate convincingly [7]. At present, the genetic factors other than *SERPINA1* mutations that increase susceptibility to COPD remain uncertain.

One approach to identifying genes as potential risk factors for COPD is by studying quantitative measures of lung function in population based samples using genome-wide linkage and association methods. Genome-wide linkage to quantitative spirometry traits such as FEV_1_, FVC, and the FEV_1_/FVC ratio [8] has led to the identification of positional candidate genes like *SMOC2* [9],[10]. Quantitative spirometry traits were examined in a previous genome-wide association study using 70,987 single nucleotide polymorphisms (SNPs) in about 1220 related individuals in the Framingham Heart Study [11]. Though no results met the strict criteria for genome-wide significance, *GSTO2* emerged as a candidate gene that warranted replication studies.

The Framingham Heart Study (FHS) has collected spirometry and smoking history data on three generations of adults, and these research participants provided DNA samples that have recently been genotyped for 550,000 SNPs using microarray technology. These genotype and phenotype data, which have been made publicly available through the NHLBI's SNP Health Association Resource (SHARe) initiative (http://public.nhlbi.nih.gov/GeneticsGenomics/home/share.aspx), provide a powerful resource to conduct genome-wide association studies with the goal of discovering novel genetic risk factors for airflow obstruction. Since the prevalence of moderate to severe COPD in this population based sample is low and genetic studies of quantitative traits are thought to have higher statistical power than dichotomous traits, we report here the findings from a genome-wide association (GWA) study for the quantitative pulmonary function measure FEV_1_/FVC, analyzed as a percent of predicted.

The Family Heart Study was developed from existing epidemiologic studies in Forsyth County, NC, Framingham, MA, Minneapolis, MN, and Salt Lake City, UT. Participants were invited to provide a family health history and of those responding, a subset of families was enrolled for a clinical examination that included cross-sectional spirometry and smoking history data as well as DNA for genetic studies [12]. Genome-wide linkage analyses were previously reported for FEV_1_, FVC, and FEV_1_/FVC [13], and many of the DNA samples were available for further study. We now report the results of SNP association with the percent predicted FEV_1_/FVC ratio in the Family Heart Study sample that was undertaken to evaluate independent replication of findings arising in the Framingham GWA.

## Methods

### Ethics Statement

All participants provided written informed consent and local institutional review boards approved the study protocols.

### Framingham and Family Heart Samples

The Framingham Heart Study has measured spirometry on three generations of families from clinical examinations that began in 1948. Original Cohort participants, their Offspring and offspring spouses, and the Third Generation (GEN3) of participants were recruited and examined in Framingham, Massachusetts [14]. A total of 7691 white participants had both genotyping and spirometry available.

A sample of 835 non-asthmatic white Family Heart Study participants, including 225 cases of spirometrically defined airflow obstruction and 610 controls, was used to screen Framingham GWA analysis results. The sample excluded Framingham field center participants in order to define an independent replication sample that included 91 families (545 individuals) with 81 cases of spirometrically defined obstructive pulmonary disease and a case/control sample with an additional 144 cases and 146 controls frequency-matched to cases on age and smoking status. Case and control criteria for airflow obstruction were defined by spirometry as described below. Controls included both family members of cases and unrelated controls.

### Percent Predicted Spirometry Phenotypes

Spirometry from each Framingham participant's latest examination with acceptable pulmonary function data was used; eligible examinations included Cohort exams 19, 17, 16 and 13, Offspring exams 7, 6, 5, and 3, and GEN3 exam 1. Predicted values for FEV_1_ and FEV_1_/FVC were calculated using cohort and gender-specific regression models predicting spirometry measurements on the basis of age, age squared, and height squared among Framingham subjects who were lifetime nonsmokers and had no history of chronic bronchitis, pulmonary disease, COPD/emphysema, asthma, or wheezing. The percent predicted value was calculated by dividing the observed by the predicted value. Standardized residuals were then created by regressing the percent predicted on smoking status (never, former, current) coded using dummy variables to indicate current smoking (yes/no) and former smoking (yes/no), pack-years, and body mass index (BMI: kg/m^2^), in cohort and gender-specific models. The standardized residuals for percent predicted FEV_1_ and FEV_1_/FVC ratio were correlated at 0.52 (p-value<0.0001) in the Framingham participants. The percent predicted FEV_1_/FVC ratio standardized residual was examined as the primary quantitative trait in the GWA.

Spirometry in the Family Heart Study was performed at only one point in time, thus the data are cross-sectional. The observed spirometry was compared to expected values in order to define a percent predicted measure that was further adjusted for smoking status, pack-years, and BMI in gender-specific models using all participants with spirometry. The subset of participants genotyped was analyzed with a FEV_1_/FVC ratio standardized residual generated from the larger sample, paralleling the analysis of Framingham data.

### Dichotomous Airflow Obstruction Phenotypes

In both Framingham and Family Heart studies, a dichotomous trait for airflow obstruction was defined by a percent predicted FEV_1_/FVC ratio less than 90 and a percent predicted FEV_1_ less than 80. These values were selected to standardize a definition for mild airflow obstruction across cohorts and gender using the percent predicted FEV_1_/FVC ratio, which accounts for the normal reduction in FEV_1_/FVC with increasing age [15]. Controls used in these analyses were required to have neither a percent predicted FEV_1_/FVC ratio less than 90 nor a percent predicted FEV_1_ less than 80, which results in a smaller sample size for studies of the dichotomous trait compared to studies of percent predicted FEV_1_/FVC ratio. In Framingham, asthma was classified based on self-report at Cohort exam 1, 3, or 5, Offspring exam 7, and GEN3 exam 1. In the Family Heart Study, asthma was also classified based on self-report.

### Affymetrix 550K Genotyping

Framingham participants were genotyped using the Affymetrix (Santa Clara, CA) GeneChip Human Mapping 500K Array Set, which was comprised of two arrays generating approximately 262,000 SNPs with Nsp arrays and 238,000 SNPs with Sty arrays. An additional Affymetrix 50K Array (HuGeneFocused50K) with gene-centric and coding SNPs was also genotyped for a total of approximately 550K SNPs.

### Analysis of Genotyped SNPs

For population-based analyses of quantitative traits, we examined standardized residuals using linear-mixed effects (LME) models with fixed effects for SNP genotypes and random effects for individuals correlated within families due to polygenic/familial shared effects [16]. Logistic regression via generalized estimating equations (GEE) to account for correlated observations in pedigrees and adjust for covariates was used for dichotomous outcomes. Family-based association tests were implemented in the FBAT software [17]. To assess population stratification in the Framingham sample, principal components were generated using Eigenstrat [18]. None of the first ten components was significantly (p<0.05) related to the percent predicted FEV_1_/FVC ratio, suggesting that population substructure was unlikely to confound the population-based association analysis. Analyses implemented additive genetic models using individuals with ≥97% genotyping call-rate. SNP results were filtered on Hardy-Weinberg Equilibrium p-value of 1×10^−6^, SNP call-rate of 95%, and Minor Allele Frequency of 0.01.

### Genotype Imputation and Analysis

Imputation implemented in MACH [19] (http://www.sph.umich.edu/csg/abecasis/MACH/) was applied to the GWA data to produce imputed genotypes on 2,540,223 HapMap SNPs. An additive model was used for association analysis of the dosage data for imputed genotypes, which provided information on new SNPs and new genotypes for SNPs with previously missing data. The ratio of the empirically observed dosage variance to the expected (binomial) dosage variance was computed as a quality control metric for imputed SNPs [20], and only those with a ratio above 0.9 were considered of high quality.

### Replication in Family Heart Study Participants

LME model results were used to rank the regions identified in the GWA of percent predicted FEV_1_/FVC in Framingham data. A SNP from each of the ten regions with the lowest LME p-values was genotyped in the set of 835 Family Heart Study participants. As only one region met genome-wide statistical significance, selecting ten regions for follow-up was arbitrary. Genotyping was performed using TaqMan technology implemented on the ABI PRISM 7900HT Sequence Detection system at Boston University School of Medicine. The ten SNPs were analyzed for association with the percent predicted FEV_1_/FVC phenotype in the Family Heart Study sample. Based on testing association to ten SNPs representing the top regions of association, a Bonferroni p-value of 0.005 was considered evidence of association in the replication study. Results from screening these top ten regions led to testing additional SNPs and phenotypes on chromosome 4.

## Results

Descriptive statistics of the Framingham and Family Heart Study samples are provided in Table 1. Though the proportion of current smokers is similar across Framingham cohorts, GEN3 had more never-smoking participants than did the older generations. The proportion of participants reporting asthma was greater in the younger generations, and the proportion of participants meeting criteria for COPD was higher in the older generations. The higher proportion of current smokers and airflow obstruction in the Family Heart Study reflects the selection of the sample.

**Table 1:** Descriptive characteristics of the Framingham and Family Heart Study participants.

	Framingham Cohort		Framingham Offspring		Framingham GEN3		Family Heart Study	
	Male (n = 326)	Female (n = 556)	Male (n = 1508)	Female (n = 1696)	Male (n = 1708)	Female (n = 1897)	Male (n = 417)	Female (n = 418)
Age	71.6±5.3	72.0±5.5	59.8±9.6	59.6±9.7	40.3±8.7	40.1±8.7	55.2±12.8	54.2±12.9
BMI	27.0±3.9	26.4±4.8	28.5±4.4	27.5±5.8	27.9±4.5	26.0±6.0	27.9±4.3	27.0±5.9
Current smoking	12.6%	15.1%	15.1%	16.3%	15.9%	14.8%	23.7%	23.2%
Former smoking	59.8%	36.5%	53.3%	44.8%	24.6%	30.6%	46.3%	25.4%
Pack-years*	36.8±22.7	22.8±20.3	32.1±24.7	23.8±21.4	16.2±16.0	11.8±12.1	37.6±28.7	29.0±22.0
% predicted FEV_1_	92.7±20.2	99.8±20.1	93.2±17.4	94.4±17.7	97.8±12.2	98.5±12.0	91.0±21.1	95.1±20.5
% predicted FEV_1_/FVC	92.7±11.9	95.1±10.6	95.9±10.6	96.0±10.4	98.5±7.9	98.7±7.5	89.9±13.2	90.5±11.0
% airflow obstruction	19.9%	10.3%	12.3%	11.3%	3.8%	3.1%	30.2%	23.2%
% asthma	4.7%	2.9%	10.1%	13.3%	13.3%	16.8%	0%	0%

*: Pack-year mean and standard deviation computed among former and current smokers only.

**Figure 1:**
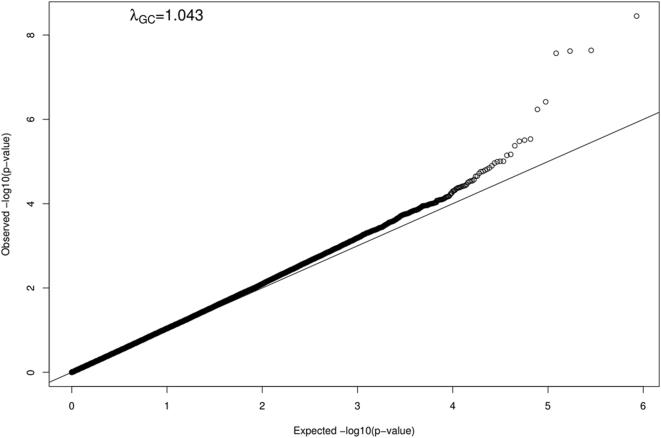
Quantile-Quantile Plot of GWA results for percent predicted FEV_1_/FVC.

The GWA results for percent predicted FEV_1_/FVC based on LME analysis are reported in Table 2 and a Q-Q plot of the results is presented in Figure 1. Ten top regions of interest are described based on 18 SNPs with p-values less than 1.5E-05. The genomic inflation factor value of 1.043 suggested the results were appropriately distributed. Only one region (four SNPs) reached the threshold for genome-wide statistical significance (p<5E-08), which was located on chromosome 4 in an intergenic region near the gene *HHIP*. Also of note among the top regions identified was the *MMP15* gene, which belongs to the matrix metalloproteinase (MMP) family. MMPs have been previously implicated in pulmonary disease, although this particular gene has not been related to COPD development. Table 2 also presents corresponding p-values from analysis of adjusted standardized residuals with FBAT, which is a more conservative test than LME but protects against population stratification. The FBAT results in the *HHIP* region include the lowest FBAT p-values observed genome-wide and support the strong association observed using the LME model.

**Table 2:** Top 10 regions implicated by LME results for percent predicted FEV_1_/FVC in an additive genetic model studying genotyped SNPs in the Framingham Heart Study.

rs#	Chrom	position	MAF	FBAT p-value*	LME beta estimate	LME p-value	Genes within 100 kb
rs11100860	4	145698589	0.388	1.31E-06	0.104	3.55E-09	*HHIP*
rs13147758	4	145679680	0.385	6.92E-06	0.098	2.31E-08	
rs7655625	4	145705365	0.389	6.27E-06	0.098	2.4E-08	*HHIP*
rs1980057	4	145705188	0.387	6.54E-06	0.097	2.7E-08	*HHIP*
rs1828591	4	145700230	0.392	8.63E-06	0.090	3.83E-07	*HHIP*
rs1512285	4	145670409	0.461	0.00013	−0.09	2.93E-06	
rs973796	4	145643418	0.369	0.000157	0.08	7.14E-06	
rs6471895	8	61787503	0.037	0.0161	−0.221	5.8E-07	*CHD7*
rs1017444	2	117491841	0.036	0.00015	−0.212	3.12E-06	
rs17086172	18	68378001	0.057	0.119	−0.170	3.31E-06	*CBLN2*
rs7707619	5	72715410	0.011	0.00833	−0.356	4.22E-06	*FOXD1*
rs994960	3	73814606	0.482	9.82E-05	−0.076	6.79E-06	*PDZRN3*
rs2137064	3	73833683	0.473	4.06E-05	−0.075	9.88E-06	*PDZRN3*
rs1877252	3	73810230	0.482	0.000185	−0.074	1.01E-05	*PDZRN3*
rs17646919	22	28730861	0.085	0.0362	0.134	9.84E-06	*MTMR3*
rs2304488	16	56631711	0.202	0.000672	−0.092	1.09E-05	*MMP15*
rs573461	7	51403173	0.279	0.095	0.083	1.26E-05	*COBL*
rs2456526	15	50876734	0.138	0.0197	−0.107	1.41E-05	*ONECUT1*

*: The direction of effect indicated by the sign of the FBAT Z score was consistent with the direction of the LME beta estimate for all SNPs.

**Figure 2:**
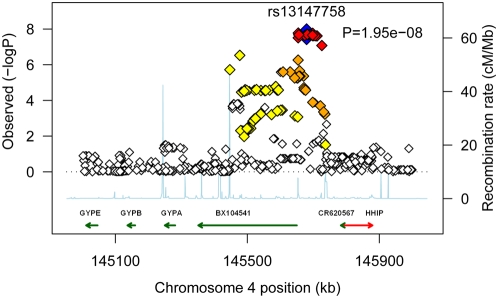
Plot of chromosome 4 association with percent predicted FEV_1_/FVC in imputed Framingham Heart Study data. X-axis is the physical position in kb with arrows denoting genes and expressed sequence tags in the region, the left y-axis plots the −log(p-value), and the right y-axis plots the CEPH recombination rate. The blue diamond identifies the labeled SNP of interest, and LD with the SNP is depicted by color of additional diamonds: red-strong LD (r^2^≥0.8), orange-moderate LD (0.5≤r^2^<0.8), yellow-low LD (0.2≤r^2^<0.5), white-no LD (r^2^<0.2).

The results of the GWA for percent predicted FEV_1_/FVC using imputed SNP genotypes were evaluated to determine whether new regions were implicated by the improved SNP density and whether the associations improved by imputing missing values for genotyped SNPs. The region with the best p-values continued to be the chromosome 4 region near *HHIP*, and the results from imputed genotypes in this region are depicted in Figure 2. The p-value for rs13147758 was slightly better in the results from imputed genotypes, and a total of 27 SNPs in the region (including the four reported in Table 2) met the criterion for genome-wide statistical significance (p<5E-08). The colors plotted in Figure 2 reflect the linkage disequilibrium (LD) of each SNP with rs13147758 and demonstrate the strong LD among the SNPs with the smallest p-values. All SNP results for association with percent predicted FEV_1_/FVC with a p-value<0.001 for LME analysis of high quality imputed genotypes have been included in Table S1 in the Online Data Supplement.

Table 3 reports the association results for the top GWA regions screened in 835 Family Heart Study participants. The SNP with the best overall p-value (rs11100860 on chromosome 4) was ordered for genotyping, but the assay did not meet Applied Biosystems' internal quality control criteria and therefore was not genotyped. SNP rs13147758, with the second best overall p-value, was therefore studied to represent the chromosome 4 region. The SNP on chromosome 5, rs7707619, had a minor allele frequency of 1% in Framingham, and was not observed in the Family Heart Study samples. Of the nine SNPs studied for association with the percent predicted FEV_1_/FVC ratio in the Family Heart Study, only one p-value was observed that met a Bonferroni corrected cutoff for statistical significance (p<0.005), which was located in the chromosome 4 GWA significant region (rs13147758; p-value = 2.0E-04).

**Table 3:** Association results for SNPs in the top 10 GWA regions identified in Framingham and examined in 835 Family Heart Study participants for association to percent predicted FEV_1_/FVC.

rs#	Chrom	position	MAF	Beta estimate	p-value	Gene region
rs13147758	4	145679680	0.411	**0.25**	**2.0E-04**	*HHIP*
rs6471895	8	61787503	0.025	−0.17	0.33	*CHD7*
rs1017444	2	117491841	0.038	−0.38	0.03	
rs17086172	18	68378001	0.071	−0.04	0.77	*CBLN2*
rs7707619	5	72715410	0	*	*	*FOXD1*
rs994960	3	73814606	0.472	−0.12	0.06	*PDZRN3*
rs17646919	22	28730861	0.07	−0.15	0.27	*MTMR3*
rs2304488	16	56631711	0.223	0.04	0.68	*MMP15*
rs573461	7	51403173	0.277	0.06	0.44	*COBL*
rs2456526	15	50876734	0.13	0.07	0.48	*ONECUT1*

*: SNP was genotyped and found to be non-polymorphic in the sample.

To further evaluate the region of chromosome 4 that demonstrated replication with SNP rs13147758, we examined association with additional SNPs and additional phenotypes in the Family Heart Study. Table 4 presents association to FEV_1_, the FEV_1_/FVC ratio, and a dichotomous airflow obstruction trait in the ever-smoking participants. When the analysis was restricted to subjects with a history of ever smoking cigarettes, the association between rs13147758 genotype and the FEV_1_/FVC ratio had a smaller p-value (2.2E-06) and larger estimate of effect. The SNP identified with association to the FEV_1_/FVC ratio phenotype was also statistically significant for FEV_1_ (p = 0.0001) and airflow obstruction (p = 6.18E-06) in smokers. Furthermore, minor alleles at neighboring SNPs exhibited association to lower lung function and increased risk of airflow obstruction (e.g. rs17019336: OR = 1.76, p = 0.004; rs2353397: OR = 1.65, p = 0.003).

**Table 4:** SNP association results for the Family Heart Study smokers (N = 485) in the chromosome 4 region.

SNP	Position	MAF	FEV_1_ beta	FEV_1_ p-value	Ratio beta	Ratio p-value	Airflow Obstruction OR(a)	Airflow Obstruction p-value(a)
rs17019336(b)	145553059	0.23	−0.222	0.052	−0.294	0.01	1.76	0.004
rs13147758	145679680	0.41	0.341	0.0001	0.397	2.24E-06	0.45	6.18E-06
rs2353397	145737028	0.44	−0.154	0.09	−0.138	0.14	1.65	0.003
rs2035901	145741317	0.49	0.082	0.37	0.107	0.26	0.67	0.02
rs6537302	145807566	0.46	0.082	0.38	0.057	0.59	0.69	0.04

(a) N = 185 cases, 226 controls.

(b) SNP had the lowest p-value for FEV_1_ (p = 1.7E-04) in Framingham results for this region.

Table 5 presents the results of the Family Heart Study sample alongside Framingham results for rs13147758 and includes analyses of FEV_1_, a dichotomous airflow obstruction trait, and analyses restricted to smokers. The results demonstrate a modest association with FEV_1_ in the Framingham sample, and a significant association with FEV_1_ in the Family Heart Study. When restricted to ever smokers, the effect size and p-values for FEV_1_ and the ratio improve in the Family Heart sample, but not in Framingham. For analysis of the dichotomous airflow obstruction phenotype, restricting the analysis to ever smokers improves the effect size and p-value in both samples. The minor allele of rs13147758 was protective for airflow obstruction among ever smokers in both samples, though only a trend toward statistical significance was seen in the Framingham sample.

**Table 5:** Association results for rs13147758 with percent predicted FEV_1_/FVC, FEV_1_, and airflow obstruction across studies and in subset of ever smokers.

Phenotype	Framingham Heart Study				Family Heart Study			
	N = 7691		N = 4141 smokers		N = 823		N = 485 smokers	
	Beta est	p-value	Beta est smokers	p-value smokers	Beta est	p-value	Beta est smokers	p-value smokers
FEV_1_/FVC	0.098	2.31E-08	0.09	8.1E-05	0.246	0.0002	0.397	2.2E-06
FEV_1_	0.038	0.03	0.04	0.09	0.196	0.004	0.341	0.0001

	623 cases, 5902 controls		Smokers: 506 cases, 2884 controls		223 cases, 455 controls		Smokers: 185 cases, 226 controls	
	OR all	p-value	OR smokers	p-value	OR all	p-value	OR smokers	p-value
Airflow obstruction	0.9	0.14	0.85	0.06	0.61	0.005	0.45	6.18E-06

## Discussion

The GWA results for percent predicted FEV_1_/FVC in the Framingham Heart Study identified a region on chromosome 4q31 around 145.68 Mb with LME p-values that achieved genome-wide statistical significance (rs13147758 p = 2.31E-08 for genotyped SNP; p = 1.95E-08 for the same SNP imputed). FBAT analyses, which protect against false positive results arising from population stratification, also provided strong support for an association (p = 6.9E-06). Screening the top ten identified regions in 835 Family Heart Study participants further implicated the chromosome 4 region, and did not support the SNP association in the other nine regions. Though the replication sample size may have had limited power to detect SNPs with smaller MAFs or effect sizes, the identification of replication on chromosome 4 is not likely to be a false positive. The minor allele of rs13147758 with a frequency of 39% was associated with higher levels of the FEV_1_/FVC ratio and a 15–55% reduced risk of airflow obstruction among smokers. The reported beta estimates were on the scale of the standardized residual and reflect an approximately 1% increase in the percent of predicted FEV_1_/FVC ratio. For example, among Framingham smokers, the unadjusted mean percent predicted FEV_1_/FVC ratio was 94.4%, 95.5%, and 96.3% for the homozygous major allele, heterozygous, and homozygous minor allele genotypes, respectively.

In a related publication describing results for a GWA of COPD, the chromosome 4q31 region was identified among the top 100 SNP results and replicated in two independent populations [21]. The two SNPs reported with combined p-values of 1.5E-07 and 1.7E-07 both exhibit strong LD with rs13147758 (r^2^ = 0.97 in HapMap CEPH). The independent finding for this region based on samples ascertained for moderate to severe COPD provides compelling evidence for the association on chromosome 4q31.

The genomic region on chromosome 4 does not lend itself to easy interpretation of the SNP association results. The closest characterized gene, hedgehog-interacting protein (*HHIP*), which is located distal to the associated SNPs, is an intriguing candidate because the hedgehog signaling pathway is known to influence lung development [22] and is activated in the airways during repair of injury [23]. However, the association does not explicitly implicate the gene. The specific SNP (rs13147758) that was replicated lies 107 kb 5′ of the start site of *HHIP* and does not exhibit LD with SNPs located within the *HHIP* gene transcript. Another gene, *CR620567*, is transcribed on the opposite strand, and rs13147758 is 104 kb 3′ from the end of that gene. The colored arrows in Figure 2 show the genes′ orientation. Also shown is the presence of an expressed sequence tag (EST) in the same region. Three overlapping ESTs transcribed on the opposite strand, including W05107, BX104541 and AI822050 (labeled in Figure 2 only as BX104541), extend between 225–300 kb in length and were cloned from a Soares fetal lung library (NbHL19W) [24],[25]. The location of the association leaves open the possibility for regulatory effects on *HHIP*, the uncharacterized ESTs from fetal lung, or members of the *GYP* gene cluster.

Though located nearly 400 kilobase pairs from the associated SNPs, the gene glycophorin A (*GYPA*) is the nearest known proximal gene and lies adjacent to its homolog *GYPB*. Glycophorins are proteins of the red blood cell membrane, and glycophorin expression has been shown to be lower among COPD patients than controls [26]. The red blood cell is susceptible to oxidative alterations, which have been proposed as a useful marker for cell damage in COPD [27]. Review of the imputed data results for a missense SNP in *GYPA* showed a modest association (rs7658293; p = 0.037), with a common minor allele associated with a decreased FEV_1_/FVC ratio, and additional SNPs in LD exhibited similar p-values. However, the *GYPA* missense SNP does not exhibit LD with the replicated SNP rs13147758 (HapMap CEPH r^2^ = 0.006). In contrast to SNP results within *GYPA*, none of the SNPs within *HHIP* had p-values less than 0.05 for association with percent predicted FEV_1_/FVC.

The GWA results provide only limited insight into the functional role of the most strongly associated region. It is not clear if the chromosome 4 region is associated with pulmonary disease or variability in pulmonary function in normal populations. The definitions of airflow obstruction and asthma in these observational studies have limitations. Both studies use spirometry to define airflow obstruction, but post-bronchodilator spirometry is not available, which may misclassify some individuals with reversible airflow obstruction as seen in asthma. However, results excluding diagnosed asthmatic participants (data not shown) suggest that the association is not driven by an asthma phenotype. GWA analyses for the FEV_1_/FVC ratio after exclusion of self-reported asthma identified the same chromosome 4 region with the best p-value at genome-wide significance, and all the results in the Family Heart Study represent a non-asthmatic sample. Though we have not identified a functional variant in the region, the replication of genome-wide significant results suggests that this intergenic region of chromosome 4 may influence pulmonary function and risk of obstructive pulmonary disease.

The related manuscript describing a GWA for COPD identified association to a region of chromosome 15 that includes the nicotinic acetylcholine receptor (*CHRNA3*/*5*) [21]. Two SNPs were identified to be associated with COPD in the discovery cohort and replicated in two independent populations. We evaluated whether these SNPs (rs8034191 and rs1051730) provided evidence of association using the Framingham imputed genotypes, as they were not directly genotyped, but neither SNP was significantly associated with percent predicted FEV_1_/FVC or dichotomous airflow obstruction. Reviewing results across the region, we noted that SNPs in the nearby *IREB2* gene provided evidence for association to percent predicted FEV_1_/FVC (best p-value = 0.006). This lack of a specific replication to *CHRNA3*/*5* may reflect differences in the severity of airflow obstruction present in the population based Framingham study in contrast to a clinically ascertained COPD case sample. Further, as this locus has been reported to be associated with nicotine dependence [28],[29], differences in smoking behavior may influence the power to detect an association. As the Framingham study represents three generations ascertained at different time points, secular trends in smoking behavior may confound the association despite adjustment for pack-years within cohort.

We have provided, in the online supplement, a resource of all SNP association results with nominal statistical significance (p<0.001) for the percent predicted FEV_1_/FVC to facilitate future efforts to replicate SNP association with pulmonary phenotypes. The region of association on chromosome 4 warrants follow-up to explore the expressed sequence in fetal and adult lung, and to evaluate the relationship of the associated SNPs to gene expression of the nearby genes *HHIP* and *GYPA* and the ESTs. Our results demonstrate the utility of genome-wide association analysis in the identification of genetic determinants of obstructive lung diseases.

## Supporting Information

Table S1 All SNPs generating a p-value<0.001 for LME analysis of high quality (ratio>0.9) imputed genotypes for percent predicted FEV_1_/FVC in an additive genetic model.
(4.81 MB DOC)
