# Supplementary material for: A Genome-Wide Association Study of Pulmonary Function Measures in the Framingham Heart Study
Source: PLoS Genet. 2009 Mar 20;5(3):e1000429. doi: 10.1371/journal.pgen.1000429 (PMC2652834; doi:10.1371/journal.pgen.1000429)
Supplement: Table S1 — All SNPs generating a p-value<0.001 for LME analysis of high quality (ratio>0.9) imputed genotypes for percent predicted FEV1/FVC in an additive genetic model. (4.81 MB DOC) [file pgen.1000429.s001.doc]

**Online Supplement**

The results of the GWA for percent predicted FEV­_1_/FVC using imputed SNP genotypes were evaluated to determine whether new regions were implicated by the improved SNP density and whether the associations improved by imputing missing values for the genotyped SNPs. Out of 2,540,223 imputed SNPs, a total of 84 SNPs with high quality imputation had p-values less than 1.5E-05, the cutoff that defined the ten regions of interest among genotyped SNPs. Only one region not previously identified in Table 2 was revealed by the analysis of imputed SNPs. Three SNPs in *ZBTB20* (zinc finger and BTB domain containing 20) with a 40% minor allele frequency were associated with lower percent predicted FEV­_1_/FVC with p-values of 6E-06. The region with the best p-values continued to be the chromosome 4 region near *HHIP*, and the results from imputed genotypes in this region are depicted in Figure 2. The p-value for rs13147758 was slightly better in the results from imputed genotypes, and a total of 27 SNPs in the region (including the four reported in Table 2) met the criterion for genome-wide statistical significance (p<5E-08). The imputed GWA results also implicated the *CHD7*, *CBLN2,* *PDZRN3*, *ONECUT1*, and *MMP15* regions that were identified using genotyped SNPs (Table 2). All SNP results for association with percent predicted FEV­_1_/FVC with a p-value < 0.001 for LME analysis of high quality imputed genotypes have been included in Table S1.

Table S1. All SNPs generating a p-value < 0.001 for LME analysis of high quality (ratio > 0.9) imputed genotypes for percent predicted FEV_1_/FVC in an additive genetic model. The imputed minor allele frequency (iMAF), minor and major allele, whether a SNP’s observed genotype was used for imputation (Input: Y/N), and the closest gene is provided for each SNP result.

| rs# | Chr | Position | iMAF | Beta | p-value | Minor | Major | Input | Closest gene |
| --- | --- | --- | --- | --- | --- | --- | --- | --- | --- |
| rs1512281 | 4 | 145654351 | 0.413 | 0.101 | 1.67E-08 | G | A | N | *HHIP* |
| rs12509311 | 4 | 145698112 | 0.385 | 0.099 | 1.73E-08 | T | C | N | *HHIP* |
| rs13147758 | 4 | 145679680 | 0.388 | 0.098 | 1.95E-08 | G | A | Y | *HHIP* |
| rs13148031 | 4 | 145679788 | 0.388 | 0.098 | 1.97E-08 | G | A | N | *HHIP* |
| rs720484 | 4 | 145681814 | 0.388 | 0.098 | 1.99E-08 | A | G | N | *HHIP* |
| rs720485 | 4 | 145682038 | 0.388 | 0.098 | 2.01E-08 | C | A | N | *HHIP* |
| rs6828540 | 4 | 145682681 | 0.388 | 0.098 | 2.03E-08 | A | G | N | *HHIP* |
| rs6537292 | 4 | 145689418 | 0.390 | 0.098 | 2.04E-08 | T | G | N | *HHIP* |
| rs2130339 | 4 | 145684335 | 0.388 | 0.098 | 2.06E-08 | G | A | N | *HHIP* |
| rs6830832 | 4 | 145684941 | 0.388 | 0.098 | 2.07E-08 | G | T | N | *HHIP* |
| rs12511230 | 4 | 145690695 | 0.390 | 0.098 | 2.08E-08 | T | A | N | *HHIP* |
| rs11938745 | 4 | 145685218 | 0.388 | 0.098 | 2.09E-08 | C | T | N | *HHIP* |
| rs7697189 | 4 | 145656344 | 0.394 | 0.098 | 2.09E-08 | C | G | N | *HHIP* |
| rs12504628 | 4 | 145655774 | 0.394 | 0.098 | 2.11E-08 | C | T | N | *HHIP* |
| rs1489759 | 4 | 145693923 | 0.390 | 0.098 | 2.17E-08 | G | A | N | *HHIP* |
| rs995758 | 4 | 145697651 | 0.390 | 0.098 | 2.23E-08 | T | C | N | *HHIP* |
| rs1489762 | 4 | 145674414 | 0.388 | 0.098 | 2.30E-08 | C | T | N | *HHIP* |
| rs1980057 | 4 | 145705188 | 0.390 | 0.098 | 2.33E-08 | T | C | Y | *HHIP* |
| rs1828591 | 4 | 145700230 | 0.390 | 0.098 | 2.35E-08 | G | A | N | *HHIP* |
| rs6842889 | 4 | 145699330 | 0.390 | 0.098 | 2.38E-08 | C | T | N | *HHIP* |
| rs7655625 | 4 | 145705365 | 0.390 | 0.098 | 2.38E-08 | C | T | Y | *HHIP* |
| rs13118928 | 4 | 145705839 | 0.390 | 0.098 | 2.39E-08 | G | A | N | *HHIP* |
| rs11100860 | 4 | 145698589 | 0.390 | 0.098 | 2.39E-08 | G | A | Y | *HHIP* |
| rs6537296 | 4 | 145707384 | 0.390 | 0.098 | 2.40E-08 | G | A | N | *HHIP* |
| rs1512288 | 4 | 145710731 | 0.390 | 0.098 | 2.43E-08 | A | G | N | *HHIP* |
| rs6817273 | 4 | 145711453 | 0.390 | 0.098 | 2.44E-08 | C | T | N | *HHIP* |
| rs1032297 | 4 | 145654194 | 0.407 | 0.098 | 3.11E-08 | G | A | N | *HHIP* |
| rs13141641 | 4 | 145725906 | 0.396 | 0.096 | 8.40E-08 | C | T | N | *HHIP* |
| rs6471895 | 8 | 61787503 | 0.038 | -0.214 | 1.34E-06 | C | T | Y | *CHD7* |
| rs13269361 | 8 | 61770005 | 0.038 | -0.214 | 1.40E-06 | G | A | N | *CHD7* |
| rs16926398 | 8 | 61769299 | 0.038 | -0.214 | 1.41E-06 | A | G | N | *CHD7* |
| rs7013969 | 8 | 61760001 | 0.038 | -0.214 | 1.48E-06 | A | G | N | *CHD7* |
| rs16926379 | 8 | 61758160 | 0.037 | -0.222 | 2.02E-06 | G | A | N | *CHD7* |
| rs4835637 | 4 | 145658477 | 0.476 | -0.083 | 2.20E-06 | A | G | N | *HHIP* |
| rs17086172 | 18 | 68378001 | 0.057 | -0.173 | 2.42E-06 | C | T | Y | *CBLN2* |
| rs17086174 | 18 | 68379613 | 0.057 | -0.173 | 2.43E-06 | C | G | N | *CBLN2* |
| rs640733 | 18 | 68380416 | 0.057 | -0.173 | 2.45E-06 | C | T | N | *CBLN2* |
| rs13142879 | 4 | 145629927 | 0.382 | 0.084 | 2.53E-06 | T | C | N | *HHIP* |
| rs2130499 | 4 | 145608284 | 0.383 | 0.084 | 2.53E-06 | T | G | N | *HHIP* |
| rs681058 | 18 | 68396951 | 0.062 | -0.167 | 2.59E-06 | C | T | N | *CBLN2* |
| rs1505770 | 4 | 145600252 | 0.384 | 0.084 | 2.63E-06 | C | T | N | *HHIP* |
| rs1032296 | 4 | 145654138 | 0.445 | -0.083 | 2.87E-06 | T | C | N | *HHIP* |
| rs654316 | 18 | 68398275 | 0.057 | -0.170 | 3.33E-06 | A | G | N | *CBLN2* |
| rs1022170 | 3 | 73787987 | 0.434 | -0.080 | 3.46E-06 | C | A | N | *PDZRN3* |
| rs2036893 | 3 | 73788712 | 0.434 | -0.080 | 3.47E-06 | A | G | N | *PDZRN3* |
| rs1512285 | 4 | 145670409 | 0.462 | -0.078 | 4.34E-06 | A | G | Y | *HHIP* |
| rs6829956 | 4 | 145659738 | 0.462 | -0.078 | 4.39E-06 | C | T | N | *HHIP* |
| rs7681384 | 4 | 145656464 | 0.462 | -0.078 | 4.43E-06 | T | C | N | *HHIP* |
| rs994960 | 3 | 73814606 | 0.482 | -0.078 | 4.53E-06 | G | A | Y | *PDZRN3* |
| rs9790092 | 3 | 73814228 | 0.482 | -0.077 | 4.63E-06 | G | A | N | *PDZRN3* |
| rs9790264 | 3 | 73813996 | 0.490 | -0.078 | 5.04E-06 | C | T | N | *PDZRN3* |
| rs6777769 | 3 | 73818048 | 0.482 | -0.077 | 5.21E-06 | C | G | N | *PDZRN3* |
| rs2456526 | 15 | 50876734 | 0.137 | -0.113 | 5.24E-06 | C | T | Y | *ONECUT1* |
| rs7678427 | 4 | 145653025 | 0.372 | 0.081 | 5.46E-06 | A | C | N | *HHIP* |
| rs2456525 | 15 | 50867705 | 0.137 | -0.113 | 5.50E-06 | T | C | N | *ONECUT1* |
| rs1512282 | 4 | 145650947 | 0.371 | 0.081 | 5.53E-06 | G | A | N | *HHIP* |
| rs973796 | 4 | 145643418 | 0.370 | 0.081 | 5.56E-06 | C | A | Y | *HHIP* |
| rs17614981 | 3 | 115592234 | 0.400 | -0.082 | 5.95E-06 | A | C | N | *ZBTB20* |
| rs11918578 | 3 | 73818811 | 0.483 | -0.077 | 6.04E-06 | G | A | N | *PDZRN3* |
| rs9859157 | 3 | 115593236 | 0.400 | -0.082 | 6.06E-06 | A | C | N | *ZBTB20* |
| rs1432077 | 18 | 68423879 | 0.065 | -0.159 | 6.13E-06 | T | G | N | *CBLN2* |
| rs9859304 | 3 | 115593319 | 0.400 | -0.082 | 6.15E-06 | T | C | N | *ZBTB20* |
| rs2440376 | 15 | 50879162 | 0.140 | -0.112 | 6.60E-06 | A | G | N | *ONECUT1* |
| rs1567574 | 3 | 73820157 | 0.483 | -0.077 | 6.63E-06 | T | C | N | *PDZRN3* |
| rs2456532 | 15 | 50879812 | 0.140 | -0.112 | 6.74E-06 | C | G | N | *ONECUT1* |
| rs2243790 | 15 | 50879912 | 0.140 | -0.112 | 6.82E-06 | A | G | N | *ONECUT1* |
| rs1877252 | 3 | 73810230 | 0.484 | -0.076 | 6.98E-06 | T | C | Y | *PDZRN3* |
| rs4677328 | 3 | 73802634 | 0.484 | -0.076 | 7.67E-06 | A | T | N | *PDZRN3* |
| rs2165990 | 15 | 50857039 | 0.282 | -0.086 | 9.12E-06 | G | A | Y | *ONECUT1* |
| rs9926547 | 16 | 56639295 | 0.197 | -0.098 | 9.14E-06 | T | C | N | *MMP15* |
| rs10518694 | 15 | 50859965 | 0.138 | -0.110 | 9.35E-06 | A | C | N | *ONECUT1* |
| rs11128357 | 3 | 73791220 | 0.454 | -0.075 | 1.01E-05 | C | A | N | *PDZRN3* |
| rs2137064 | 3 | 73833683 | 0.474 | -0.075 | 1.01E-05 | G | A | Y | *PDZRN3* |
| rs9868151 | 3 | 73834122 | 0.474 | -0.075 | 1.02E-05 | C | A | N | *PDZRN3* |
| rs12447440 | 16 | 56642671 | 0.165 | -0.102 | 1.14E-05 | T | G | N | *MMP15* |
| rs2118018 | 16 | 56633923 | 0.202 | -0.092 | 1.22E-05 | G | A | N | *MMP15* |
| rs2304488 | 16 | 56631711 | 0.202 | -0.092 | 1.29E-05 | G | A | Y | *MMP15* |
| rs1050779 | 16 | 56637716 | 0.193 | -0.094 | 1.46E-05 | C | G | N | *MMP15* |
| rs3743563 | 16 | 56636666 | 0.193 | -0.094 | 1.48E-05 | A | G | N | *MMP15* |
| rs1858325 | 3 | 73793062 | 0.447 | -0.074 | 1.48E-05 | A | G | N | *PDZRN3* |
| rs4677325 | 3 | 73793397 | 0.446 | -0.074 | 1.49E-05 | G | A | N | *PDZRN3* |
| rs4677326 | 3 | 73793442 | 0.446 | -0.074 | 1.49E-05 | G | C | N | *PDZRN3* |
| rs9310279 | 3 | 73793635 | 0.446 | -0.074 | 1.50E-05 | C | T | N | *PDZRN3* |
| rs12447804 | 16 | 56632783 | 0.193 | -0.093 | 1.50E-05 | T | C | N | *MMP15* |
| rs9824294 | 3 | 73794325 | 0.446 | -0.074 | 1.51E-05 | T | C | N | *PDZRN3* |
| rs1511521 | 3 | 73794553 | 0.446 | -0.074 | 1.52E-05 | T | C | N | *PDZRN3* |
| rs12340660 | 9 | 79353634 | 0.073 | 0.141 | 1.60E-05 | A | G | Y | *GNA14* |
| rs4494867 | 3 | 73838093 | 0.464 | -0.073 | 1.64E-05 | T | C | N | *PDZRN3* |
| rs12499685 | 4 | 145585950 | 0.301 | 0.082 | 1.67E-05 | C | T | N | *HHIP* |
| rs17019354 | 4 | 145586427 | 0.301 | 0.082 | 1.67E-05 | T | G | N | *HHIP* |
| rs1039956 | 3 | 73797533 | 0.465 | -0.073 | 1.72E-05 | T | A | N | *PDZRN3* |
| rs9847694 | 3 | 73794315 | 0.435 | -0.075 | 1.77E-05 | A | T | N | *PDZRN3* |
| rs1543070 | 3 | 73831927 | 0.486 | -0.073 | 1.82E-05 | T | C | N | *PDZRN3* |
| rs6821114 | 4 | 145671839 | 0.456 | -0.073 | 1.93E-05 | A | G | Y | *HHIP* |
| rs1355467 | 3 | 73796830 | 0.466 | -0.072 | 2.02E-05 | T | C | Y | *PDZRN3* |
| rs11598305 | 10 | 12309406 | 0.365 | 0.075 | 2.05E-05 | A | C | Y | *CDC123* |
| rs4835177 | 4 | 145493378 | 0.347 | 0.076 | 2.18E-05 | G | A | Y | *GYPA* |
| rs2900115 | 5 | 117442249 | 0.095 | 0.123 | 2.22E-05 | A | G | Y | *DTWD2* |
| rs1039955 | 3 | 73798754 | 0.457 | -0.072 | 2.35E-05 | T | C | N | *PDZRN3* |
| rs1489761 | 4 | 145674941 | 0.462 | -0.072 | 2.37E-05 | G | A | N | *HHIP* |
| rs17113606 | 10 | 90599114 | 0.014 | 0.314 | 2.37E-05 | G | A | Y | *ANKRD22* |
| rs6845536 | 4 | 145672233 | 0.462 | -0.072 | 2.47E-05 | C | T | Y | *HHIP* |
| rs4271183 | 1 | 20035877 | 0.071 | 0.146 | 2.48E-05 | G | T | N | *RNF186* |
| rs1505766 | 4 | 145527749 | 0.310 | 0.078 | 2.48E-05 | T | C | N | *GYPA* |
| rs17646919 | 22 | 28730861 | 0.085 | 0.128 | 2.56E-05 | G | A | Y | *MTMR3* |
| rs1259578 | 10 | 76762032 | 0.374 | 0.076 | 2.60E-05 | G | A | N | *ZNF503* |
| rs2440322 | 15 | 50859038 | 0.138 | -0.103 | 2.60E-05 | G | T | N | *ONECUT1* |
| rs2435884 | 10 | 76764224 | 0.374 | 0.076 | 2.60E-05 | G | A | N | *ZNF503* |
| rs12499537 | 4 | 145567548 | 0.323 | 0.079 | 2.63E-05 | T | G | N | *HHIP* |
| rs6828795 | 4 | 145594210 | 0.315 | 0.079 | 2.64E-05 | T | C | N | *HHIP* |
| rs12510916 | 4 | 145591698 | 0.316 | 0.079 | 2.67E-05 | T | C | N | *HHIP* |
| rs13142439 | 4 | 145590886 | 0.316 | 0.079 | 2.67E-05 | G | A | N | *HHIP* |
| rs7692102 | 4 | 145676591 | 0.464 | -0.072 | 2.68E-05 | A | G | N | *HHIP* |
| rs1394998 | 4 | 145521605 | 0.310 | 0.077 | 2.70E-05 | T | C | N | *GYPA* |
| rs12137940 | 1 | 20037970 | 0.070 | 0.144 | 2.72E-05 | A | C | N | *RNF186* |
| rs9926693 | 16 | 56654858 | 0.212 | -0.088 | 2.74E-05 | G | A | N | *MMP15* |
| rs17766287 | 4 | 145498287 | 0.309 | 0.077 | 2.75E-05 | C | T | N | *GYPA* |
| rs2070600 | 6 | 32259421 | 0.043 | 0.176 | 2.76E-05 | T | C | Y | *AGER* |
| rs4290852 | 4 | 145555922 | 0.309 | 0.077 | 2.77E-05 | T | C | N | *HHIP* |
| rs4240362 | 4 | 145505813 | 0.309 | 0.077 | 2.78E-05 | C | G | N | *GYPA* |
| rs12500355 | 4 | 145541272 | 0.309 | 0.077 | 2.79E-05 | C | T | N | *HHIP* |
| rs17019340 | 4 | 145553233 | 0.309 | 0.077 | 2.83E-05 | A | C | Y | *HHIP* |
| rs11593567 | 10 | 12347969 | 0.560 | 0.076 | 2.85E-05 | G | A | N | *CDC123* |
| rs2831873 | 21 | 28804530 | 0.175 | -0.094 | 2.88E-05 | A | G | N | *N6AMT1* |
| rs2831887 | 21 | 28816416 | 0.176 | -0.093 | 2.94E-05 | T | C | N | *N6AMT1* |
| rs2831877 | 21 | 28807057 | 0.176 | -0.094 | 2.94E-05 | C | A | N | *N6AMT1* |
| rs7654571 | 4 | 145540456 | 0.239 | -0.085 | 2.98E-05 | A | G | N | *HHIP* |
| rs225206 | 17 | 27918485 | 0.512 | 0.070 | 2.99E-05 | G | T | Y | *MYO1D* |
| rs10917558 | 1 | 20046559 | 0.070 | 0.142 | 2.99E-05 | T | C | N | *RNF186* |
| rs17766168 | 4 | 145492142 | 0.309 | 0.077 | 3.01E-05 | A | G | N | *GYPA* |
| rs6834183 | 4 | 145490405 | 0.309 | 0.077 | 3.01E-05 | C | T | N | *GYPA* |
| rs17709487 | 4 | 145489514 | 0.309 | 0.077 | 3.02E-05 | T | G | Y | *GYPA* |
| rs11234778 | 11 | 86136751 | 0.158 | -0.098 | 3.03E-05 | T | C | N | *PRSS23* |
| rs7180600 | 15 | 50857433 | 0.139 | -0.102 | 3.04E-05 | A | G | Y | *ONECUT1* |
| rs1926185 | 10 | 90632522 | 0.016 | 0.285 | 3.14E-05 | C | A | N | *STAMBPL1* |
| rs11602353 | 11 | 86134812 | 0.158 | -0.098 | 3.15E-05 | T | C | N | *PRSS23* |
| rs7776375 | 6 | 142818757 | 0.285 | 0.080 | 3.21E-05 | G | A | N | *GPR126* |
| rs12269097 | 10 | 90634715 | 0.016 | 0.284 | 3.26E-05 | T | G | N | *STAMBPL1* |
| rs12924451 | 16 | 56651170 | 0.197 | -0.088 | 3.34E-05 | T | G | N | *MMP15* |
| rs17262657 | 1 | 29964236 | 0.410 | -0.074 | 3.39E-05 | C | T | N | *PTPRU* |
| rs12929002 | 16 | 56643383 | 0.197 | -0.088 | 3.40E-05 | A | G | N | *MMP15* |
| rs13105210 | 4 | 145482377 | 0.313 | 0.078 | 3.45E-05 | T | C | N | *GYPA* |
| rs2241773 | 16 | 56638411 | 0.197 | -0.088 | 3.45E-05 | T | C | N | *MMP15* |
| rs1866967 | 1 | 29958249 | 0.410 | -0.073 | 3.49E-05 | G | A | N | *PTPRU* |
| rs737903 | 22 | 28673846 | 0.085 | 0.128 | 3.63E-05 | A | T | N | *MTMR3* |
| rs7843452 | 8 | 104249919 | 0.031 | 0.205 | 3.70E-05 | G | T | N | *BAALC* |
| rs7839419 | 8 | 104245562 | 0.031 | 0.202 | 3.70E-05 | G | A | Y | *BAALC* |
| rs7843029 | 8 | 104249641 | 0.031 | 0.205 | 3.71E-05 | C | T | N | *BAALC* |
| rs11202880 | 10 | 90637622 | 0.016 | 0.280 | 3.78E-05 | G | A | N | *STAMBPL1* |
| rs2392015 | 7 | 31997405 | 0.203 | 0.089 | 3.90E-05 | T | C | N | *PDE1C* |
| rs9373424 | 6 | 145073553 | 0.103 | 0.120 | 3.93E-05 | G | A | N | *UTRN* |
| rs1876089 | 3 | 80438463 | 0.247 | -0.083 | 3.97E-05 | C | T | N | *ROBO1* |
| rs11202881 | 10 | 90641681 | 0.016 | 0.279 | 4.08E-05 | C | A | N | *STAMBPL1* |
| rs9376844 | 6 | 145067304 | 0.103 | 0.119 | 4.16E-05 | C | T | N | *UTRN* |
| rs7075596 | 10 | 90649566 | 0.016 | 0.281 | 4.23E-05 | A | G | N | *STAMBPL1* |
| rs12019525 | 13 | 24226680 | 0.358 | -0.072 | 4.26E-05 | G | A | Y | *RNF17* |
| rs7910708 | 10 | 90644640 | 0.016 | 0.277 | 4.33E-05 | T | C | Y | *STAMBPL1* |
| rs7076879 | 10 | 90642492 | 0.016 | 0.277 | 4.33E-05 | G | C | Y | *STAMBPL1* |
| rs1497956 | 4 | 167329038 | 0.512 | -0.071 | 4.34E-05 | A | G | N | *TLL1* |
| rs17713999 | 22 | 28714053 | 0.084 | 0.126 | 4.41E-05 | G | A | N | *MTMR3* |
| rs949794 | 4 | 167326473 | 0.522 | -0.069 | 4.55E-05 | T | C | Y | *TLL1* |
| rs6124112 | 20 | 37187116 | 0.273 | 0.079 | 4.60E-05 | C | T | N | *DHX35* |
| rs12493973 | 3 | 171510691 | 0.379 | -0.071 | 4.61E-05 | G | A | Y | *PRKCI* |
| rs4763513 | 12 | 18987475 | 0.390 | 0.072 | 4.75E-05 | C | T | N | *PLEKHA5* |
| rs2440315 | 15 | 50871645 | 0.239 | -0.081 | 4.76E-05 | G | T | N | *ONECUT1* |
| rs4691248 | 4 | 167319400 | 0.523 | -0.069 | 4.76E-05 | G | C | N | *TLL1* |
| rs6838888 | 4 | 167319021 | 0.477 | 0.069 | 4.77E-05 | A | G | N | *TLL1* |
| rs4763514 | 12 | 18987834 | 0.390 | 0.072 | 4.83E-05 | T | A | N | *PLEKHA5* |
| rs2120450 | 15 | 50888872 | 0.135 | -0.102 | 4.85E-05 | A | T | N | *ONECUT1* |
| rs2456531 | 15 | 50879188 | 0.242 | -0.081 | 4.88E-05 | C | A | N | *ONECUT1* |
| rs1259594 | 10 | 76778693 | 0.365 | 0.073 | 4.91E-05 | G | A | N | *ZNF503* |
| rs4799709 | 18 | 29509869 | 0.083 | 0.127 | 4.92E-05 | C | T | N | *ASXL3* |
| rs1899752 | 15 | 50880229 | 0.242 | -0.081 | 4.94E-05 | G | A | N | *ONECUT1* |
| rs8090361 | 18 | 29517990 | 0.070 | 0.136 | 5.03E-05 | G | C | Y | *ASXL3* |
| rs703719 | 12 | 100191681 | 0.428 | 0.071 | 5.07E-05 | C | T | N | *UTP20* |
| rs17733794 | 18 | 29519467 | 0.070 | 0.136 | 5.08E-05 | T | G | N | *ASXL3* |
| rs13096120 | 3 | 73838775 | 0.478 | -0.069 | 5.12E-05 | G | A | N | *PDZRN3* |
| rs7227148 | 18 | 29520293 | 0.070 | 0.136 | 5.12E-05 | G | C | N | *ASXL3* |
| rs4799708 | 18 | 29509827 | 0.069 | 0.137 | 5.23E-05 | G | T | N | *ASXL3* |
| rs12597233 | 16 | 56657709 | 0.189 | -0.087 | 5.31E-05 | A | G | Y | *MMP15* |
| rs2022059 | 6 | 32264467 | 0.046 | 0.169 | 5.33E-05 | C | G | N | *PBX2* |
| rs2856437 | 6 | 32265342 | 0.046 | 0.169 | 5.36E-05 | A | G | N | *PBX2* |
| rs8098239 | 18 | 29507636 | 0.069 | 0.137 | 5.47E-05 | A | G | N | *ASXL3* |
| rs1360194 | 6 | 142794288 | 0.302 | 0.074 | 5.57E-05 | A | G | Y | *GPR126* |
| rs10859824 | 12 | 93953563 | 0.256 | -0.078 | 5.78E-05 | A | G | Y | *NR2C1* |
| rs11820294 | 11 | 97006263 | 0.076 | -0.132 | 5.84E-05 | C | T | N | *JRKL* |
| rs6570510 | 6 | 142796717 | 0.301 | 0.074 | 5.85E-05 | T | C | N | *GPR126* |
| rs977689 | 21 | 28844261 | 0.182 | -0.090 | 5.91E-05 | T | G | N | *N6AMT1* |
| rs6988200 | 8 | 61802606 | 0.026 | -0.216 | 5.98E-05 | T | G | N | *CHD7* |
| rs4143503 | 12 | 18994492 | 0.389 | 0.070 | 6.15E-05 | T | C | N | *PLEKHA5* |
| rs755049 | 10 | 90660790 | 0.013 | 0.312 | 6.24E-05 | G | A | N | *STAMBPL1* |
| rs7068966 | 10 | 12317998 | 0.536 | 0.071 | 6.42E-05 | T | C | N | *CDC123* |
| rs1160758 | 7 | 31918138 | 0.151 | 0.095 | 6.45E-05 | G | C | N | *PDE1C* |
| rs12117495 | 1 | 20056032 | 0.062 | 0.140 | 6.54E-05 | G | A | N | *OTUD3* |
| rs13223209 | 7 | 31916288 | 0.150 | 0.095 | 6.56E-05 | G | A | Y | *PDE1C* |
| rs17758195 | 11 | 86130556 | 0.169 | -0.092 | 6.57E-05 | C | T | N | *PRSS23* |
| rs262121 | 6 | 142881191 | 0.306 | 0.073 | 6.61E-05 | C | T | Y | *GPR126* |
| rs1447050 | 4 | 56397752 | 0.440 | 0.068 | 6.61E-05 | A | G | N | *EXOC1* |
| rs4778640 | 15 | 79391186 | 0.038 | 0.177 | 6.62E-05 | G | A | N | *STARD5* |
| rs11257613 | 10 | 12324398 | 0.536 | 0.071 | 6.63E-05 | A | G | N | *CDC123* |
| rs12365944 | 11 | 86129668 | 0.169 | -0.092 | 6.64E-05 | A | G | N | *PRSS23* |
| rs2488135 | 10 | 18373150 | 0.299 | 0.077 | 6.70E-05 | T | C | N | *SLC39A12* |
| rs2340392 | 3 | 80440990 | 0.236 | -0.080 | 6.71E-05 | A | G | N | *ROBO1* |
| rs12362740 | 11 | 86154338 | 0.145 | -0.097 | 6.73E-05 | T | A | N | *PRSS23* |
| rs262114 | 6 | 142859911 | 0.306 | 0.073 | 6.82E-05 | T | C | Y | *GPR126* |
| rs17150692 | 7 | 30032485 | 0.019 | -0.246 | 6.89E-05 | A | G | N | *FKBP14* |
| rs10998724 | 10 | 70768382 | 0.064 | 0.142 | 6.91E-05 | G | T | N | *HK1* |
| rs3748069 | 6 | 142809326 | 0.299 | 0.074 | 6.92E-05 | G | A | N | *GPR126* |
| rs17098576 | 10 | 120988240 | 0.016 | 0.269 | 7.02E-05 | G | A | Y | *GRK5* |
| rs7585613 | 2 | 151449611 | 0.087 | -0.120 | 7.08E-05 | T | C | N | *RBM43* |
| rs7558214 | 2 | 151449652 | 0.087 | -0.120 | 7.09E-05 | A | G | N | *RBM43* |
| rs2119568 | 15 | 69452878 | 0.164 | -0.092 | 7.10E-05 | C | T | N | *LOC645296* |
| rs16828833 | 2 | 151451744 | 0.087 | -0.120 | 7.10E-05 | T | G | N | *RBM43* |
| rs16828835 | 2 | 151452698 | 0.087 | -0.120 | 7.12E-05 | A | T | N | *RBM43* |
| rs1567573 | 3 | 73820043 | 0.405 | -0.069 | 7.13E-05 | G | T | N | *PDZRN3* |
| rs2190367 | 2 | 151458080 | 0.086 | -0.120 | 7.16E-05 | T | C | N | *RBM43* |
| rs16828857 | 2 | 151461794 | 0.087 | -0.120 | 7.19E-05 | A | C | N | *RBM43* |
| rs17150688 | 7 | 30032430 | 0.019 | -0.246 | 7.22E-05 | G | A | N | *FKBP14* |
| rs7570251 | 2 | 151460575 | 0.086 | -0.120 | 7.26E-05 | C | T | Y | *RBM43* |
| rs2488136 | 10 | 18374527 | 0.295 | 0.078 | 7.33E-05 | A | G | N | *SLC39A12* |
| rs9373346 | 6 | 142788685 | 0.301 | 0.073 | 7.67E-05 | A | G | N | *GPR126* |
| rs10916668 | 1 | 20105673 | 0.063 | 0.138 | 7.82E-05 | A | G | Y | *OTUD3* |
| rs10492999 | 1 | 20110347 | 0.063 | 0.138 | 7.83E-05 | G | A | N | *OTUD3* |
| rs9376841 | 6 | 145054562 | 0.104 | 0.112 | 7.84E-05 | G | A | N | *UTRN* |
| rs2712557 | 12 | 126210339 | 0.103 | -0.109 | 7.84E-05 | T | C | Y | *TMEM132B* |
| rs10830242 | 11 | 87500294 | 0.323 | 0.074 | 7.90E-05 | T | C | N | *RAB38* |
| rs16828865 | 2 | 151462848 | 0.086 | -0.119 | 7.91E-05 | C | T | N | *RBM43* |
| rs6673417 | 1 | 29961195 | 0.360 | -0.073 | 7.94E-05 | C | T | N | *PTPRU* |
| rs4949268 | 1 | 29959543 | 0.360 | -0.073 | 8.03E-05 | C | T | N | *PTPRU* |
| rs1652713 | 10 | 76782306 | 0.354 | 0.070 | 8.13E-05 | T | C | Y | *ZNF503* |
| rs7306990 | 12 | 5851666 | 0.028 | 0.207 | 8.20E-05 | C | G | N | *TMEM16B* |
| rs10817689 | 9 | 116747920 | 0.435 | -0.069 | 8.33E-05 | C | T | N | *TNFSF8* |
| rs7135976 | 12 | 6056377 | 0.500 | 0.067 | 8.34E-05 | C | T | Y | *VWF* |
| rs2238104 | 12 | 6057926 | 0.480 | 0.069 | 8.34E-05 | T | G | N | *VWF* |
| rs9390194 | 6 | 145052836 | 0.104 | 0.111 | 8.38E-05 | A | G | N | *UTRN* |
| rs881172 | 9 | 116754659 | 0.434 | -0.069 | 8.40E-05 | T | C | N | *TNFSF8* |
| rs12908092 | 15 | 69459807 | 0.163 | -0.091 | 8.41E-05 | C | T | N | *LOC645296* |
| rs6498382 | 16 | 13161027 | 0.441 | 0.067 | 8.43E-05 | T | C | Y | *FLJ11151* |
| rs10982467 | 9 | 116751528 | 0.434 | -0.069 | 8.43E-05 | T | C | N | *TNFSF8* |
| rs7601783 | 2 | 151466488 | 0.086 | -0.119 | 8.46E-05 | A | G | N | *RBM43* |
| rs7602001 | 2 | 151466668 | 0.086 | -0.119 | 8.46E-05 | A | G | N | *RBM43* |
| rs9403580 | 6 | 145048473 | 0.104 | 0.111 | 8.47E-05 | C | T | Y | *UTRN* |
| rs17451453 | 3 | 80436231 | 0.236 | -0.079 | 8.48E-05 | C | G | N | *ROBO1* |
| rs16828883 | 2 | 151464957 | 0.086 | -0.119 | 8.49E-05 | T | C | N | *RBM43* |
| rs7755109 | 6 | 142792085 | 0.302 | 0.072 | 8.50E-05 | G | A | Y | *GPR126* |
| rs7078228 | 10 | 120966733 | 0.016 | 0.272 | 8.56E-05 | C | T | N | *GRK5* |
| rs6929442 | 6 | 142784352 | 0.302 | 0.072 | 8.61E-05 | C | T | N | *GPR126* |
| rs262119 | 6 | 142884486 | 0.303 | 0.072 | 8.68E-05 | C | T | N | *GPR126* |
| rs2673926 | 5 | 135725523 | 0.304 | 0.072 | 8.78E-05 | A | G | Y | *TRPC7* |
| rs7307889 | 12 | 5851614 | 0.028 | 0.206 | 8.80E-05 | T | C | N | *TMEM16B* |
| rs11063895 | 12 | 5851268 | 0.028 | 0.206 | 8.82E-05 | T | C | Y | *TMEM16B* |
| rs2140825 | 3 | 171473104 | 0.375 | -0.069 | 8.84E-05 | C | T | Y | *PRKCI* |
| rs6478121 | 9 | 116756341 | 0.433 | -0.067 | 8.90E-05 | C | G | Y | *TNFSF8* |
| rs6498385 | 16 | 13167388 | 0.442 | 0.067 | 9.03E-05 | A | G | N | *FLJ11151* |
| rs2290720 | 12 | 100211174 | 0.421 | 0.067 | 9.07E-05 | T | C | N | *UTP20* |
| rs824313 | 12 | 100195708 | 0.420 | 0.067 | 9.09E-05 | C | T | N | *UTP20* |
| rs9459625 | 6 | 166591327 | 0.370 | 0.071 | 9.19E-05 | G | C | N | *PRR18* |
| rs2294764 | 6 | 142779197 | 0.302 | 0.072 | 9.30E-05 | A | G | N | *GPR126* |
| rs6489665 | 12 | 5838279 | 0.028 | 0.210 | 9.36E-05 | T | C | N | *TMEM16B* |
| rs638052 | 18 | 68379883 | 0.086 | -0.119 | 9.37E-05 | C | T | N | *CBLN2* |
| rs2798819 | 14 | 94492794 | 0.293 | 0.072 | 9.40E-05 | G | T | Y | *DICER1* |
| rs7312071 | 12 | 100223113 | 0.421 | 0.067 | 9.41E-05 | G | A | N | *UTP20* |
| rs2497782 | 10 | 18359047 | 0.319 | 0.072 | 9.44E-05 | G | A | N | *SLC39A12* |
| rs7753012 | 6 | 142787576 | 0.315 | 0.072 | 9.50E-05 | G | T | N | *GPR126* |
| rs17098556 | 10 | 120968173 | 0.016 | 0.265 | 9.50E-05 | G | A | Y | *GRK5* |
| rs6986750 | 8 | 61779069 | 0.025 | -0.209 | 9.52E-05 | G | C | N | *CHD7* |
| rs11609516 | 12 | 100279051 | 0.239 | 0.078 | 9.54E-05 | G | C | Y | *UTP20* |
| rs17158566 | 7 | 29998209 | 0.019 | -0.242 | 9.56E-05 | G | C | Y | *SCRN1* |
| rs6795631 | 3 | 287177 | 0.282 | -0.074 | 9.58E-05 | A | G | Y | *CHL1* |
| rs10507130 | 12 | 100278085 | 0.239 | 0.078 | 9.64E-05 | A | G | N | *UTP20* |
| rs2460 | 15 | 50860376 | 0.241 | -0.078 | 9.65E-05 | A | G | N | *ONECUT1* |
| rs10817691 | 9 | 116760083 | 0.548 | 0.068 | 9.83E-05 | A | C | N | *TNFSF8* |
| rs7971079 | 12 | 93967620 | 0.252 | -0.076 | 9.83E-05 | G | A | N | *NR2C1* |
| rs11107877 | 12 | 93970892 | 0.252 | -0.076 | 9.83E-05 | C | T | N | *NR2C1* |
| rs894220 | 1 | 29955601 | 0.401 | -0.068 | 1.01E-04 | A | G | N | *PTPRU* |
| rs894219 | 1 | 29956046 | 0.401 | -0.067 | 1.01E-04 | A | G | N | *PTPRU* |
| rs11596974 | 10 | 18460390 | 0.225 | -0.081 | 1.01E-04 | A | G | N | *CACNB2* |
| rs1880589 | 3 | 171525496 | 0.395 | -0.070 | 1.02E-04 | T | G | N | *PRKCI* |
| rs894217 | 1 | 29956326 | 0.401 | -0.067 | 1.02E-04 | T | C | N | *PTPRU* |
| rs2673925 | 5 | 135733573 | 0.304 | 0.071 | 1.02E-04 | A | G | Y | *TRPC7* |
| rs4259710 | 1 | 29957013 | 0.401 | -0.067 | 1.02E-04 | G | A | Y | *PTPRU* |
| rs6028308 | 20 | 37230035 | 0.262 | 0.078 | 1.03E-04 | A | G | N | *DHX35* |
| rs9917322 | 2 | 20535781 | 0.464 | 0.069 | 1.03E-04 | A | G | N | *RHOB* |
| rs4666402 | 2 | 20536729 | 0.464 | 0.069 | 1.03E-04 | G | A | N | *RHOB* |
| rs10914228 | 1 | 29963181 | 0.423 | -0.069 | 1.03E-04 | T | G | N | *PTPRU* |
| rs4666308 | 2 | 20536254 | 0.464 | 0.069 | 1.03E-04 | G | A | N | *RHOB* |
| rs10205221 | 2 | 20537725 | 0.464 | 0.069 | 1.03E-04 | A | C | N | *RHOB* |
| rs7313312 | 12 | 100279814 | 0.239 | 0.078 | 1.03E-04 | C | T | Y | *UTP20* |
| rs9636161 | 19 | 61592298 | 0.449 | -0.067 | 1.04E-04 | T | C | Y | *ZNF582* |
| rs1265917 | 9 | 116762015 | 0.445 | -0.067 | 1.04E-04 | A | G | N | *TNFSF8* |
| rs12988451 | 2 | 20533218 | 0.463 | 0.068 | 1.04E-04 | A | G | N | *RHOB* |
| rs1997061 | 3 | 171515371 | 0.393 | -0.069 | 1.05E-04 | T | C | N | *PRKCI* |
| rs6460531 | 7 | 68937354 | 0.102 | 0.109 | 1.06E-04 | G | C | N | *AUTS2* |
| rs2658842 | 11 | 131058349 | 0.257 | 0.075 | 1.08E-04 | C | T | N | *HNT* |
| rs1880588 | 3 | 171525505 | 0.393 | -0.069 | 1.08E-04 | G | C | N | *PRKCI* |
| rs2739265 | 11 | 131055105 | 0.257 | 0.075 | 1.09E-04 | T | C | N | *HNT* |
| rs17458533 | 7 | 68950443 | 0.101 | 0.109 | 1.09E-04 | G | A | N | *AUTS2* |
| rs593789 | 18 | 68376865 | 0.081 | -0.120 | 1.09E-04 | T | A | N | *CBLN2* |
| rs2739264 | 11 | 131054974 | 0.257 | 0.075 | 1.09E-04 | T | C | N | *HNT* |
| rs9310278 | 3 | 73788941 | 0.229 | -0.078 | 1.10E-04 | A | G | N | *PDZRN3* |
| rs17457848 | 7 | 68921413 | 0.102 | 0.109 | 1.10E-04 | T | C | N | *AUTS2* |
| rs9310276 | 3 | 73786831 | 0.230 | -0.078 | 1.11E-04 | A | G | Y | *PDZRN3* |
| rs13188105 | 5 | 5969224 | 0.166 | 0.090 | 1.11E-04 | T | C | N | *FLJ33360* |
| rs2658836 | 11 | 131054912 | 0.257 | 0.075 | 1.11E-04 | C | T | N | *HNT* |
| rs17158618 | 7 | 30089670 | 0.019 | -0.240 | 1.11E-04 | G | A | N | *PLEKHA8* |
| rs12807796 | 11 | 86155408 | 0.146 | -0.095 | 1.11E-04 | C | T | N | *PRSS23* |
| rs995043 | 7 | 30078087 | 0.019 | -0.240 | 1.11E-04 | A | G | Y | *PLEKHA8* |
| rs17158623 | 7 | 30097689 | 0.019 | -0.240 | 1.11E-04 | G | A | Y | *PLEKHA8* |
| rs11234798 | 11 | 86155979 | 0.146 | -0.095 | 1.12E-04 | C | G | N | *PRSS23* |
| rs2135533 | 11 | 131054745 | 0.257 | 0.075 | 1.12E-04 | T | C | Y | *HNT* |
| rs17459271 | 7 | 69008880 | 0.101 | 0.109 | 1.12E-04 | A | G | N | *AUTS2* |
| rs7047645 | 9 | 72355453 | 0.486 | 0.066 | 1.12E-04 | A | G | N | *TRPM3* |
| rs2658833 | 11 | 131053736 | 0.258 | 0.075 | 1.12E-04 | G | A | Y | *HNT* |
| rs2463407 | 11 | 131053582 | 0.257 | 0.075 | 1.12E-04 | T | C | N | *HNT* |
| rs1323954 | 13 | 34333049 | 0.063 | -0.140 | 1.12E-04 | C | A | Y | *NBEA* |
| rs1542892 | 5 | 5971529 | 0.166 | 0.090 | 1.13E-04 | C | G | N | *FLJ33360* |
| rs7790458 | 7 | 68990553 | 0.101 | 0.109 | 1.13E-04 | A | G | N | *AUTS2* |
| rs1788002 | 18 | 53387425 | 0.462 | 0.065 | 1.13E-04 | T | C | Y | *FECH* |
| rs6805318 | 3 | 73784679 | 0.230 | -0.078 | 1.13E-04 | A | G | N | *PDZRN3* |
| rs17361399 | 7 | 68965752 | 0.101 | 0.109 | 1.13E-04 | T | C | Y | *AUTS2* |
| rs17361448 | 7 | 68967414 | 0.101 | 0.109 | 1.13E-04 | A | T | N | *AUTS2* |
| rs2658828 | 11 | 131053097 | 0.257 | 0.075 | 1.13E-04 | A | G | N | *HNT* |
| rs2658830 | 11 | 131053361 | 0.257 | 0.075 | 1.13E-04 | G | C | N | *HNT* |
| rs6781766 | 3 | 73784634 | 0.230 | -0.078 | 1.13E-04 | T | C | N | *PDZRN3* |
| rs2658829 | 11 | 131053274 | 0.257 | 0.075 | 1.13E-04 | G | A | N | *HNT* |
| rs7025694 | 9 | 72355083 | 0.487 | 0.066 | 1.14E-04 | C | T | N | *TRPM3* |
| rs2695812 | 11 | 131053050 | 0.257 | 0.075 | 1.14E-04 | C | T | N | *HNT* |
| rs17459722 | 7 | 69040697 | 0.101 | 0.109 | 1.14E-04 | C | T | N | *AUTS2* |
| rs17362481 | 7 | 69045030 | 0.101 | 0.109 | 1.14E-04 | T | G | Y | *AUTS2* |
| rs17459369 | 7 | 69021701 | 0.101 | 0.108 | 1.14E-04 | G | A | Y | *AUTS2* |
| rs2658827 | 11 | 131053026 | 0.257 | 0.075 | 1.14E-04 | G | T | N | *HNT* |
| rs1889915 | 9 | 72354532 | 0.487 | 0.066 | 1.14E-04 | C | T | Y | *TRPM3* |
| rs634985 | 18 | 68392317 | 0.081 | -0.120 | 1.14E-04 | T | C | N | *CBLN2* |
| rs2658826 | 11 | 131053009 | 0.257 | 0.076 | 1.14E-04 | C | T | N | *HNT* |
| rs1511520 | 3 | 73784368 | 0.230 | -0.078 | 1.14E-04 | C | T | N | *PDZRN3* |
| rs10780946 | 9 | 72353420 | 0.487 | 0.066 | 1.15E-04 | C | T | N | *TRPM3* |
| rs262115 | 6 | 142859100 | 0.322 | 0.071 | 1.16E-04 | C | T | N | *GPR126* |
| rs9864199 | 3 | 115593868 | 0.436 | -0.066 | 1.17E-04 | A | G | N | *ZBTB20* |
| rs7973083 | 12 | 100248212 | 0.240 | 0.077 | 1.17E-04 | A | T | N | *UTP20* |
| rs2693815 | 2 | 6114642 | 0.300 | 0.071 | 1.17E-04 | G | A | N | *SOX11* |
| rs225210 | 17 | 27919165 | 0.446 | -0.068 | 1.17E-04 | G | A | N | *MYO1D* |
| rs6494904 | 15 | 69396576 | 0.266 | -0.074 | 1.17E-04 | G | A | Y | *LOC645296* |
| rs4325427 | 13 | 40114534 | 0.291 | 0.072 | 1.18E-04 | T | C | Y | *FOXO1* |
| rs6129210 | 20 | 37250882 | 0.242 | 0.077 | 1.18E-04 | G | A | N | *DHX35* |
| rs155259 | 6 | 142888258 | 0.303 | 0.071 | 1.18E-04 | A | G | N | *GPR126* |
| rs9673 | 9 | 96901258 | 0.030 | -0.196 | 1.18E-04 | G | A | Y | *FANCC* |
| rs2649696 | 5 | 135734750 | 0.304 | 0.070 | 1.18E-04 | C | T | Y | *TRPC7* |
| rs932017 | 3 | 73817337 | 0.350 | -0.069 | 1.19E-04 | G | A | N | *PDZRN3* |
| rs9088 | 7 | 150132449 | 0.334 | -0.069 | 1.19E-04 | A | G | N | *TMEM176A* |
| rs2609114 | 2 | 6113619 | 0.300 | 0.071 | 1.19E-04 | C | A | N | *SOX11* |
| rs7763064 | 6 | 142838982 | 0.309 | 0.070 | 1.20E-04 | A | G | Y | *GPR126* |
| rs741067 | 7 | 150131388 | 0.334 | -0.070 | 1.20E-04 | A | G | N | *TMEM176A* |
| rs6517532 | 21 | 39564059 | 0.057 | -0.141 | 1.20E-04 | C | T | Y | *BRWD1* |
| rs4744602 | 9 | 72348947 | 0.487 | 0.066 | 1.21E-04 | C | A | N | *TRPM3* |
| rs3957585 | 3 | 115594746 | 0.435 | -0.066 | 1.22E-04 | A | G | Y | *ZBTB20* |
| rs4744601 | 9 | 72348926 | 0.487 | 0.066 | 1.22E-04 | A | T | N | *TRPM3* |
| rs1511532 | 3 | 73818364 | 0.350 | -0.069 | 1.22E-04 | T | C | N | *PDZRN3* |
| rs1949914 | 2 | 6113298 | 0.301 | 0.071 | 1.22E-04 | C | T | Y | *SOX11* |
| rs262129 | 6 | 142894837 | 0.303 | 0.071 | 1.22E-04 | G | A | N | *GPR126* |
| rs1511533 | 3 | 73818420 | 0.350 | -0.069 | 1.22E-04 | G | A | N | *PDZRN3* |
| rs1475080 | 6 | 17145237 | 0.447 | 0.066 | 1.23E-04 | T | A | Y | *RBM24* |
| rs2609115 | 2 | 6112182 | 0.300 | 0.071 | 1.23E-04 | G | A | N | *SOX11* |
| rs714885 | 7 | 150130720 | 0.334 | -0.070 | 1.23E-04 | T | C | N | *TMEM176B* |
| rs12657635 | 5 | 43473667 | 0.020 | 0.235 | 1.23E-04 | A | G | N | *C5orf28* |
| rs13155888 | 5 | 43481775 | 0.020 | 0.236 | 1.25E-04 | A | T | N | *C5orf28* |
| rs17641623 | 5 | 90937093 | 0.526 | 0.065 | 1.26E-04 | A | G | Y | *ARRDC3* |
| rs8083020 | 18 | 29502420 | 0.065 | 0.132 | 1.26E-04 | A | T | N | *ASXL3* |
| rs11593214 | 10 | 12338601 | 0.587 | 0.066 | 1.27E-04 | C | G | Y | *CDC123* |
| rs6787034 | 3 | 73847232 | 0.459 | -0.065 | 1.28E-04 | A | G | N | *PDZRN3* |
| rs4834988 | 4 | 145698227 | 0.455 | -0.065 | 1.28E-04 | C | A | N | *HHIP* |
| rs2798821 | 14 | 94492244 | 0.287 | 0.073 | 1.29E-04 | T | C | N | *DICER1* |
| rs10502624 | 18 | 29501059 | 0.065 | 0.132 | 1.29E-04 | T | C | Y | *ASXL3* |
| rs7703346 | 5 | 90945330 | 0.526 | 0.065 | 1.30E-04 | A | T | N | *ARRDC3* |
| rs9620595 | 22 | 24964750 | 0.036 | 0.178 | 1.31E-04 | T | C | N | *SEZ6L* |
| rs4799351 | 18 | 29459187 | 0.065 | 0.133 | 1.31E-04 | G | C | Y | *ASXL3* |
| rs3024471 | 6 | 6264534 | 0.027 | -0.198 | 1.31E-04 | G | A | Y | *F13A1* |
| rs17665911 | 18 | 29465335 | 0.065 | 0.133 | 1.31E-04 | G | A | N | *ASXL3* |
| rs12510044 | 4 | 145704088 | 0.455 | -0.065 | 1.31E-04 | C | G | N | *HHIP* |
| rs17158629 | 7 | 30125304 | 0.019 | -0.236 | 1.32E-04 | A | G | Y | *C7orf41* |
| rs2078482 | 6 | 22105905 | 0.531 | 0.064 | 1.32E-04 | G | A | Y | *PRL* |
| rs7645388 | 3 | 73848121 | 0.459 | -0.065 | 1.32E-04 | G | A | N | *PDZRN3* |
| rs263179 | 6 | 142905581 | 0.303 | 0.070 | 1.33E-04 | C | T | N | *GPR126* |
| rs2040201 | 18 | 29456104 | 0.065 | 0.133 | 1.34E-04 | A | G | N | *ASXL3* |
| rs17665756 | 18 | 29455503 | 0.065 | 0.133 | 1.34E-04 | C | T | N | *ASXL3* |
| rs9956208 | 18 | 29453829 | 0.065 | 0.133 | 1.35E-04 | G | C | N | *ASXL3* |
| rs2271804 | 10 | 12292223 | 0.554 | 0.067 | 1.35E-04 | A | G | N | *NUDT5* |
| rs1959056 | 14 | 56045506 | 0.349 | 0.068 | 1.35E-04 | A | G | N | *C14orf101* |
| rs9959496 | 18 | 29454798 | 0.065 | 0.133 | 1.36E-04 | G | C | N | *ASXL3* |
| rs4799703 | 18 | 29445545 | 0.065 | 0.133 | 1.37E-04 | C | T | N | *ASXL3* |
| rs11855326 | 15 | 69397889 | 0.264 | -0.073 | 1.37E-04 | A | G | Y | *LOC645296* |
| rs747742 | 17 | 69741087 | 0.222 | -0.078 | 1.38E-04 | A | G | N | *TTYH2* |
| rs1791481 | 18 | 33259093 | 0.303 | -0.073 | 1.38E-04 | A | T | N | *BRUNOL4* |
| rs811165 | 14 | 53677512 | 0.156 | -0.089 | 1.38E-04 | G | A | N | *BMP4* |
| rs2382837 | 17 | 69748982 | 0.222 | -0.078 | 1.38E-04 | T | C | Y | *TTYH2* |
| rs2550357 | 16 | 56771068 | 0.148 | -0.091 | 1.39E-04 | C | T | N | *CSNK2A2* |
| rs16968931 | 18 | 33258803 | 0.303 | -0.073 | 1.39E-04 | A | G | N | *BRUNOL4* |
| rs952708 | 3 | 73849883 | 0.459 | -0.065 | 1.39E-04 | C | T | N | *PDZRN3* |
| rs3817928 | 6 | 142792209 | 0.216 | 0.078 | 1.40E-04 | G | A | N | *GPR126* |
| rs1511531 | 3 | 73850194 | 0.459 | -0.065 | 1.40E-04 | C | T | N | *PDZRN3* |
| rs12657724 | 5 | 43465132 | 0.021 | 0.230 | 1.41E-04 | C | T | N | *C5orf28* |
| rs7194812 | 16 | 79870467 | 0.262 | -0.073 | 1.41E-04 | G | A | N | *BCMO1* |
| rs7192773 | 16 | 79870507 | 0.262 | -0.073 | 1.42E-04 | C | G | Y | *BCMO1* |
| rs10906097 | 10 | 12283827 | 0.554 | 0.067 | 1.42E-04 | G | T | N | *NUDT5* |
| rs1878798 | 10 | 12283489 | 0.554 | 0.067 | 1.42E-04 | G | C | N | *NUDT5* |
| rs7188650 | 16 | 79871882 | 0.262 | -0.073 | 1.42E-04 | C | T | N | *BCMO1* |
| rs726147 | 18 | 29442138 | 0.065 | 0.135 | 1.43E-04 | A | G | N | *ASXL3* |
| rs2673913 | 5 | 135749346 | 0.304 | 0.070 | 1.43E-04 | C | G | Y | *TRPC7* |
| rs17384972 | 22 | 26402029 | 0.041 | 0.163 | 1.44E-04 | T | C | Y | *MN1* |
| rs6570511 | 6 | 142799061 | 0.215 | 0.078 | 1.44E-04 | A | G | N | *GPR126* |
| rs1323956 | 13 | 34333190 | 0.062 | -0.138 | 1.44E-04 | T | C | N | *NBEA* |
| rs6890108 | 5 | 43462224 | 0.021 | 0.230 | 1.44E-04 | T | C | N | *CCL28* |
| rs4369918 | 2 | 134205469 | 0.168 | 0.086 | 1.46E-04 | A | C | N | *NAP5* |
| rs2550355 | 16 | 56766333 | 0.148 | -0.091 | 1.46E-04 | T | C | N | *CSNK2A2* |
| rs6889653 | 5 | 43462066 | 0.021 | 0.229 | 1.46E-04 | G | A | N | *CCL28* |
| rs1042717 | 5 | 148186839 | 0.209 | 0.079 | 1.48E-04 | A | G | N | *ADRB2* |
| rs2789376 | 14 | 94490218 | 0.295 | 0.070 | 1.48E-04 | A | G | N | *DICER1* |
| rs11959615 | 5 | 148181509 | 0.404 | -0.068 | 1.49E-04 | T | A | N | *ADRB2* |
| rs2488132 | 10 | 18369824 | 0.434 | 0.065 | 1.49E-04 | C | T | Y | *SLC39A12* |
| rs12343979 | 9 | 79364768 | 0.067 | 0.131 | 1.49E-04 | T | A | N | *GNA14* |
| rs611802 | 6 | 142908080 | 0.303 | 0.070 | 1.49E-04 | C | A | N | *GPR126* |
| rs9599756 | 13 | 34333744 | 0.062 | -0.138 | 1.49E-04 | C | T | N | *NBEA* |
| rs731890 | 18 | 33256116 | 0.304 | -0.072 | 1.49E-04 | G | A | N | *BRUNOL4* |
| rs10108922 | 8 | 72886643 | 0.248 | -0.075 | 1.49E-04 | C | T | N | *MSC* |
| rs11817797 | 10 | 12338450 | 0.587 | 0.066 | 1.49E-04 | T | C | N | *CDC123* |
| rs2292028 | 16 | 56758474 | 0.148 | -0.091 | 1.50E-04 | T | C | N | *CSNK2A2* |
| rs2242445 | 16 | 56756704 | 0.148 | -0.090 | 1.51E-04 | T | G | Y | *CSNK2A2* |
| rs225209 | 17 | 27918999 | 0.468 | -0.064 | 1.51E-04 | A | G | Y | *MYO1D* |
| rs4747968 | 10 | 12289803 | 0.610 | 0.068 | 1.51E-04 | T | C | N | *NUDT5* |
| rs12775830 | 10 | 12289451 | 0.611 | 0.068 | 1.52E-04 | G | C | N | *NUDT5* |
| rs1941945 | 18 | 33254085 | 0.304 | -0.072 | 1.52E-04 | A | G | N | *BRUNOL4* |
| rs11819728 | 10 | 12338200 | 0.587 | 0.066 | 1.53E-04 | G | A | N | *CDC123* |
| rs1505771 | 4 | 145458435 | 0.306 | -0.070 | 1.53E-04 | T | C | N | *GYPA* |
| rs4256191 | 4 | 145459461 | 0.306 | -0.070 | 1.53E-04 | C | A | N | *GYPA* |
| rs7377575 | 4 | 145474135 | 0.306 | -0.070 | 1.53E-04 | T | C | N | *GYPA* |
| rs4321584 | 4 | 145459602 | 0.307 | -0.070 | 1.54E-04 | G | A | Y | *GYPA* |
| rs7655235 | 4 | 145469840 | 0.306 | -0.070 | 1.54E-04 | A | G | Y | *GYPA* |
| rs4965599 | 15 | 98577407 | 0.348 | -0.068 | 1.55E-04 | C | G | Y | *ADAMTS17* |
| rs12413792 | 10 | 12289295 | 0.611 | 0.068 | 1.55E-04 | C | T | N | *NUDT5* |
| rs11875702 | 18 | 33249230 | 0.304 | -0.072 | 1.56E-04 | C | T | N | *BRUNOL4* |
| rs1935506 | 10 | 18356946 | 0.324 | 0.069 | 1.56E-04 | G | A | N | *SLC39A12* |
| rs17158631 | 7 | 30127948 | 0.019 | -0.234 | 1.56E-04 | T | C | N | *C7orf41* |
| rs2082395 | 5 | 148180793 | 0.427 | -0.064 | 1.57E-04 | A | G | Y | *ADRB2* |
| rs10492726 | 13 | 34334375 | 0.062 | -0.138 | 1.59E-04 | G | T | N | *NBEA* |
| rs2082382 | 5 | 148180746 | 0.427 | -0.064 | 1.59E-04 | G | A | N | *ADRB2* |
| rs263178 | 6 | 142906799 | 0.303 | 0.069 | 1.59E-04 | T | C | Y | *GPR126* |
| rs10269275 | 7 | 13640704 | 0.412 | -0.065 | 1.59E-04 | G | A | Y | *ETV1* |
| rs2893225 | 1 | 93141084 | 0.387 | 0.069 | 1.59E-04 | G | C | N | *FAM69A* |
| rs11707796 | 3 | 291417 | 0.306 | -0.070 | 1.59E-04 | C | T | Y | *CHL1* |
| rs1570909 | 6 | 9448764 | 0.096 | -0.112 | 1.59E-04 | C | T | N | *OFCC1* |
| rs6082456 | 20 | 21500714 | 0.404 | -0.066 | 1.60E-04 | G | A | Y | *NKX2-2* |
| rs17084708 | 18 | 67384837 | 0.302 | -0.070 | 1.61E-04 | C | T | Y | *CBLN2* |
| rs6764363 | 3 | 287349 | 0.377 | -0.067 | 1.62E-04 | C | T | N | *CHL1* |
| rs4281830 | 19 | 61625097 | 0.499 | 0.063 | 1.62E-04 | G | A | Y | *ZNF583* |
| rs11660297 | 18 | 67381007 | 0.302 | -0.070 | 1.62E-04 | G | T | N | *CBLN2* |
| rs12773913 | 10 | 12289230 | 0.611 | 0.067 | 1.64E-04 | T | G | N | *NUDT5* |
| rs917651 | 19 | 61625676 | 0.499 | 0.063 | 1.64E-04 | C | G | Y | *ZNF583* |
| rs917652 | 19 | 61625923 | 0.499 | 0.063 | 1.64E-04 | A | G | N | *ZNF583* |
| rs12448461 | 16 | 13158917 | 0.444 | 0.066 | 1.65E-04 | G | A | N | *FLJ11151* |
| rs4965593 | 15 | 98575908 | 0.330 | -0.071 | 1.65E-04 | C | G | N | *ADAMTS17* |
| rs3810340 | 19 | 61627339 | 0.499 | 0.063 | 1.65E-04 | A | G | N | *ZNF583* |
| rs9599757 | 13 | 34335110 | 0.062 | -0.137 | 1.65E-04 | G | A | N | *NBEA* |
| rs8104319 | 19 | 61631383 | 0.499 | 0.063 | 1.65E-04 | G | A | N | *ZNF583* |
| rs11909465 | 21 | 24911738 | 0.010 | -0.316 | 1.66E-04 | A | G | N | *MRPL39* |
| rs11151678 | 18 | 67377933 | 0.302 | -0.070 | 1.66E-04 | G | C | N | *CBLN2* |
| rs11151679 | 18 | 67378014 | 0.302 | -0.070 | 1.66E-04 | G | A | N | *CBLN2* |
| rs9599764 | 13 | 34338882 | 0.136 | -0.095 | 1.66E-04 | C | T | N | *NBEA* |
| rs1040526 | 6 | 142777509 | 0.300 | 0.070 | 1.67E-04 | G | A | N | *GPR126* |
| rs7264927 | 20 | 57247948 | 0.123 | 0.101 | 1.67E-04 | T | G | N | *C20orf174* |
| rs9493341 | 6 | 132872637 | 0.375 | 0.066 | 1.67E-04 | G | A | Y | *STX7* |
| rs11073348 | 15 | 36808652 | 0.338 | -0.071 | 1.67E-04 | A | G | N | *FLJ35695* |
| rs17000152 | 21 | 24911290 | 0.010 | -0.315 | 1.67E-04 | T | C | Y | *MRPL39* |
| rs11253188 | 10 | 5497613 | 0.284 | -0.072 | 1.68E-04 | C | T | N | *NET1* |
| rs2408980 | 21 | 24910194 | 0.010 | -0.315 | 1.68E-04 | T | C | N | *MRPL39* |
| rs11084449 | 19 | 61633541 | 0.499 | 0.063 | 1.68E-04 | G | A | N | *ZNF583* |
| rs12460342 | 19 | 61633831 | 0.499 | 0.063 | 1.68E-04 | C | G | N | *ZNF583* |
| rs11100850 | 4 | 145457875 | 0.303 | -0.072 | 1.69E-04 | T | A | N | *GYPA* |
| rs12973407 | 19 | 61619933 | 0.500 | 0.063 | 1.70E-04 | G | A | N | *ZNF583* |
| rs11084450 | 19 | 61634034 | 0.499 | 0.063 | 1.71E-04 | C | T | N | *ZNF583* |
| rs728232 | 19 | 61636200 | 0.499 | 0.063 | 1.72E-04 | T | C | Y | *ZNF667* |
| rs962554 | 6 | 142775897 | 0.300 | 0.069 | 1.72E-04 | C | T | N | *GPR126* |
| rs13160431 | 5 | 43458237 | 0.021 | 0.224 | 1.75E-04 | G | T | N | *CCL28* |
| rs2239547 | 3 | 52830269 | 0.268 | 0.074 | 1.76E-04 | C | T | N | *ITIH4* |
| rs6041822 | 20 | 12985152 | 0.169 | -0.084 | 1.77E-04 | C | G | N | *SPTLC3* |
| rs3857420 | 5 | 148193275 | 0.182 | 0.082 | 1.77E-04 | A | G | Y | *ADRB2* |
| rs4438885 | 5 | 43456847 | 0.021 | 0.224 | 1.77E-04 | C | A | N | *CCL28* |
| rs7320901 | 13 | 34346087 | 0.135 | -0.095 | 1.79E-04 | T | C | N | *NBEA* |
| rs4478891 | 10 | 12307660 | 0.573 | 0.065 | 1.79E-04 | G | A | N | *CDC123* |
| rs6562657 | 13 | 34336443 | 0.062 | -0.137 | 1.79E-04 | C | T | N | *NBEA* |
| rs9350404 | 6 | 22102723 | 0.477 | 0.064 | 1.79E-04 | A | G | N | *PRL* |
| rs7774095 | 6 | 142712555 | 0.307 | 0.071 | 1.80E-04 | A | C | N | *GPR126* |
| rs1023933 | 4 | 34826055 | 0.421 | -0.064 | 1.80E-04 | A | C | Y | *CENTD1* |
| rs1437660 | 2 | 164461232 | 0.267 | 0.072 | 1.80E-04 | G | A | Y | *FIGN* |
| rs1897293 | 20 | 12971715 | 0.355 | -0.066 | 1.83E-04 | C | T | Y | *SPTLC3* |
| rs6041820 | 20 | 12984607 | 0.169 | -0.084 | 1.83E-04 | T | G | N | *SPTLC3* |
| rs11168066 | 5 | 148181448 | 0.427 | -0.063 | 1.83E-04 | C | A | Y | *ADRB2* |
| rs6570508 | 6 | 142755535 | 0.314 | 0.069 | 1.84E-04 | A | G | N | *GPR126* |
| rs926244 | 1 | 2996143 | 0.322 | -0.069 | 1.84E-04 | G | A | N | *PRDM16* |
| rs824311 | 12 | 100195249 | 0.443 | 0.064 | 1.84E-04 | G | A | N | *UTP20* |
| rs6711676 | 2 | 164462857 | 0.267 | 0.072 | 1.85E-04 | A | G | N | *FIGN* |
| rs6937121 | 6 | 142748826 | 0.300 | 0.069 | 1.85E-04 | G | T | Y | *GPR126* |
| rs211405 | 10 | 32431717 | 0.352 | 0.066 | 1.85E-04 | G | A | N | *KIF5B* |
| rs651217 | 13 | 52300292 | 0.388 | -0.066 | 1.86E-04 | C | A | N | *PCDH8* |
| rs12441227 | 15 | 69483940 | 0.161 | -0.086 | 1.86E-04 | G | A | Y | *LOC645296* |
| rs2981881 | 1 | 2994217 | 0.322 | -0.068 | 1.86E-04 | C | T | Y | *PRDM16* |
| rs6041818 | 20 | 12984336 | 0.169 | -0.084 | 1.87E-04 | A | G | N | *SPTLC3* |
| rs11234768 | 11 | 86126487 | 0.162 | -0.086 | 1.87E-04 | C | T | N | *PRSS23* |
| rs12195563 | 6 | 150897557 | 0.304 | 0.069 | 1.87E-04 | G | A | Y | *PLEKHG1* |
| rs11234767 | 11 | 86126352 | 0.162 | -0.086 | 1.87E-04 | C | A | N | *PRSS23* |
| rs16920077 | 8 | 55409655 | 0.255 | -0.072 | 1.88E-04 | T | C | N | *SOX17* |
| rs13208542 | 6 | 150898463 | 0.304 | 0.069 | 1.88E-04 | C | G | Y | *PLEKHG1* |
| rs211408 | 10 | 32433148 | 0.352 | 0.066 | 1.88E-04 | A | C | N | *KIF5B* |
| rs12195029 | 6 | 150900625 | 0.304 | 0.069 | 1.88E-04 | C | T | Y | *PLEKHG1* |
| rs13182748 | 5 | 5968186 | 0.126 | 0.096 | 1.89E-04 | A | C | N | *FLJ33360* |
| rs10898531 | 11 | 86125740 | 0.162 | -0.086 | 1.89E-04 | A | G | Y | *PRSS23* |
| rs6109674 | 20 | 12984227 | 0.169 | -0.084 | 1.89E-04 | A | C | N | *SPTLC3* |
| rs925431 | 3 | 73852454 | 0.460 | -0.063 | 1.90E-04 | G | A | Y | *PDZRN3* |
| rs4429172 | 13 | 40087143 | 0.311 | 0.069 | 1.90E-04 | A | C | N | *FOXO1* |
| rs12631241 | 3 | 73845995 | 0.225 | -0.076 | 1.91E-04 | C | A | N | *PDZRN3* |
| rs6570509 | 6 | 142757979 | 0.299 | 0.069 | 1.91E-04 | T | G | N | *GPR126* |
| rs7035746 | 9 | 7770854 | 0.026 | 0.206 | 1.92E-04 | T | G | N | *C9orf123* |
| rs2985381 | 11 | 34879792 | 0.341 | 0.068 | 1.92E-04 | G | C | N | *APIP* |
| rs7314897 | 12 | 115909537 | 0.011 | -0.295 | 1.93E-04 | G | T | N | *FBXW8* |
| rs970543 | 3 | 73842112 | 0.225 | -0.076 | 1.93E-04 | C | T | N | *PDZRN3* |
| rs7133609 | 12 | 115923799 | 0.011 | -0.295 | 1.93E-04 | T | A | N | *FBXW8* |
| rs16947233 | 12 | 115944460 | 0.011 | -0.295 | 1.93E-04 | C | T | Y | *FBXW8* |
| rs10164803 | 2 | 164471146 | 0.267 | 0.071 | 1.93E-04 | T | C | Y | *FIGN* |
| rs16947209 | 12 | 115929565 | 0.011 | -0.295 | 1.93E-04 | A | C | N | *FBXW8* |
| rs6975213 | 7 | 69083754 | 0.109 | 0.104 | 1.93E-04 | G | T | N | *AUTS2* |
| rs9299670 | 10 | 32454636 | 0.362 | 0.066 | 1.93E-04 | T | C | N | *KIF5B* |
| rs1837103 | 2 | 164472128 | 0.267 | 0.071 | 1.93E-04 | T | C | N | *FIGN* |
| rs1439568 | 5 | 124573631 | 0.404 | -0.064 | 1.95E-04 | C | T | Y | *ZNF608* |
| rs6041817 | 20 | 12982926 | 0.169 | -0.084 | 1.97E-04 | G | A | N | *SPTLC3* |
| rs1755066 | 10 | 32349166 | 0.491 | -0.063 | 1.98E-04 | G | C | N | *KIF5B* |
| rs10998726 | 10 | 70769228 | 0.061 | 0.134 | 1.98E-04 | T | G | N | *HK1* |
| rs3743558 | 16 | 56589987 | 0.227 | -0.079 | 1.98E-04 | T | C | N | *ZNF319* |
| rs10998725 | 10 | 70769201 | 0.061 | 0.134 | 1.98E-04 | A | G | N | *HK1* |
| rs1775715 | 10 | 32349011 | 0.491 | -0.063 | 1.99E-04 | G | A | N | *KIF5B* |
| rs6033601 | 20 | 12982865 | 0.169 | -0.083 | 1.99E-04 | G | A | N | *SPTLC3* |
| rs7172574 | 15 | 69488506 | 0.163 | -0.088 | 2.00E-04 | T | C | N | *LOC645296* |
| rs7172592 | 15 | 69488531 | 0.163 | -0.088 | 2.00E-04 | T | C | N | *LOC645296* |
| rs932426 | 20 | 37307227 | 0.263 | 0.074 | 2.01E-04 | A | G | N | *DHX35* |
| rs7283303 | 21 | 24890938 | 0.010 | -0.320 | 2.01E-04 | A | C | N | *MRPL39* |
| rs1251355 | 10 | 32330152 | 0.490 | -0.063 | 2.01E-04 | G | C | Y | *KIF5B* |
| rs6537297 | 4 | 145721479 | 0.446 | -0.064 | 2.02E-04 | C | T | N | *HHIP* |
| rs6033600 | 20 | 12982811 | 0.169 | -0.083 | 2.02E-04 | A | G | Y | *SPTLC3* |
| rs12664563 | 6 | 142826894 | 0.209 | 0.078 | 2.02E-04 | G | A | N | *GPR126* |
| rs7277223 | 21 | 24889322 | 0.010 | -0.320 | 2.02E-04 | A | C | N | *MRPL39* |
| rs10998731 | 10 | 70772739 | 0.061 | 0.132 | 2.03E-04 | A | G | N | *HK1* |
| rs1431434 | 20 | 12977308 | 0.169 | -0.083 | 2.03E-04 | G | T | N | *SPTLC3* |
| rs1431436 | 20 | 12977187 | 0.169 | -0.083 | 2.03E-04 | G | T | N | *SPTLC3* |
| rs1336380 | 9 | 72353055 | 0.482 | 0.063 | 2.04E-04 | G | T | N | *TRPM3* |
| rs9373347 | 6 | 142821578 | 0.209 | 0.078 | 2.04E-04 | T | C | N | *GPR126* |
| rs1928528 | 6 | 142820802 | 0.209 | 0.078 | 2.04E-04 | G | T | N | *GPR126* |
| rs7776356 | 6 | 142818722 | 0.208 | 0.078 | 2.05E-04 | G | A | N | *GPR126* |
| rs6906468 | 6 | 142811079 | 0.208 | 0.078 | 2.05E-04 | C | T | Y | *GPR126* |
| rs9687386 | 5 | 5974133 | 0.126 | 0.094 | 2.05E-04 | T | C | Y | *FLJ33360* |
| rs6033599 | 20 | 12982735 | 0.169 | -0.083 | 2.05E-04 | G | A | Y | *SPTLC3* |
| rs1995535 | 8 | 55412867 | 0.248 | -0.073 | 2.05E-04 | A | G | N | *SOX17* |
| rs10780948 | 9 | 72372819 | 0.481 | 0.063 | 2.05E-04 | A | T | Y | *TRPM3* |
| rs16920078 | 8 | 55411463 | 0.248 | -0.073 | 2.05E-04 | T | C | N | *SOX17* |
| rs10998744 | 10 | 70793133 | 0.061 | 0.130 | 2.05E-04 | G | A | Y | *HK1* |
| rs10958402 | 8 | 55414136 | 0.248 | -0.073 | 2.06E-04 | C | T | Y | *SOX17* |
| rs6537278 | 4 | 145477389 | 0.298 | -0.070 | 2.06E-04 | A | G | N | *GYPA* |
| rs4031150 | 4 | 145476862 | 0.298 | -0.070 | 2.06E-04 | A | G | N | *GYPA* |
| rs10462810 | 5 | 5975597 | 0.126 | 0.094 | 2.06E-04 | T | G | N | *FLJ33360* |
| rs225215 | 17 | 27921023 | 0.519 | 0.063 | 2.06E-04 | G | A | Y | *MYO1D* |
| rs2294775 | 6 | 142808040 | 0.208 | 0.077 | 2.07E-04 | G | C | Y | *GPR126* |
| rs4896582 | 6 | 142745570 | 0.313 | 0.069 | 2.07E-04 | A | G | N | *GPR126* |
| rs917650 | 19 | 61623484 | 0.475 | 0.063 | 2.07E-04 | C | T | N | *ZNF583* |
| rs1150467 | 5 | 5979188 | 0.126 | 0.094 | 2.07E-04 | T | A | Y | *FLJ33360* |
| rs6705268 | 2 | 164445618 | 0.266 | 0.073 | 2.08E-04 | A | G | N | *FIGN* |
| rs6830386 | 4 | 145454993 | 0.297 | -0.069 | 2.08E-04 | A | G | N | *GYPA* |
| rs3892360 | 13 | 40082078 | 0.311 | 0.068 | 2.08E-04 | G | C | Y | *FOXO1* |
| rs10823349 | 10 | 70779704 | 0.061 | 0.130 | 2.09E-04 | A | C | Y | *HK1* |
| rs12901896 | 15 | 69489319 | 0.163 | -0.089 | 2.09E-04 | G | A | N | *LOC645296* |
| rs6063438 | 20 | 47874575 | 0.203 | 0.078 | 2.09E-04 | T | C | Y | *SLC9A8* |
| rs10497248 | 2 | 164460708 | 0.267 | 0.071 | 2.09E-04 | T | C | Y | *FIGN* |
| rs6909857 | 6 | 142742851 | 0.299 | 0.069 | 2.09E-04 | G | A | N | *GPR126* |
| rs7986407 | 13 | 40077798 | 0.311 | 0.068 | 2.10E-04 | G | A | N | *FOXO1* |
| rs4777384 | 15 | 69489673 | 0.163 | -0.089 | 2.10E-04 | C | T | N | *LOC645296* |
| rs4545769 | 15 | 69490697 | 0.163 | -0.089 | 2.10E-04 | A | G | N | *LOC645296* |
| rs4777385 | 15 | 69489764 | 0.163 | -0.089 | 2.11E-04 | C | G | N | *LOC645296* |
| rs1158762 | 12 | 18895583 | 0.114 | 0.098 | 2.11E-04 | G | T | Y | *CAPZA3* |
| rs743657 | 16 | 13170152 | 0.454 | 0.063 | 2.11E-04 | T | C | N | *FLJ11151* |
| rs969183 | 8 | 55976760 | 0.285 | 0.070 | 2.12E-04 | G | A | N | *XKR4* |
| rs180994 | 3 | 60519776 | 0.296 | -0.072 | 2.12E-04 | T | C | N | *FHIT* |
| rs12545787 | 8 | 142168494 | 0.330 | 0.069 | 2.12E-04 | C | A | N | *DENND3* |
| rs4327580 | 5 | 105548751 | 0.037 | 0.167 | 2.13E-04 | A | G | N | *EFNA5* |
| rs8086262 | 18 | 67378570 | 0.315 | -0.069 | 2.13E-04 | G | A | N | *CBLN2* |
| rs9566553 | 13 | 40072231 | 0.311 | 0.068 | 2.14E-04 | G | A | N | *FOXO1* |
| rs6828489 | 4 | 145453965 | 0.296 | -0.069 | 2.14E-04 | T | C | Y | *GYPA* |
| rs12583418 | 13 | 40093411 | 0.311 | 0.068 | 2.14E-04 | A | G | N | *FOXO1* |
| rs7335520 | 13 | 40094407 | 0.311 | 0.068 | 2.15E-04 | T | C | N | *FOXO1* |
| rs1431441 | 20 | 12974863 | 0.353 | -0.066 | 2.15E-04 | T | G | Y | *SPTLC3* |
| rs4603422 | 13 | 40046353 | 0.310 | 0.068 | 2.16E-04 | C | T | N | *FOXO1* |
| rs4903960 | 14 | 80525460 | 0.096 | 0.109 | 2.16E-04 | G | A | N | *TSHR* |
| rs4943795 | 13 | 40071433 | 0.310 | 0.068 | 2.16E-04 | G | A | N | *FOXO1* |
| rs7074993 | 10 | 5510943 | 0.273 | -0.071 | 2.17E-04 | T | C | N | *CALML5* |
| rs11119583 | 1 | 208969817 | 0.236 | -0.074 | 2.18E-04 | A | C | N | *KCNH1* |
| rs7847568 | 9 | 7776145 | 0.023 | 0.210 | 2.18E-04 | C | T | N | *C9orf123* |
| rs7099350 | 10 | 5509005 | 0.273 | -0.071 | 2.18E-04 | T | C | N | *NET1* |
| rs10508438 | 10 | 12284085 | 0.324 | -0.069 | 2.18E-04 | A | G | N | *NUDT5* |
| rs7330614 | 13 | 40097421 | 0.311 | 0.068 | 2.18E-04 | A | G | N | *FOXO1* |
| rs6067243 | 20 | 47874914 | 0.203 | 0.078 | 2.18E-04 | A | C | N | *SLC9A8* |
| rs10079780 | 5 | 105503234 | 0.037 | 0.165 | 2.18E-04 | T | A | N | *EFNA5* |
| rs7078960 | 10 | 12629156 | 0.335 | 0.070 | 2.18E-04 | A | G | Y | *CAMK1D* |
| rs11827713 | 11 | 96982632 | 0.067 | -0.129 | 2.18E-04 | T | C | N | *JRKL* |
| rs2662826 | 12 | 126198334 | 0.106 | -0.101 | 2.19E-04 | A | G | Y | *TMEM132B* |
| rs4965598 | 15 | 98577137 | 0.317 | -0.069 | 2.19E-04 | C | T | N | *ADAMTS17* |
| rs7338669 | 13 | 40098091 | 0.311 | 0.068 | 2.19E-04 | G | A | N | *FOXO1* |
| rs6574616 | 14 | 80527336 | 0.095 | 0.109 | 2.19E-04 | T | C | N | *TSHR* |
| rs1329705 | 6 | 142795031 | 0.202 | 0.079 | 2.21E-04 | A | G | N | *GPR126* |
| rs2755219 | 13 | 40067625 | 0.310 | 0.068 | 2.21E-04 | G | A | N | *FOXO1* |
| rs9932411 | 16 | 52562664 | 0.407 | 0.064 | 2.21E-04 | C | T | Y | *FTO* |
| rs12901204 | 15 | 98573930 | 0.317 | -0.069 | 2.22E-04 | A | G | N | *ADAMTS17* |
| rs3006625 | 10 | 32449554 | 0.352 | 0.065 | 2.22E-04 | A | T | N | *KIF5B* |
| rs11741821 | 5 | 105472767 | 0.037 | 0.165 | 2.22E-04 | T | G | N | *EFNA5* |
| rs2721044 | 13 | 40060225 | 0.310 | 0.068 | 2.22E-04 | T | C | N | *FOXO1* |
| rs2907803 | 10 | 32449660 | 0.352 | 0.065 | 2.23E-04 | G | A | N | *KIF5B* |
| rs9549236 | 13 | 40045158 | 0.309 | 0.068 | 2.23E-04 | C | T | N | *FOXO1* |
| rs10976575 | 9 | 7777542 | 0.022 | 0.215 | 2.24E-04 | A | G | N | *C9orf123* |
| rs10812133 | 9 | 2490687 | 0.202 | 0.079 | 2.24E-04 | G | A | N | *VLDLR* |
| rs7993420 | 13 | 34350845 | 0.134 | -0.093 | 2.25E-04 | A | C | N | *NBEA* |
| rs4687657 | 3 | 52827578 | 0.268 | 0.073 | 2.25E-04 | T | G | N | *ITIH4* |
| rs2721066 | 13 | 40054188 | 0.310 | 0.068 | 2.25E-04 | C | T | Y | *FOXO1* |
| rs7777166 | 7 | 32362822 | 0.308 | -0.068 | 2.25E-04 | T | C | N | *LSM5* |
| rs2276815 | 3 | 52828787 | 0.268 | 0.073 | 2.25E-04 | G | C | N | *ITIH4* |
| rs998413 | 7 | 31990756 | 0.107 | 0.105 | 2.25E-04 | C | T | N | *PDE1C* |
| rs2907804 | 10 | 32450664 | 0.352 | 0.065 | 2.26E-04 | C | G | N | *KIF5B* |
| rs9423618 | 10 | 5500991 | 0.273 | -0.070 | 2.26E-04 | G | A | N | *NET1* |
| rs7128805 | 11 | 86163038 | 0.146 | -0.091 | 2.27E-04 | C | G | N | *PRSS23* |
| rs2367233 | 2 | 86395177 | 0.440 | 0.063 | 2.30E-04 | A | C | N | *REEP1* |
| rs17160742 | 7 | 31993813 | 0.107 | 0.104 | 2.30E-04 | G | T | N | *PDE1C* |
| rs7997592 | 13 | 34356662 | 0.134 | -0.092 | 2.32E-04 | T | C | N | *NBEA* |
| rs995267 | 7 | 31994550 | 0.107 | 0.104 | 2.32E-04 | C | A | N | *PDE1C* |
| rs7328677 | 13 | 40127905 | 0.301 | 0.070 | 2.32E-04 | G | A | N | *FOXO1* |
| rs17493316 | 10 | 12626943 | 0.308 | 0.068 | 2.32E-04 | G | C | Y | *CAMK1D* |
| rs11625251 | 14 | 80477644 | 0.103 | 0.107 | 2.32E-04 | G | C | N | *C14orf145* |
| rs1816468 | 11 | 54832793 | 0.166 | -0.084 | 2.36E-04 | C | A | N | *OR4A16* |
| rs1251363 | 10 | 32368532 | 0.488 | -0.062 | 2.37E-04 | C | G | N | *KIF5B* |
| rs1051879 | 12 | 115951508 | 0.012 | -0.293 | 2.38E-04 | A | G | N | *FBXW8* |
| rs1446749 | 2 | 134225205 | 0.171 | 0.082 | 2.39E-04 | C | G | Y | *NAP5* |
| rs6089707 | 20 | 60268960 | 0.066 | -0.125 | 2.39E-04 | T | C | N | *OSBPL2* |
| rs17362817 | 7 | 69068275 | 0.104 | 0.102 | 2.39E-04 | G | A | N | *AUTS2* |
| rs180998 | 3 | 60520642 | 0.305 | -0.070 | 2.40E-04 | A | G | N | *FHIT* |
| rs1388765 | 5 | 5981980 | 0.126 | 0.093 | 2.41E-04 | A | G | Y | *FLJ33360* |
| rs9414877 | 10 | 66648119 | 0.394 | 0.064 | 2.43E-04 | G | C | N | *CTNNA3* |
| rs10048364 | 18 | 33259479 | 0.306 | -0.070 | 2.43E-04 | G | A | N | *BRUNOL4* |
| rs10976573 | 9 | 7774277 | 0.023 | 0.203 | 2.44E-04 | C | T | Y | *C9orf123* |
| rs1562349 | 2 | 134211989 | 0.171 | 0.082 | 2.44E-04 | A | T | Y | *NAP5* |
| rs2440374 | 15 | 50884380 | 0.242 | -0.072 | 2.44E-04 | G | C | N | *ONECUT1* |
| rs2309741 | 9 | 72363501 | 0.558 | 0.063 | 2.45E-04 | A | G | N | *TRPM3* |
| rs1439569 | 5 | 124573447 | 0.404 | -0.063 | 2.46E-04 | A | T | Y | *ZNF608* |
| rs10778114 | 12 | 100269416 | 0.452 | 0.062 | 2.48E-04 | A | G | N | *UTP20* |
| rs17019336 | 4 | 145553059 | 0.253 | -0.071 | 2.48E-04 | A | T | Y | *HHIP* |
| rs7812790 | 8 | 55408323 | 0.248 | -0.072 | 2.48E-04 | A | G | N | *SOX17* |
| rs2165991 | 15 | 50888992 | 0.242 | -0.072 | 2.48E-04 | G | A | Y | *ONECUT1* |
| rs12623348 | 2 | 15806476 | 0.435 | 0.065 | 2.49E-04 | G | A | N | *DDX1* |
| rs10279512 | 7 | 13640880 | 0.531 | 0.063 | 2.50E-04 | C | A | Y | *ETV1* |
| rs12221133 | 10 | 12293603 | 0.227 | -0.077 | 2.51E-04 | A | G | N | *NUDT5* |
| rs7915870 | 10 | 66647891 | 0.394 | 0.064 | 2.51E-04 | A | T | N | *CTNNA3* |
| rs7896600 | 10 | 12295181 | 0.227 | -0.077 | 2.51E-04 | C | G | N | *NUDT5* |
| rs12653639 | 5 | 5983756 | 0.127 | 0.093 | 2.52E-04 | A | T | N | *FLJ33360* |
| rs1040525 | 6 | 142745362 | 0.298 | 0.068 | 2.53E-04 | T | C | Y | *GPR126* |
| rs2202509 | 5 | 90965179 | 0.516 | 0.064 | 2.53E-04 | G | A | N | *ARRDC3* |
| rs13294227 | 9 | 37772708 | 0.011 | -0.308 | 2.53E-04 | T | C | Y | *EXOSC3* |
| rs10453100 | 8 | 55963899 | 0.286 | 0.069 | 2.54E-04 | G | T | N | *XKR4* |
| rs7336674 | 13 | 34360987 | 0.134 | -0.092 | 2.54E-04 | A | G | N | *NBEA* |
| rs7895525 | 10 | 12290626 | 0.228 | -0.076 | 2.56E-04 | C | T | N | *NUDT5* |
| rs1992784 | 2 | 134207736 | 0.171 | 0.082 | 2.56E-04 | T | C | N | *NAP5* |
| rs17364237 | 7 | 69125203 | 0.104 | 0.102 | 2.57E-04 | T | C | N | *AUTS2* |
| rs11070517 | 15 | 44489426 | 0.312 | 0.068 | 2.58E-04 | A | G | N | *SQRDL* |
| rs1867894 | 2 | 134212517 | 0.171 | 0.082 | 2.58E-04 | T | C | N | *NAP5* |
| rs2175218 | 10 | 66645956 | 0.394 | 0.064 | 2.59E-04 | A | G | N | *CTNNA3* |
| rs1928160 | 6 | 22114655 | 0.529 | 0.061 | 2.59E-04 | G | A | N | *PRL* |
| rs4469792 | 10 | 12328786 | 0.585 | 0.063 | 2.59E-04 | G | A | N | *CDC123* |
| rs12692277 | 2 | 15806591 | 0.436 | 0.064 | 2.59E-04 | C | T | N | *DDX1* |
| rs225205 | 17 | 27918399 | 0.396 | 0.064 | 2.60E-04 | T | G | Y | *MYO1D* |
| rs11685485 | 2 | 164459010 | 0.267 | 0.071 | 2.60E-04 | T | G | N | *FIGN* |
| rs10778113 | 12 | 100266092 | 0.452 | 0.062 | 2.60E-04 | A | G | N | *UTP20* |
| rs1385073 | 15 | 44490900 | 0.311 | 0.067 | 2.61E-04 | A | G | Y | *SQRDL* |
| rs242089 | 22 | 31543319 | 0.531 | -0.062 | 2.61E-04 | A | G | N | *TIMP3* |
| rs6062175 | 20 | 60268887 | 0.067 | -0.124 | 2.61E-04 | T | A | N | *OSBPL2* |
| rs6547674 | 2 | 86406430 | 0.440 | 0.062 | 2.61E-04 | G | T | N | *REEP1* |
| rs7989140 | 13 | 34367729 | 0.134 | -0.091 | 2.61E-04 | G | C | Y | *NBEA* |
| rs1360950 | 10 | 89770130 | 0.255 | -0.071 | 2.61E-04 | A | G | Y | *PTEN* |
| rs1877256 | 3 | 73852192 | 0.398 | -0.063 | 2.62E-04 | A | G | Y | *PDZRN3* |
| rs10976571 | 9 | 7770039 | 0.023 | 0.203 | 2.63E-04 | T | C | Y | *C9orf123* |
| rs180993 | 3 | 60518906 | 0.306 | -0.070 | 2.64E-04 | C | G | N | *FHIT* |
| rs738992 | 22 | 31540005 | 0.531 | -0.062 | 2.64E-04 | C | T | N | *TIMP3* |
| rs1428814 | 16 | 56607070 | 0.216 | -0.077 | 2.64E-04 | T | G | N | *C16orf57* |
| rs10502622 | 18 | 29538006 | 0.089 | 0.109 | 2.64E-04 | G | A | N | *ASXL3* |
| rs11127029 | 2 | 86408136 | 0.440 | 0.062 | 2.65E-04 | C | G | N | *REEP1* |
| rs180992 | 3 | 60518255 | 0.305 | -0.070 | 2.65E-04 | C | G | N | *FHIT* |
| rs6990967 | 8 | 41804027 | 0.425 | 0.065 | 2.65E-04 | G | T | N | *ANK1* |
| rs11683609 | 2 | 164456742 | 0.267 | 0.071 | 2.65E-04 | G | A | N | *FIGN* |
| rs1992783 | 2 | 134207467 | 0.171 | 0.081 | 2.65E-04 | T | G | N | *NAP5* |
| rs2399797 | 10 | 12329624 | 0.585 | 0.063 | 2.65E-04 | T | C | N | *CDC123* |
| rs1821849 | 2 | 164456018 | 0.267 | 0.071 | 2.66E-04 | T | C | N | *FIGN* |
| rs877640 | 9 | 111703346 | 0.380 | 0.064 | 2.68E-04 | C | T | Y | *PALM2* |
| rs3848249 | 16 | 83586185 | 0.372 | -0.063 | 2.69E-04 | C | T | Y | *ZDHHC7* |
| rs4369917 | 2 | 134205342 | 0.171 | 0.081 | 2.70E-04 | T | C | N | *NAP5* |
| rs1370488 | 2 | 164453822 | 0.267 | 0.071 | 2.71E-04 | T | C | N | *FIGN* |
| rs9682464 | 3 | 52863525 | 0.236 | 0.073 | 2.71E-04 | T | C | N | *TMEM110* |
| rs2276825 | 3 | 52861645 | 0.237 | 0.073 | 2.72E-04 | C | T | N | *TMEM110* |
| rs4581585 | 13 | 40101905 | 0.311 | 0.067 | 2.72E-04 | T | C | N | *FOXO1* |
| rs4943798 | 13 | 40100948 | 0.311 | 0.067 | 2.73E-04 | C | T | N | *FOXO1* |
| rs4943797 | 13 | 40099633 | 0.311 | 0.067 | 2.73E-04 | A | G | Y | *FOXO1* |
| rs16876618 | 5 | 5984649 | 0.127 | 0.092 | 2.74E-04 | T | A | N | *FLJ33360* |
| rs2673930 | 5 | 135719577 | 0.290 | 0.068 | 2.75E-04 | G | T | N | *TRPC7* |
| rs7333037 | 13 | 40113256 | 0.311 | 0.067 | 2.75E-04 | T | A | N | *FOXO1* |
| rs10215894 | 7 | 31923777 | 0.101 | -0.108 | 2.75E-04 | A | G | N | *PDE1C* |
| rs7989711 | 13 | 40113696 | 0.311 | 0.067 | 2.75E-04 | T | C | N | *FOXO1* |
| rs7988604 | 13 | 40113802 | 0.311 | 0.067 | 2.76E-04 | T | G | N | *FOXO1* |
| rs4325426 | 13 | 40114437 | 0.311 | 0.067 | 2.77E-04 | A | C | N | *FOXO1* |
| rs9927546 | 16 | 79867508 | 0.264 | -0.070 | 2.77E-04 | G | A | N | *BCMO1* |
| rs8001443 | 13 | 40114646 | 0.311 | 0.067 | 2.77E-04 | C | T | Y | *FOXO1* |
| rs7279660 | 21 | 39158680 | 0.504 | -0.062 | 2.77E-04 | G | A | N | *FLJ45139* |
| rs6062177 | 20 | 60276328 | 0.066 | -0.123 | 2.78E-04 | A | G | Y | *OSBPL2* |
| rs2569821 | 12 | 126178939 | 0.103 | -0.102 | 2.78E-04 | T | C | N | *TMEM132B* |
| rs2662819 | 12 | 126179768 | 0.103 | -0.101 | 2.78E-04 | C | A | Y | *TMEM132B* |
| rs1042714 | 5 | 148186666 | 0.420 | -0.062 | 2.79E-04 | G | C | Y | *ADRB2* |
| rs17569373 | 18 | 33247837 | 0.308 | -0.070 | 2.79E-04 | C | T | N | *BRUNOL4* |
| rs7499777 | 16 | 79867238 | 0.264 | -0.070 | 2.79E-04 | G | A | N | *BCMO1* |
| rs7317254 | 13 | 40115849 | 0.311 | 0.067 | 2.80E-04 | G | A | N | *FOXO1* |
| rs9973849 | 2 | 164442433 | 0.267 | 0.071 | 2.80E-04 | C | T | N | *FIGN* |
| rs17605839 | 2 | 134218454 | 0.171 | 0.081 | 2.80E-04 | T | G | N | *NAP5* |
| rs2721071 | 13 | 40042507 | 0.308 | 0.067 | 2.80E-04 | A | G | N | *FOXO1* |
| rs10239445 | 7 | 13640670 | 0.531 | 0.062 | 2.81E-04 | G | A | Y | *ETV1* |
| rs9842536 | 3 | 41372045 | 0.211 | 0.079 | 2.81E-04 | T | C | N | *ULK4* |
| rs12468301 | 2 | 134218729 | 0.171 | 0.081 | 2.82E-04 | G | T | N | *NAP5* |
| rs1923249 | 13 | 40041881 | 0.308 | 0.067 | 2.82E-04 | A | C | N | *FOXO1* |
| rs2154659 | 8 | 110653350 | 0.063 | -0.127 | 2.82E-04 | C | T | Y | *GOLSYN* |
| rs12468376 | 2 | 134218908 | 0.171 | 0.081 | 2.82E-04 | C | T | N | *NAP5* |
| rs4847378 | 1 | 93097222 | 0.388 | 0.063 | 2.83E-04 | G | T | N | *RPL5* |
| rs2609695 | 14 | 80335010 | 0.105 | 0.103 | 2.83E-04 | C | T | N | *C14orf145* |
| rs10507484 | 13 | 40116134 | 0.311 | 0.067 | 2.83E-04 | C | T | N | *FOXO1* |
| rs2721069 | 13 | 40041720 | 0.308 | 0.067 | 2.84E-04 | T | C | N | *FOXO1* |
| rs6490977 | 13 | 24222303 | 0.346 | -0.066 | 2.84E-04 | G | T | N | *RNF17* |
| rs7994316 | 13 | 24222895 | 0.346 | -0.066 | 2.84E-04 | C | T | N | *RNF17* |
| rs2297627 | 13 | 40131931 | 0.311 | 0.067 | 2.85E-04 | G | A | Y | *FOXO1* |
| rs9532571 | 13 | 40116670 | 0.311 | 0.067 | 2.85E-04 | T | C | Y | *FOXO1* |
| rs9549244 | 13 | 40116685 | 0.311 | 0.067 | 2.85E-04 | A | G | Y | *FOXO1* |
| rs7993233 | 13 | 40117434 | 0.311 | 0.067 | 2.85E-04 | A | T | N | *FOXO1* |
| rs9577091 | 13 | 40125865 | 0.311 | 0.067 | 2.85E-04 | A | G | N | *FOXO1* |
| rs9549252 | 13 | 40128141 | 0.311 | 0.067 | 2.85E-04 | C | T | Y | *FOXO1* |
| rs12640763 | 4 | 145455106 | 0.306 | -0.069 | 2.85E-04 | C | T | N | *GYPA* |
| rs2721072 | 13 | 40042919 | 0.305 | 0.068 | 2.86E-04 | A | G | N | *FOXO1* |
| rs581456 | 20 | 47899845 | 0.203 | 0.076 | 2.86E-04 | A | T | N | *SLC9A8* |
| rs17330985 | 10 | 89972718 | 0.052 | 0.138 | 2.86E-04 | C | T | Y | *C10orf59* |
| rs2025977 | 6 | 22103020 | 0.323 | 0.067 | 2.86E-04 | C | A | N | *PRL* |
| rs6802512 | 3 | 73790711 | 0.351 | -0.065 | 2.87E-04 | A | C | N | *PDZRN3* |
| rs1941946 | 18 | 33246552 | 0.308 | -0.070 | 2.87E-04 | T | C | N | *BRUNOL4* |
| rs9549255 | 13 | 40133213 | 0.311 | 0.067 | 2.87E-04 | T | C | N | *FOXO1* |
| rs16828728 | 2 | 134224718 | 0.171 | 0.081 | 2.88E-04 | T | C | Y | *NAP5* |
| rs10496709 | 2 | 134223635 | 0.171 | 0.081 | 2.88E-04 | T | C | Y | *NAP5* |
| rs16828705 | 2 | 134224252 | 0.171 | 0.081 | 2.88E-04 | T | A | N | *NAP5* |
| rs1668393 | 7 | 32360136 | 0.318 | -0.068 | 2.88E-04 | C | T | N | *LSM5* |
| rs720212 | 1 | 34343793 | 0.425 | -0.063 | 2.89E-04 | T | C | N | *CSMD2* |
| rs10874746 | 1 | 93096559 | 0.384 | 0.062 | 2.89E-04 | T | C | Y | *RPL5* |
| rs7718593 | 5 | 96014248 | 0.095 | -0.105 | 2.89E-04 | G | A | N | *CAST* |
| rs720211 | 1 | 34343731 | 0.425 | -0.063 | 2.90E-04 | C | T | N | *CSMD2* |
| rs9620594 | 22 | 24955605 | 0.034 | 0.172 | 2.90E-04 | G | C | N | *SEZ6L* |
| rs1916362 | 11 | 54638724 | 0.167 | -0.083 | 2.91E-04 | G | A | N | *TRIM48* |
| rs180987 | 3 | 60517363 | 0.300 | -0.070 | 2.91E-04 | C | G | N | *FHIT* |
| rs7292846 | 22 | 24956242 | 0.034 | 0.172 | 2.91E-04 | G | A | N | *SEZ6L* |
| rs17061503 | 13 | 40144758 | 0.311 | 0.067 | 2.91E-04 | A | G | Y | *FOXO1* |
| rs10487941 | 7 | 69160457 | 0.103 | 0.101 | 2.91E-04 | C | T | N | *AUTS2* |
| rs10068316 | 5 | 5991780 | 0.128 | 0.092 | 2.91E-04 | A | G | N | *FLJ33360* |
| rs2053217 | 1 | 34344258 | 0.425 | -0.063 | 2.93E-04 | G | T | N | *CSMD2* |
| rs9624998 | 22 | 24954155 | 0.034 | 0.172 | 2.94E-04 | T | C | N | *SEZ6L* |
| rs12054664 | 5 | 5990537 | 0.128 | 0.092 | 2.94E-04 | T | C | Y | *FLJ33360* |
| rs4723110 | 7 | 31919766 | 0.125 | 0.093 | 2.95E-04 | C | G | N | *PDE1C* |
| rs1935502 | 10 | 18372118 | 0.275 | 0.071 | 2.95E-04 | A | G | N | *SLC39A12* |
| rs4832033 | 2 | 86362274 | 0.485 | 0.061 | 2.95E-04 | T | A | N | *REEP1* |
| rs11623591 | 14 | 56605062 | 0.309 | 0.068 | 2.95E-04 | A | G | N | *EXOC5* |
| rs585976 | 20 | 47924311 | 0.205 | 0.077 | 2.95E-04 | T | G | N | *SLC9A8* |
| rs2239613 | 1 | 166550951 | 0.455 | 0.063 | 2.96E-04 | G | A | N | *TBX19* |
| rs9496346 | 6 | 142711031 | 0.297 | 0.067 | 2.96E-04 | G | A | N | *GPR126* |
| rs542234 | 20 | 47924665 | 0.205 | 0.077 | 2.96E-04 | T | G | N | *SLC9A8* |
| rs12054663 | 5 | 5990485 | 0.128 | 0.091 | 2.98E-04 | A | C | N | *FLJ33360* |
| rs2087753 | 5 | 5988502 | 0.128 | 0.092 | 2.98E-04 | A | G | N | *FLJ33360* |
| rs17820139 | 11 | 86166659 | 0.146 | -0.090 | 2.98E-04 | G | A | N | *PRSS23* |
| rs676035 | 20 | 47916399 | 0.205 | 0.077 | 2.98E-04 | G | A | N | *SLC9A8* |
| rs17374170 | 12 | 12255678 | 0.064 | -0.125 | 2.98E-04 | C | T | N | *BCL2L14* |
| rs17245026 | 11 | 74390579 | 0.068 | 0.123 | 3.00E-04 | A | G | N | *NEU3* |
| rs13111399 | 4 | 150161100 | 0.266 | 0.069 | 3.00E-04 | T | C | Y | *NR3C2* |
| rs12791740 | 11 | 86114396 | 0.154 | -0.087 | 3.00E-04 | G | A | N | *ME3* |
| rs673010 | 20 | 47910452 | 0.204 | 0.076 | 3.00E-04 | A | G | N | *SLC9A8* |
| rs17758334 | 11 | 86166716 | 0.146 | -0.090 | 3.00E-04 | G | A | N | *PRSS23* |
| rs4392535 | 4 | 41208180 | 0.280 | 0.068 | 3.01E-04 | A | G | N | *LIMCH1* |
| rs6480780 | 10 | 76785960 | 0.337 | 0.065 | 3.01E-04 | C | T | N | *ZNF503* |
| rs13173909 | 5 | 5989111 | 0.128 | 0.091 | 3.01E-04 | T | C | N | *FLJ33360* |
| rs532237 | 20 | 47900967 | 0.204 | 0.076 | 3.02E-04 | G | C | N | *SLC9A8* |
| rs7910950 | 10 | 66615669 | 0.391 | 0.063 | 3.03E-04 | T | C | N | *CTNNA3* |
| rs1972074 | 12 | 126325828 | 0.272 | -0.069 | 3.05E-04 | G | A | Y | *SLC15A4* |
| rs12304673 | 12 | 12329297 | 0.061 | -0.130 | 3.05E-04 | G | A | N | *LRP6* |
| rs10275337 | 7 | 51604145 | 0.433 | 0.062 | 3.05E-04 | C | G | N | *COBL* |
| rs1668389 | 7 | 32353440 | 0.318 | -0.067 | 3.06E-04 | G | T | N | *LSM5* |
| rs6078905 | 20 | 12982705 | 0.354 | -0.064 | 3.07E-04 | C | T | Y | *SPTLC3* |
| rs1546454 | 12 | 126325752 | 0.272 | -0.069 | 3.07E-04 | G | A | N | *SLC15A4* |
| rs10493863 | 1 | 93172282 | 0.363 | 0.063 | 3.08E-04 | C | T | N | *FAM69A* |
| rs17721066 | 14 | 56609920 | 0.308 | 0.068 | 3.08E-04 | C | T | N | *EXOC5* |
| rs2520494 | 4 | 150149136 | 0.266 | 0.069 | 3.08E-04 | T | C | N | *NR3C2* |
| rs17133608 | 11 | 74401324 | 0.068 | 0.122 | 3.08E-04 | T | G | Y | *NEU3* |
| rs1653889 | 7 | 32353178 | 0.318 | -0.067 | 3.08E-04 | G | A | N | *LSM5* |
| rs7683275 | 4 | 41221779 | 0.281 | 0.068 | 3.08E-04 | G | A | Y | *LIMCH1* |
| rs6062207 | 20 | 60312623 | 0.064 | -0.127 | 3.09E-04 | G | A | N | *ADRM1* |
| rs4246010 | 5 | 111620253 | 0.456 | 0.061 | 3.10E-04 | A | T | N | *EPB41L4A* |
| rs1322023 | 6 | 17155296 | 0.130 | -0.093 | 3.10E-04 | A | G | N | *RBM24* |
| rs4947374 | 7 | 51604357 | 0.433 | 0.062 | 3.11E-04 | G | A | N | *COBL* |
| rs6957512 | 7 | 31944481 | 0.125 | 0.093 | 3.11E-04 | T | G | N | *PDE1C* |
| rs6977395 | 7 | 31944556 | 0.125 | 0.093 | 3.11E-04 | C | T | N | *PDE1C* |
| rs3006630 | 10 | 32455017 | 0.351 | 0.063 | 3.12E-04 | T | A | N | *KIF5B* |
| rs10875076 | 1 | 97610152 | 0.238 | -0.073 | 3.13E-04 | T | C | Y | *DPYD* |
| rs989277 | 1 | 34346358 | 0.425 | -0.062 | 3.13E-04 | C | T | Y | *CSMD2* |
| rs4947375 | 7 | 51604696 | 0.434 | 0.062 | 3.14E-04 | G | A | N | *COBL* |
| rs750276 | 12 | 126326169 | 0.272 | -0.068 | 3.15E-04 | T | C | Y | *SLC15A4* |
| rs3006631 | 10 | 32455670 | 0.351 | 0.063 | 3.15E-04 | T | C | N | *KIF5B* |
| rs7826221 | 8 | 41787859 | 0.439 | 0.062 | 3.15E-04 | G | A | N | *ANK1* |
| rs10075926 | 5 | 111618140 | 0.456 | 0.061 | 3.15E-04 | G | T | N | *EPB41L4A* |
| rs11099714 | 4 | 150161253 | 0.266 | 0.069 | 3.16E-04 | T | C | Y | *NR3C2* |
| rs1890138 | 1 | 97611604 | 0.238 | -0.073 | 3.16E-04 | G | A | N | *DPYD* |
| rs3006633 | 10 | 32455811 | 0.351 | 0.063 | 3.17E-04 | A | G | N | *KIF5B* |
| rs12166208 | 22 | 24964150 | 0.034 | 0.171 | 3.18E-04 | A | G | N | *SEZ6L* |
| rs17746706 | 18 | 29546952 | 0.089 | 0.108 | 3.18E-04 | T | C | N | *ASXL3* |
| rs2174731 | 8 | 41797284 | 0.444 | 0.062 | 3.18E-04 | C | G | N | *ANK1* |
| rs10030023 | 4 | 145478257 | 0.292 | -0.070 | 3.18E-04 | A | C | N | *GYPA* |
| rs3006635 | 10 | 32455925 | 0.351 | 0.063 | 3.18E-04 | G | A | N | *KIF5B* |
| rs1407287 | 6 | 17154550 | 0.130 | -0.092 | 3.19E-04 | T | A | N | *RBM24* |
| rs9403383 | 6 | 142735742 | 0.296 | 0.067 | 3.20E-04 | A | G | N | *GPR126* |
| rs12166576 | 22 | 24964163 | 0.034 | 0.171 | 3.20E-04 | T | C | N | *SEZ6L* |
| rs1415683 | 1 | 97617641 | 0.237 | -0.073 | 3.20E-04 | G | T | N | *DPYD* |
| rs10949748 | 7 | 158447962 | 0.165 | 0.082 | 3.20E-04 | C | T | Y | *WDR60* |
| rs10847306 | 12 | 126325244 | 0.281 | -0.069 | 3.22E-04 | G | T | N | *SLC15A4* |
| rs17753444 | 18 | 29552329 | 0.089 | 0.108 | 3.22E-04 | C | T | N | *ASXL3* |
| rs6926620 | 6 | 22103525 | 0.318 | 0.066 | 3.22E-04 | C | G | N | *PRL* |
| rs17287920 | 11 | 96973883 | 0.065 | -0.127 | 3.22E-04 | C | G | N | *JRKL* |
| rs10996280 | 10 | 66633503 | 0.394 | 0.063 | 3.23E-04 | T | C | N | *CTNNA3* |
| rs7244544 | 18 | 24481345 | 0.282 | 0.071 | 3.23E-04 | A | G | N | *CDH2* |
| rs2400707 | 5 | 148185245 | 0.421 | -0.061 | 3.23E-04 | A | G | N | *ADRB2* |
| rs2005358 | 8 | 142173290 | 0.325 | 0.068 | 3.23E-04 | A | G | N | *DENND3* |
| rs9884270 | 4 | 58465420 | 0.101 | -0.102 | 3.24E-04 | G | A | N | *IGFBP7* |
| rs17460898 | 7 | 69091381 | 0.117 | 0.095 | 3.24E-04 | G | A | N | *AUTS2* |
| rs17363468 | 7 | 69090051 | 0.117 | 0.095 | 3.26E-04 | T | C | N | *AUTS2* |
| rs6824915 | 4 | 56401380 | 0.317 | 0.066 | 3.28E-04 | T | C | N | *EXOC1* |
| rs9553433 | 13 | 24225320 | 0.282 | -0.069 | 3.29E-04 | C | T | N | *RNF17* |
| rs6554325 | 4 | 56401644 | 0.317 | 0.066 | 3.29E-04 | C | G | N | *EXOC1* |
| rs225212 | 17 | 27920568 | 0.396 | 0.062 | 3.29E-04 | T | C | Y | *MYO1D* |
| rs2134823 | 2 | 163865853 | 0.035 | -0.166 | 3.29E-04 | A | G | N | *FIGN* |
| rs7536563 | 1 | 93121634 | 0.385 | 0.062 | 3.30E-04 | A | G | Y | *RPL5* |
| rs7969124 | 12 | 12329459 | 0.061 | -0.130 | 3.30E-04 | T | C | N | *LRP6* |
| rs17768541 | 2 | 134245880 | 0.170 | 0.081 | 3.30E-04 | C | T | N | *NAP5* |
| rs11246618 | 11 | 51383794 | 0.167 | -0.082 | 3.31E-04 | G | A | N | *OR4C46* |
| rs10902366 | 11 | 51383586 | 0.167 | -0.082 | 3.31E-04 | G | T | N | *OR4C46* |
| rs1259604 | 10 | 76789071 | 0.337 | 0.064 | 3.33E-04 | G | A | Y | *ZNF503* |
| rs10248935 | 7 | 51618163 | 0.434 | 0.062 | 3.34E-04 | T | G | Y | *COBL* |
| rs7114213 | 11 | 26025704 | 0.109 | 0.097 | 3.34E-04 | C | T | N | *TMEM16C* |
| rs726730 | 6 | 22104087 | 0.473 | 0.060 | 3.35E-04 | A | G | N | *PRL* |
| rs1221517 | 2 | 163881316 | 0.035 | -0.166 | 3.35E-04 | T | C | Y | *FIGN* |
| rs1426388 | 11 | 115479780 | 0.228 | 0.074 | 3.35E-04 | G | A | N | *CADM1* |
| rs7951445 | 11 | 26025857 | 0.109 | 0.097 | 3.35E-04 | T | G | N | *TMEM16C* |
| rs575013 | 11 | 85128084 | 0.456 | 0.061 | 3.36E-04 | A | C | Y | *SYTL2* |
| rs6509556 | 19 | 56786328 | 0.157 | -0.084 | 3.36E-04 | C | T | Y | *ZNF175* |
| rs3847268 | 9 | 7833394 | 0.456 | -0.061 | 3.36E-04 | A | C | N | *C9orf123* |
| rs987003 | 10 | 66626969 | 0.394 | 0.062 | 3.36E-04 | T | C | N | *CTNNA3* |
| rs7789277 | 7 | 31926742 | 0.125 | 0.092 | 3.36E-04 | G | A | N | *PDE1C* |
| rs7800709 | 7 | 51618290 | 0.434 | 0.062 | 3.36E-04 | C | T | N | *COBL* |
| rs4949966 | 1 | 95741623 | 0.124 | 0.092 | 3.36E-04 | C | T | Y | *RWDD3* |
| rs10874747 | 1 | 93125079 | 0.385 | 0.062 | 3.36E-04 | A | G | N | *RPL5* |
| rs1432622 | 5 | 148183955 | 0.421 | -0.061 | 3.37E-04 | T | C | N | *ADRB2* |
| rs17054760 | 5 | 156968824 | 0.166 | 0.084 | 3.37E-04 | A | G | N | *SOX30* |
| rs2488115 | 10 | 18349036 | 0.294 | 0.067 | 3.38E-04 | G | C | N | *SLC39A12* |
| rs12983168 | 19 | 33693476 | 0.395 | 0.063 | 3.39E-04 | A | T | Y | *UQCRFS1* |
| rs8059061 | 16 | 75256988 | 0.168 | -0.081 | 3.39E-04 | C | A | N | *CNTNAP4* |
| rs9968744 | 5 | 156968488 | 0.166 | 0.084 | 3.39E-04 | T | C | N | *SOX30* |
| rs11860119 | 16 | 75270852 | 0.168 | -0.081 | 3.40E-04 | T | C | N | *CNTNAP4* |
| rs8047051 | 16 | 75280001 | 0.168 | -0.081 | 3.41E-04 | A | G | Y | *CNTNAP4* |
| rs11648487 | 16 | 13168090 | 0.441 | 0.061 | 3.41E-04 | G | C | N | *FLJ11151* |
| rs8050006 | 16 | 75280554 | 0.168 | -0.081 | 3.41E-04 | C | A | Y | *CNTNAP4* |
| rs17495 | 14 | 56611714 | 0.308 | 0.068 | 3.41E-04 | G | A | N | *EXOC5* |
| rs9321345 | 6 | 132836468 | 0.494 | -0.062 | 3.42E-04 | T | C | N | *STX7* |
| rs2191253 | 7 | 51619367 | 0.434 | 0.061 | 3.42E-04 | G | T | Y | *COBL* |
| rs6964930 | 7 | 51620560 | 0.434 | 0.061 | 3.43E-04 | T | A | N | *COBL* |
| rs4428627 | 7 | 51620909 | 0.434 | 0.061 | 3.43E-04 | C | T | Y | *COBL* |
| rs1570647 | 10 | 105829993 | 0.108 | -0.097 | 3.43E-04 | T | C | N | *COL17A1* |
| rs2876955 | 7 | 51621046 | 0.434 | 0.061 | 3.43E-04 | T | C | N | *COBL* |
| rs850786 | 12 | 100196224 | 0.452 | 0.061 | 3.43E-04 | G | A | N | *UTP20* |
| rs10014018 | 4 | 56457927 | 0.319 | 0.066 | 3.43E-04 | A | G | N | *EXOC1* |
| rs6945118 | 7 | 51621340 | 0.434 | 0.061 | 3.43E-04 | G | A | Y | *COBL* |
| rs10782946 | 1 | 93125257 | 0.385 | 0.062 | 3.43E-04 | G | A | N | *RPL5* |
| rs11191916 | 10 | 105822340 | 0.108 | -0.097 | 3.43E-04 | T | C | Y | *COL17A1* |
| rs2215331 | 7 | 51621534 | 0.434 | 0.061 | 3.45E-04 | A | G | N | *COBL* |
| rs242088 | 22 | 31549887 | 0.531 | -0.061 | 3.46E-04 | T | C | Y | *TIMP3* |
| rs2191252 | 7 | 51621757 | 0.434 | 0.061 | 3.46E-04 | T | C | N | *COBL* |
| rs17054759 | 5 | 156967166 | 0.166 | 0.084 | 3.46E-04 | G | A | N | *SOX30* |
| rs7114240 | 11 | 51348231 | 0.167 | -0.082 | 3.46E-04 | T | C | N | *OR4C46* |
| rs8040015 | 15 | 69491374 | 0.227 | -0.076 | 3.47E-04 | T | C | N | *LOC645296* |
| rs1360366 | 1 | 93103575 | 0.384 | 0.062 | 3.47E-04 | A | C | N | *RPL5* |
| rs2018894 | 6 | 170176039 | 0.356 | -0.065 | 3.48E-04 | T | C | N | *C6orf70* |
| rs11733975 | 4 | 145605923 | 0.301 | -0.067 | 3.48E-04 | C | T | N | *HHIP* |
| rs12334878 | 8 | 142163959 | 0.368 | 0.063 | 3.50E-04 | C | T | Y | *DENND3* |
| rs2453823 | 5 | 91040917 | 0.481 | -0.062 | 3.50E-04 | A | T | N | *ARRDC3* |
| rs6822760 | 4 | 58463415 | 0.101 | -0.101 | 3.50E-04 | A | G | N | *IGFBP7* |
| rs10435958 | 9 | 72376919 | 0.446 | -0.062 | 3.50E-04 | T | A | N | *TRPM3* |
| rs242085 | 22 | 31551030 | 0.530 | -0.061 | 3.51E-04 | C | T | N | *TIMP3* |
| rs13405740 | 2 | 235019660 | 0.334 | -0.065 | 3.52E-04 | G | C | N | *ARL4C* |
| rs2381670 | 9 | 7844575 | 0.458 | -0.061 | 3.53E-04 | A | G | N | *C9orf123* |
| rs4498257 | 5 | 43452780 | 0.022 | 0.205 | 3.53E-04 | G | A | N | *CCL28* |
| rs11696906 | 20 | 1581018 | 0.051 | 0.138 | 3.54E-04 | C | T | N | *SIRPG* |
| rs9915089 | 17 | 8893619 | 0.312 | 0.068 | 3.54E-04 | T | C | N | *NTN1* |
| rs17019370 | 4 | 145601216 | 0.301 | -0.067 | 3.54E-04 | C | A | N | *HHIP* |
| rs16873319 | 5 | 43454277 | 0.022 | 0.205 | 3.55E-04 | G | A | Y | *CCL28* |
| rs4264950 | 5 | 43453006 | 0.022 | 0.205 | 3.55E-04 | C | T | N | *CCL28* |
| rs7720858 | 5 | 43455616 | 0.022 | 0.205 | 3.55E-04 | A | C | Y | *CCL28* |
| rs2381671 | 9 | 7844722 | 0.458 | -0.061 | 3.56E-04 | A | G | N | *C9orf123* |
| rs1415563 | 1 | 95719925 | 0.124 | 0.092 | 3.56E-04 | A | G | Y | *RWDD3* |
| rs4847384 | 1 | 93154867 | 0.385 | 0.061 | 3.57E-04 | T | C | N | *FAM69A* |
| rs12409850 | 1 | 95720408 | 0.124 | 0.092 | 3.58E-04 | G | A | N | *RWDD3* |
| rs7756305 | 6 | 142712648 | 0.295 | 0.066 | 3.58E-04 | C | T | N | *GPR126* |
| rs4754879 | 11 | 102127709 | 0.080 | -0.112 | 3.59E-04 | T | C | N | *MMP10* |
| rs6604031 | 1 | 93173354 | 0.385 | 0.061 | 3.59E-04 | T | G | N | *FAM69A* |
| rs3741639 | 12 | 126325220 | 0.280 | -0.069 | 3.60E-04 | G | A | N | *SLC15A4* |
| rs9399401 | 6 | 142710594 | 0.295 | 0.066 | 3.61E-04 | C | T | N | *GPR126* |
| rs9321870 | 6 | 142842451 | 0.225 | 0.073 | 3.61E-04 | A | T | Y | *GPR126* |
| rs972982 | 6 | 142708554 | 0.295 | 0.066 | 3.62E-04 | C | T | N | *GPR126* |
| rs6781403 | 3 | 115587592 | 0.293 | -0.066 | 3.64E-04 | T | C | Y | *ZBTB20* |
| rs5009058 | 11 | 51315633 | 0.167 | -0.081 | 3.64E-04 | A | G | N | *OR4A5* |
| rs2721068 | 13 | 40037712 | 0.257 | 0.069 | 3.66E-04 | C | T | N | *FOXO1* |
| rs1342060 | 6 | 142704870 | 0.295 | 0.066 | 3.66E-04 | C | T | N | *GPR126* |
| rs2412692 | 4 | 56413069 | 0.320 | 0.065 | 3.67E-04 | C | T | Y | *EXOC1* |
| rs4923337 | 11 | 26025878 | 0.109 | 0.096 | 3.67E-04 | C | T | N | *TMEM16C* |
| rs13118613 | 4 | 37962196 | 0.348 | -0.064 | 3.68E-04 | A | G | N | *TBC1D1* |
| rs7818962 | 8 | 41781576 | 0.436 | 0.061 | 3.69E-04 | T | A | N | *ANK1* |
| rs13119207 | 4 | 37961802 | 0.348 | -0.064 | 3.69E-04 | T | C | Y | *TBC1D1* |
| rs9366421 | 6 | 22126017 | 0.467 | 0.060 | 3.69E-04 | G | C | N | *PRL* |
| rs6570507 | 6 | 142721265 | 0.294 | 0.066 | 3.70E-04 | A | G | N | *GPR126* |
| rs12803371 | 11 | 26026617 | 0.109 | 0.096 | 3.70E-04 | A | G | N | *TMEM16C* |
| rs12785755 | 11 | 26026637 | 0.109 | 0.096 | 3.70E-04 | T | C | Y | *TMEM16C* |
| rs1979624 | 11 | 26026372 | 0.109 | 0.096 | 3.70E-04 | G | A | N | *TMEM16C* |
| rs12803860 | 11 | 26026607 | 0.109 | 0.096 | 3.70E-04 | G | A | N | *TMEM16C* |
| rs1979622 | 11 | 26025929 | 0.109 | 0.096 | 3.70E-04 | G | A | Y | *TMEM16C* |
| rs1979623 | 11 | 26026236 | 0.109 | 0.096 | 3.70E-04 | G | A | N | *TMEM16C* |
| rs2701859 | 13 | 40039232 | 0.257 | 0.069 | 3.70E-04 | C | T | N | *FOXO1* |
| rs7818173 | 8 | 41781094 | 0.436 | 0.061 | 3.72E-04 | G | A | N | *ANK1* |
| rs9356791 | 6 | 22126560 | 0.467 | 0.060 | 3.72E-04 | G | A | Y | *PRL* |
| rs7923844 | 10 | 66625100 | 0.394 | 0.062 | 3.73E-04 | C | T | N | *CTNNA3* |
| rs10504044 | 8 | 41802781 | 0.429 | 0.063 | 3.73E-04 | G | C | N | *ANK1* |
| rs13272350 | 8 | 41780873 | 0.436 | 0.061 | 3.74E-04 | C | T | N | *ANK1* |
| rs16945076 | 18 | 24476122 | 0.264 | 0.069 | 3.74E-04 | G | A | N | *CDH2* |
| rs1484217 | 18 | 68610550 | 0.384 | -0.064 | 3.75E-04 | A | G | N | *NETO1* |
| rs6927921 | 6 | 156717341 | 0.175 | -0.079 | 3.75E-04 | A | G | N | *ARID1B* |
| rs10874923 | 1 | 95733387 | 0.124 | 0.092 | 3.77E-04 | C | T | N | *RWDD3* |
| rs6915211 | 6 | 156721020 | 0.175 | -0.079 | 3.77E-04 | C | G | N | *ARID1B* |
| rs10834898 | 11 | 26023571 | 0.151 | 0.086 | 3.77E-04 | G | T | N | *TMEM16C* |
| rs2349796 | 11 | 26026902 | 0.110 | 0.096 | 3.79E-04 | T | C | N | *TMEM16C* |
| rs6855889 | 4 | 166524104 | 0.196 | 0.077 | 3.79E-04 | T | C | Y | *CPE* |
| rs17019365 | 4 | 145592584 | 0.300 | -0.067 | 3.81E-04 | G | A | N | *HHIP* |
| rs2899039 | 4 | 56397056 | 0.436 | -0.061 | 3.81E-04 | A | G | Y | *EXOC1* |
| rs4663137 | 2 | 235022422 | 0.327 | -0.064 | 3.81E-04 | G | A | Y | *ARL4C* |
| rs6850000 | 4 | 166523622 | 0.196 | 0.077 | 3.82E-04 | G | A | N | *CPE* |
| rs4302374 | 3 | 52880842 | 0.232 | 0.072 | 3.82E-04 | C | T | N | *TMEM110* |
| rs2375261 | 8 | 55980521 | 0.329 | 0.064 | 3.82E-04 | C | G | N | *XKR4* |
| rs4663138 | 2 | 235022618 | 0.327 | -0.064 | 3.83E-04 | A | G | N | *ARL4C* |
| rs10822476 | 10 | 66594977 | 0.395 | 0.062 | 3.83E-04 | A | G | Y | *CTNNA3* |
| rs4663139 | 2 | 235024176 | 0.327 | -0.064 | 3.83E-04 | C | T | N | *ARL4C* |
| rs6445547 | 3 | 52883617 | 0.232 | 0.072 | 3.84E-04 | C | T | N | *TMEM110* |
| rs6431261 | 2 | 235025000 | 0.327 | -0.064 | 3.84E-04 | T | C | N | *ARL4C* |
| rs8053888 | 16 | 52561306 | 0.495 | 0.060 | 3.84E-04 | C | T | Y | *FTO* |
| rs10051652 | 5 | 90975510 | 0.524 | 0.061 | 3.85E-04 | C | T | N | *ARRDC3* |
| rs6712240 | 2 | 235026694 | 0.327 | -0.064 | 3.85E-04 | T | C | N | *ARL4C* |
| rs4783373 | 16 | 20845383 | 0.097 | -0.107 | 3.85E-04 | G | T | N | *LYRM1* |
| rs6585170 | 10 | 114442754 | 0.015 | -0.247 | 3.85E-04 | C | A | Y | *VTI1A* |
| rs6716867 | 2 | 235028086 | 0.327 | -0.064 | 3.85E-04 | T | G | N | *ARL4C* |
| rs1509082 | 2 | 235027424 | 0.327 | -0.064 | 3.85E-04 | T | C | N | *ARL4C* |
| rs2798822 | 14 | 94491274 | 0.391 | -0.062 | 3.85E-04 | T | C | N | *DICER1* |
| rs2349798 | 11 | 26026983 | 0.110 | 0.096 | 3.86E-04 | C | T | N | *TMEM16C* |
| rs9403381 | 6 | 142702849 | 0.294 | 0.066 | 3.86E-04 | T | G | N | *GPR126* |
| rs879638 | 8 | 41770625 | 0.436 | 0.061 | 3.86E-04 | C | T | N | *ANK1* |
| rs1027501 | 8 | 55979084 | 0.329 | 0.064 | 3.87E-04 | A | G | N | *XKR4* |
| rs7785283 | 7 | 150133541 | 0.199 | -0.077 | 3.87E-04 | C | G | N | *TMEM176A* |
| rs17202432 | 10 | 66621754 | 0.394 | 0.062 | 3.87E-04 | T | C | N | *CTNNA3* |
| rs9389986 | 6 | 142702807 | 0.294 | 0.066 | 3.87E-04 | A | T | N | *GPR126* |
| rs2349799 | 11 | 26027049 | 0.110 | 0.096 | 3.88E-04 | T | C | N | *TMEM16C* |
| rs12447590 | 16 | 83571015 | 0.294 | -0.065 | 3.88E-04 | T | C | Y | *ZDHHC7* |
| rs2050157 | 6 | 142699855 | 0.294 | 0.066 | 3.88E-04 | A | G | Y | *GPR126* |
| rs7778162 | 7 | 32281009 | 0.282 | -0.067 | 3.89E-04 | C | T | Y | *PDE1C* |
| rs11772167 | 7 | 150134012 | 0.198 | -0.077 | 3.89E-04 | T | C | N | *TMEM176A* |
| rs13130854 | 4 | 38182092 | 0.310 | 0.066 | 3.89E-04 | G | T | N | *KLF3* |
| rs2036087 | 6 | 170192200 | 0.239 | -0.070 | 3.89E-04 | T | G | N | *DLL1* |
| rs987403 | 10 | 66622862 | 0.394 | 0.062 | 3.89E-04 | C | T | Y | *CTNNA3* |
| rs749316 | 4 | 145590796 | 0.300 | -0.067 | 3.90E-04 | G | A | N | *HHIP* |
| rs7685933 | 4 | 38182664 | 0.310 | 0.066 | 3.90E-04 | T | C | N | *KLF3* |
| rs7073681 | 10 | 114456246 | 0.015 | -0.247 | 3.91E-04 | A | G | Y | *VTI1A* |
| rs2349800 | 11 | 26027081 | 0.110 | 0.096 | 3.91E-04 | A | G | N | *TMEM16C* |
| rs4663417 | 2 | 235031690 | 0.327 | -0.064 | 3.91E-04 | C | G | N | *ARL4C* |
| rs1601670 | 12 | 18821605 | 0.274 | 0.068 | 3.92E-04 | G | T | N | *CAPZA3* |
| rs7906591 | 10 | 114457795 | 0.015 | -0.247 | 3.92E-04 | G | A | Y | *VTI1A* |
| rs1542922 | 18 | 24481864 | 0.264 | 0.069 | 3.93E-04 | G | A | N | *CDH2* |
| rs2078543 | 6 | 22104839 | 0.330 | 0.065 | 3.93E-04 | A | G | N | *PRL* |
| rs6508557 | 18 | 24481039 | 0.264 | 0.069 | 3.93E-04 | C | T | N | *CDH2* |
| rs2497779 | 10 | 18358364 | 0.264 | 0.070 | 3.94E-04 | T | A | N | *SLC39A12* |
| rs9876403 | 3 | 52891956 | 0.232 | 0.071 | 3.95E-04 | T | C | N | *TMEM110* |
| rs13146124 | 4 | 185628402 | 0.241 | -0.071 | 3.97E-04 | A | G | N | *IRF2* |
| rs3179192 | 4 | 185635084 | 0.240 | -0.071 | 3.97E-04 | T | G | N | *IRF2* |
| rs456501 | 10 | 132131097 | 0.248 | 0.071 | 3.98E-04 | G | A | N | *GLRX3* |
| rs12683690 | 9 | 2488874 | 0.226 | 0.072 | 3.98E-04 | A | C | N | *VLDLR* |
| rs7137827 | 12 | 18815008 | 0.274 | 0.068 | 3.98E-04 | G | A | N | *CAPZA3* |
| rs1928168 | 6 | 22125717 | 0.529 | 0.059 | 3.98E-04 | T | C | Y | *PRL* |
| rs2497789 | 10 | 18367737 | 0.277 | 0.067 | 3.99E-04 | T | C | N | *SLC39A12* |
| rs8006086 | 14 | 77843042 | 0.476 | 0.060 | 3.99E-04 | T | C | Y | *NRXN3* |
| rs9865334 | 3 | 80435380 | 0.464 | 0.063 | 4.00E-04 | A | T | N | *ROBO1* |
| rs7335910 | 13 | 24220725 | 0.280 | -0.069 | 4.00E-04 | G | A | N | *RNF17* |
| rs7260196 | 19 | 33696776 | 0.429 | 0.062 | 4.01E-04 | A | G | N | *UQCRFS1* |
| rs10210757 | 2 | 235019803 | 0.327 | -0.063 | 4.01E-04 | C | T | Y | *ARL4C* |
| rs16906885 | 9 | 2488025 | 0.226 | 0.072 | 4.01E-04 | A | G | Y | *VLDLR* |
| rs488965 | 11 | 102133788 | 0.080 | -0.110 | 4.02E-04 | G | A | N | *MMP10* |
| rs372813 | 10 | 132131187 | 0.248 | 0.071 | 4.02E-04 | A | G | N | *GLRX3* |
| rs6046302 | 20 | 19688856 | 0.251 | -0.073 | 4.02E-04 | A | G | N | *SLC24A3* |
| rs2272908 | 1 | 1711339 | 0.502 | 0.060 | 4.02E-04 | C | T | Y | *GNB1* |
| rs9350408 | 6 | 22129352 | 0.468 | 0.060 | 4.02E-04 | C | T | N | *PRL* |
| rs12044597 | 1 | 1698661 | 0.502 | 0.060 | 4.03E-04 | A | G | N | *NADK* |
| rs2935247 | 5 | 91027630 | 0.475 | -0.061 | 4.03E-04 | T | A | N | *ARRDC3* |
| rs507001 | 11 | 102134642 | 0.080 | -0.110 | 4.04E-04 | T | C | Y | *MMP10* |
| rs10841085 | 12 | 18801189 | 0.280 | 0.068 | 4.04E-04 | A | G | N | *CAPZA3* |
| rs12444720 | 16 | 83562534 | 0.293 | -0.068 | 4.05E-04 | C | G | N | *ZDHHC7* |
| rs905959 | 9 | 80369272 | 0.221 | 0.074 | 4.05E-04 | A | T | N | *PSAT1* |
| rs6538982 | 12 | 100232234 | 0.454 | 0.060 | 4.06E-04 | C | T | N | *UTP20* |
| rs4705467 | 5 | 113042475 | 0.145 | -0.087 | 4.06E-04 | G | C | N | *YTHDC2* |
| rs717657 | 7 | 150134924 | 0.198 | -0.077 | 4.07E-04 | T | C | N | *TMEM176A* |
| rs10949749 | 7 | 158448018 | 0.130 | 0.089 | 4.07E-04 | A | G | Y | *WDR60* |
| rs1863316 | 4 | 185627449 | 0.241 | -0.070 | 4.07E-04 | T | A | Y | *IRF2* |
| rs1425555 | 4 | 185640085 | 0.240 | -0.071 | 4.09E-04 | C | T | N | *IRF2* |
| rs2349801 | 11 | 26027213 | 0.110 | 0.095 | 4.10E-04 | G | C | N | *TMEM16C* |
| rs1375460 | 5 | 120093645 | 0.316 | -0.067 | 4.10E-04 | G | A | N | *PRR16* |
| rs11699714 | 20 | 1561011 | 0.050 | 0.142 | 4.11E-04 | C | T | N | *SIRPG* |
| rs848042 | 14 | 36405211 | 0.422 | 0.063 | 4.13E-04 | C | T | N | *SLC25A21* |
| rs2973826 | 5 | 91017288 | 0.476 | -0.061 | 4.13E-04 | T | G | N | *ARRDC3* |
| rs13402680 | 2 | 235019147 | 0.327 | -0.064 | 4.13E-04 | G | C | N | *ARL4C* |
| rs7835438 | 8 | 41781413 | 0.422 | 0.061 | 4.13E-04 | G | C | N | *ANK1* |
| rs8089892 | 18 | 33262821 | 0.324 | -0.064 | 4.15E-04 | C | T | Y | *BRUNOL4* |
| rs10914863 | 1 | 34344752 | 0.418 | -0.061 | 4.15E-04 | T | A | N | *CSMD2* |
| rs10431196 | 11 | 51311887 | 0.176 | -0.080 | 4.19E-04 | G | C | N | *OR4A5* |
| rs6567281 | 18 | 58225849 | 0.241 | -0.073 | 4.20E-04 | C | A | N | *TNFRSF11A* |
| rs10933290 | 2 | 229926510 | 0.409 | 0.062 | 4.20E-04 | A | G | N | *DNER* |
| rs13264181 | 8 | 41780689 | 0.422 | 0.061 | 4.20E-04 | G | A | N | *ANK1* |
| rs2481712 | 1 | 93124065 | 0.373 | 0.061 | 4.20E-04 | G | A | N | *RPL5* |
| rs573553 | 3 | 174686959 | 0.107 | -0.100 | 4.21E-04 | T | G | N | *NLGN1* |
| rs7540201 | 1 | 97632909 | 0.235 | -0.071 | 4.21E-04 | A | C | N | *DPYD* |
| rs9940177 | 16 | 13171686 | 0.442 | 0.060 | 4.22E-04 | G | A | Y | *FLJ11151* |
| rs7797919 | 7 | 51555079 | 0.209 | 0.076 | 4.24E-04 | C | T | N | *COBL* |
| rs248432 | 5 | 150544558 | 0.503 | 0.061 | 4.24E-04 | G | C | Y | *CCDC69* |
| rs10742129 | 11 | 26028038 | 0.110 | 0.095 | 4.25E-04 | T | G | N | *TMEM16C* |
| rs10980114 | 9 | 111713324 | 0.380 | 0.062 | 4.25E-04 | G | A | N | *PALM2* |
| rs12308038 | 12 | 18904530 | 0.109 | 0.096 | 4.26E-04 | C | G | N | *CAPZA3* |
| rs11054761 | 12 | 12321303 | 0.061 | -0.125 | 4.26E-04 | C | T | Y | *LRP6* |
| rs10841083 | 12 | 18798277 | 0.282 | 0.067 | 4.27E-04 | C | T | Y | *CAPZA3* |
| rs1864759 | 10 | 62473595 | 0.037 | 0.159 | 4.27E-04 | C | T | Y | *RHOBTB1* |
| rs11768207 | 7 | 32293832 | 0.281 | -0.067 | 4.28E-04 | C | G | Y | *LSM5* |
| rs12039526 | 1 | 230339222 | 0.458 | 0.060 | 4.29E-04 | C | T | N | *DISC1* |
| rs12411169 | 1 | 97634092 | 0.235 | -0.071 | 4.30E-04 | A | C | Y | *DPYD* |
| rs7897808 | 10 | 32456613 | 0.352 | 0.062 | 4.30E-04 | C | T | N | *KIF5B* |
| rs1387482 | 11 | 26030956 | 0.110 | 0.095 | 4.30E-04 | C | G | N | *TMEM16C* |
| rs12566907 | 1 | 97634825 | 0.235 | -0.071 | 4.30E-04 | C | T | N | *DPYD* |
| rs6990353 | 8 | 6160762 | 0.129 | -0.091 | 4.31E-04 | C | G | Y | *MCPH1* |
| rs4723832 | 7 | 39217984 | 0.334 | 0.064 | 4.31E-04 | T | C | Y | *POU6F2* |
| rs17692345 | 16 | 20823904 | 0.097 | -0.106 | 4.32E-04 | A | C | N | *LYRM1* |
| rs964624 | 11 | 26032292 | 0.110 | 0.095 | 4.32E-04 | A | G | N | *TMEM16C* |
| rs12889327 | 14 | 94492966 | 0.376 | -0.062 | 4.33E-04 | T | C | N | *DICER1* |
| rs4370439 | 7 | 32274626 | 0.282 | -0.066 | 4.33E-04 | C | T | N | *PDE1C* |
| rs12881250 | 14 | 94495464 | 0.376 | -0.062 | 4.33E-04 | C | A | N | *DICER1* |
| rs17691515 | 16 | 20768573 | 0.103 | -0.100 | 4.34E-04 | A | C | N | *LOC81691* |
| rs10864720 | 1 | 230337855 | 0.458 | 0.059 | 4.34E-04 | C | T | Y | *DISC1* |
| rs11070518 | 15 | 44489497 | 0.321 | 0.065 | 4.34E-04 | C | T | N | *SQRDL* |
| rs290193 | 11 | 85106722 | 0.457 | 0.061 | 4.35E-04 | A | G | N | *SYTL2* |
| rs1791581 | 11 | 106001248 | 0.166 | 0.083 | 4.35E-04 | A | G | N | *GUCY1A2* |
| rs8094965 | 18 | 36896753 | 0.041 | -0.151 | 4.36E-04 | T | C | N | *PIK3C3* |
| rs964625 | 11 | 26032369 | 0.110 | 0.095 | 4.36E-04 | C | T | N | *TMEM16C* |
| rs6954406 | 7 | 39243641 | 0.434 | -0.061 | 4.37E-04 | G | C | N | *POU6F2* |
| rs225211 | 17 | 27919415 | 0.395 | 0.061 | 4.38E-04 | G | C | Y | *MYO1D* |
| rs1995533 | 8 | 55412476 | 0.248 | -0.072 | 4.41E-04 | A | G | N | *SOX17* |
| rs2774949 | 1 | 93125141 | 0.372 | 0.061 | 4.41E-04 | T | C | N | *RPL5* |
| rs7517158 | 1 | 153353517 | 0.073 | -0.118 | 4.41E-04 | C | T | N | *EFNA1* |
| rs9855082 | 3 | 80442545 | 0.476 | 0.060 | 4.42E-04 | A | G | Y | *ROBO1* |
| rs7233276 | 18 | 36894927 | 0.041 | -0.151 | 4.42E-04 | T | C | N | *PIK3C3* |
| rs11054760 | 12 | 12320749 | 0.061 | -0.125 | 4.42E-04 | A | G | Y | *LRP6* |
| rs7238667 | 18 | 36894703 | 0.041 | -0.151 | 4.42E-04 | T | C | Y | *PIK3C3* |
| rs7670758 | 4 | 145731325 | 0.441 | -0.060 | 4.42E-04 | A | G | Y | *HHIP* |
| rs2130231 | 11 | 26032788 | 0.110 | 0.094 | 4.43E-04 | A | T | N | *TMEM16C* |
| rs2811593 | 1 | 93116479 | 0.372 | 0.061 | 4.43E-04 | T | C | N | *RPL5* |
| rs696284 | 9 | 33192548 | 0.329 | 0.063 | 4.43E-04 | G | T | Y | *B4GALT1* |
| rs9892875 | 17 | 58557163 | 0.467 | 0.060 | 4.43E-04 | A | C | N | *TANC2* |
| rs1888601 | 1 | 230333545 | 0.458 | 0.059 | 4.43E-04 | C | T | N | *DISC1* |
| rs16974026 | 18 | 36894108 | 0.041 | -0.151 | 4.44E-04 | C | T | N | *PIK3C3* |
| rs7178469 | 15 | 50911012 | 0.040 | -0.156 | 4.44E-04 | C | T | N | *ONECUT1* |
| rs867844 | 1 | 230332901 | 0.458 | 0.059 | 4.45E-04 | T | A | Y | *DISC1* |
| rs11605090 | 11 | 74301879 | 0.066 | 0.121 | 4.45E-04 | C | G | N | *XRRA1* |
| rs6055570 | 20 | 8077324 | 0.402 | 0.062 | 4.46E-04 | A | C | N | *PLCB1* |
| rs2300230 | 12 | 12207124 | 0.077 | -0.112 | 4.47E-04 | C | T | N | *LRP6* |
| rs10271037 | 7 | 32296861 | 0.281 | -0.066 | 4.47E-04 | T | G | Y | *LSM5* |
| rs4674187 | 2 | 218108860 | 0.102 | -0.099 | 4.47E-04 | G | C | Y | *TNS1* |
| rs1489501 | 11 | 26039527 | 0.111 | 0.094 | 4.48E-04 | G | C | N | *TMEM16C* |
| rs7973115 | 12 | 93914941 | 0.259 | -0.069 | 4.48E-04 | T | G | N | *NDUFA12* |
| rs11607203 | 11 | 74308145 | 0.066 | 0.121 | 4.48E-04 | G | T | N | *XRRA1* |
| rs9874140 | 3 | 80421052 | 0.481 | 0.061 | 4.50E-04 | A | G | N | *ROBO1* |
| rs11604806 | 11 | 74370296 | 0.066 | 0.121 | 4.50E-04 | G | A | N | *SPCS2* |
| rs10501035 | 11 | 26017220 | 0.138 | 0.085 | 4.50E-04 | A | T | Y | *TMEM16C* |
| rs10501034 | 11 | 26015770 | 0.138 | 0.085 | 4.51E-04 | C | G | Y | *TMEM16C* |
| rs4328171 | 11 | 26016429 | 0.138 | 0.085 | 4.51E-04 | T | C | Y | *TMEM16C* |
| rs2041516 | 7 | 31910256 | 0.193 | 0.076 | 4.52E-04 | C | G | N | *PDE1C* |
| rs290198 | 11 | 85097315 | 0.459 | 0.062 | 4.52E-04 | C | T | N | *SYTL2* |
| rs13238655 | 7 | 48750616 | 0.069 | -0.119 | 4.53E-04 | C | T | N | *ABCA13* |
| rs4765525 | 12 | 126323618 | 0.139 | -0.086 | 4.53E-04 | A | G | Y | *SLC15A4* |
| rs1489502 | 11 | 26039583 | 0.111 | 0.094 | 4.54E-04 | C | T | Y | *TMEM16C* |
| rs10994618 | 10 | 62468415 | 0.037 | 0.159 | 4.55E-04 | A | C | N | *RHOBTB1* |
| rs2774953 | 1 | 93127842 | 0.372 | 0.061 | 4.55E-04 | G | A | N | *RPL5* |
| rs9871716 | 3 | 80415831 | 0.481 | 0.060 | 4.55E-04 | A | G | N | *ROBO1* |
| rs10501629 | 11 | 86137614 | 0.129 | -0.090 | 4.56E-04 | T | A | N | *PRSS23* |
| rs4925363 | 20 | 60269044 | 0.044 | -0.144 | 4.56E-04 | C | G | Y | *OSBPL2* |
| rs741064 | 7 | 150131662 | 0.199 | -0.075 | 4.57E-04 | A | G | Y | *TMEM176A* |
| rs6580470 | 5 | 146901671 | 0.173 | 0.078 | 4.58E-04 | C | G | N | *JAKMIP2* |
| rs2481710 | 1 | 93110082 | 0.372 | 0.061 | 4.59E-04 | T | C | N | *RPL5* |
| rs12109947 | 5 | 146897476 | 0.173 | 0.078 | 4.60E-04 | T | C | N | *JAKMIP2* |
| rs2255723 | 1 | 93140897 | 0.372 | 0.061 | 4.60E-04 | G | T | Y | *FAM69A* |
| rs12108716 | 5 | 146897411 | 0.173 | 0.078 | 4.60E-04 | G | A | N | *JAKMIP2* |
| rs3006640 | 10 | 32457808 | 0.352 | 0.061 | 4.61E-04 | G | A | N | *KIF5B* |
| rs7715946 | 5 | 146905560 | 0.173 | 0.078 | 4.62E-04 | G | A | Y | *JAKMIP2* |
| rs16974040 | 18 | 36903928 | 0.041 | -0.152 | 4.62E-04 | C | T | N | *PIK3C3* |
| rs16974011 | 18 | 36888680 | 0.041 | -0.152 | 4.62E-04 | G | A | N | *PIK3C3* |
| rs6481776 | 10 | 32458311 | 0.352 | 0.061 | 4.62E-04 | T | C | N | *KIF5B* |
| rs11953038 | 5 | 146896436 | 0.173 | 0.078 | 4.65E-04 | C | T | N | *JAKMIP2* |
| rs1994547 | 11 | 26039946 | 0.110 | 0.094 | 4.65E-04 | T | A | Y | *TMEM16C* |
| rs7789925 | 7 | 39231954 | 0.335 | 0.063 | 4.65E-04 | A | G | N | *POU6F2* |
| rs1994548 | 11 | 26040006 | 0.110 | 0.094 | 4.65E-04 | A | G | N | *TMEM16C* |
| rs8090050 | 18 | 36902291 | 0.041 | -0.152 | 4.65E-04 | C | T | N | *PIK3C3* |
| rs1994549 | 11 | 26040028 | 0.110 | 0.094 | 4.66E-04 | T | G | Y | *TMEM16C* |
| rs7097979 | 10 | 32458480 | 0.352 | 0.061 | 4.66E-04 | T | C | Y | *KIF5B* |
| rs331387 | 5 | 91036123 | 0.473 | -0.060 | 4.66E-04 | G | A | N | *ARRDC3* |
| rs331393 | 5 | 91044514 | 0.474 | -0.060 | 4.66E-04 | T | G | N | *ARRDC3* |
| rs331392 | 5 | 91044828 | 0.474 | -0.060 | 4.67E-04 | A | G | N | *ARRDC3* |
| rs12807406 | 11 | 77358047 | 0.256 | 0.070 | 4.67E-04 | T | G | N | *INTS4* |
| rs1653876 | 7 | 32327144 | 0.258 | -0.068 | 4.68E-04 | T | C | N | *LSM5* |
| rs331390 | 5 | 91045508 | 0.474 | -0.060 | 4.69E-04 | A | G | N | *ARRDC3* |
| rs2811600 | 1 | 93106726 | 0.371 | 0.061 | 4.70E-04 | C | T | Y | *RPL5* |
| rs717191 | 22 | 24972679 | 0.180 | 0.078 | 4.70E-04 | A | G | N | *SEZ6L* |
| rs1432846 | 5 | 146893468 | 0.173 | 0.078 | 4.70E-04 | T | C | N | *JAKMIP2* |
| rs975122 | 7 | 32269319 | 0.280 | -0.066 | 4.70E-04 | A | T | N | *PDE1C* |
| rs12819916 | 12 | 12178856 | 0.077 | -0.112 | 4.70E-04 | T | C | N | *LRP6* |
| rs1885284 | 20 | 21418454 | 0.402 | -0.060 | 4.71E-04 | C | T | N | *NKX2-2* |
| rs239494 | 6 | 80756460 | 0.471 | 0.059 | 4.71E-04 | C | A | N | *TTK* |
| rs7716144 | 5 | 146892300 | 0.173 | 0.078 | 4.71E-04 | C | T | N | *JAKMIP2* |
| rs17650890 | 5 | 146891632 | 0.173 | 0.078 | 4.72E-04 | A | G | N | *JAKMIP2* |
| rs151646 | 6 | 80756426 | 0.471 | 0.059 | 4.72E-04 | T | A | Y | *TTK* |
| rs3743562 | 16 | 56610662 | 0.207 | -0.073 | 4.73E-04 | C | T | Y | *C16orf57* |
| rs3886205 | 22 | 24972934 | 0.180 | 0.078 | 4.73E-04 | A | G | N | *SEZ6L* |
| rs10900284 | 10 | 42702598 | 0.202 | -0.076 | 4.73E-04 | A | T | Y | *BMS1* |
| rs2657816 | 5 | 91053186 | 0.474 | -0.060 | 4.74E-04 | G | C | N | *ARRDC3* |
| rs6597033 | 6 | 4115527 | 0.130 | 0.089 | 4.74E-04 | C | A | Y | *PECI* |
| rs2561063 | 5 | 91053281 | 0.474 | -0.060 | 4.74E-04 | T | C | N | *ARRDC3* |
| rs12819810 | 12 | 12178280 | 0.077 | -0.112 | 4.75E-04 | A | T | N | *LRP6* |
| rs1391468 | 8 | 55975683 | 0.330 | 0.063 | 4.75E-04 | G | A | N | *XKR4* |
| rs2929649 | 15 | 36829662 | 0.307 | -0.065 | 4.75E-04 | A | C | N | *FLJ35695* |
| rs3803651 | 16 | 79876383 | 0.283 | -0.069 | 4.75E-04 | G | A | N | *BCMO1* |
| rs12449108 | 16 | 79876939 | 0.283 | -0.069 | 4.75E-04 | A | G | N | *BCMO1* |
| rs1012672 | 12 | 12176182 | 0.077 | -0.112 | 4.76E-04 | A | G | N | *LRP6* |
| rs2929648 | 15 | 36829105 | 0.307 | -0.065 | 4.76E-04 | G | A | N | *FLJ35695* |
| rs4765524 | 12 | 126322887 | 0.139 | -0.086 | 4.77E-04 | T | C | N | *SLC15A4* |
| rs1009090 | 12 | 100227382 | 0.455 | 0.059 | 4.78E-04 | A | G | Y | *UTP20* |
| rs7143051 | 14 | 56624529 | 0.308 | 0.067 | 4.78E-04 | C | T | N | *EXOC5* |
| rs11752585 | 6 | 4114694 | 0.130 | 0.089 | 4.78E-04 | C | T | N | *PECI* |
| rs11699591 | 20 | 1553165 | 0.059 | 0.126 | 4.79E-04 | A | G | N | *SIRPB1* |
| rs11120299 | 1 | 212576030 | 0.331 | -0.063 | 4.79E-04 | A | C | N | *SMYD2* |
| rs12317211 | 12 | 100228652 | 0.455 | 0.059 | 4.79E-04 | T | C | N | *UTP20* |
| rs6538981 | 12 | 100226636 | 0.455 | 0.059 | 4.80E-04 | C | T | N | *UTP20* |
| rs1048791 | 1 | 212578142 | 0.331 | -0.063 | 4.80E-04 | C | T | N | *SMYD2* |
| rs4766926 | 12 | 118025078 | 0.331 | -0.065 | 4.81E-04 | A | T | N | *KIAA1853* |
| rs13197025 | 6 | 4116417 | 0.121 | 0.094 | 4.83E-04 | A | G | N | *PECI* |
| rs2172585 | 8 | 55975375 | 0.330 | 0.063 | 4.85E-04 | A | T | N | *XKR4* |
| rs698640 | 7 | 56218441 | 0.163 | 0.080 | 4.86E-04 | C | G | Y | *CHCHD2* |
| rs789557 | 11 | 131109572 | 0.190 | 0.076 | 4.86E-04 | G | C | Y | *HNT* |
| rs17114161 | 10 | 90670204 | 0.030 | 0.173 | 4.86E-04 | C | G | Y | *STAMBPL1* |
| rs6538980 | 12 | 100217097 | 0.455 | 0.059 | 4.86E-04 | G | A | Y | *UTP20* |
| rs2520490 | 4 | 150130919 | 0.399 | 0.061 | 4.87E-04 | A | G | N | *NR3C2* |
| rs4426587 | 20 | 21421495 | 0.401 | -0.060 | 4.87E-04 | G | A | N | *NKX2-2* |
| rs7751774 | 6 | 4111577 | 0.130 | 0.089 | 4.87E-04 | C | T | N | *PECI* |
| rs4750540 | 10 | 14703390 | 0.111 | 0.094 | 4.87E-04 | A | G | Y | *FAM107B* |
| rs6137363 | 20 | 21423961 | 0.401 | -0.060 | 4.88E-04 | G | C | N | *NKX2-2* |
| rs11680888 | 2 | 239572546 | 0.181 | 0.080 | 4.89E-04 | C | G | N | *HDAC4* |
| rs2255717 | 1 | 93141088 | 0.373 | 0.061 | 4.91E-04 | G | T | N | *FAM69A* |
| rs3105607 | 11 | 131107765 | 0.189 | 0.076 | 4.92E-04 | A | C | N | *HNT* |
| rs10242061 | 7 | 39260567 | 0.442 | 0.060 | 4.93E-04 | G | A | Y | *POU6F2* |
| rs1506670 | 11 | 131106975 | 0.189 | 0.076 | 4.94E-04 | A | T | N | *HNT* |
| rs6857191 | 4 | 150141106 | 0.399 | 0.061 | 4.94E-04 | T | A | N | *NR3C2* |
| rs7112290 | 11 | 51334648 | 0.162 | -0.081 | 4.94E-04 | A | G | N | *OR4C46* |
| rs9532580 | 13 | 40142260 | 0.272 | 0.067 | 4.95E-04 | C | T | N | *FOXO1* |
| rs2002922 | 6 | 4110364 | 0.130 | 0.089 | 4.95E-04 | A | G | N | *PECI* |
| rs3789643 | 1 | 228876057 | 0.012 | -0.273 | 4.95E-04 | G | T | N | *COG2* |
| rs9655034 | 7 | 39258636 | 0.442 | 0.060 | 4.95E-04 | T | G | N | *POU6F2* |
| rs10116004 | 9 | 72336602 | 0.556 | 0.060 | 4.96E-04 | C | T | Y | *TRPM3* |
| rs1149997 | 10 | 9395973 | 0.423 | -0.059 | 4.96E-04 | C | T | Y | *GATA3* |
| rs11068246 | 12 | 115858114 | 0.012 | -0.268 | 4.96E-04 | A | G | N | *FBXW8* |
| rs16947131 | 12 | 115856149 | 0.012 | -0.268 | 4.96E-04 | C | T | N | *FBXW8* |
| rs16947122 | 12 | 115850914 | 0.012 | -0.268 | 4.96E-04 | T | C | Y | *FBXW8* |
| rs2531853 | 17 | 9143979 | 0.445 | -0.059 | 4.96E-04 | T | G | Y | *STX8* |
| rs4335493 | 11 | 26017594 | 0.138 | 0.085 | 4.96E-04 | T | G | N | *TMEM16C* |
| rs1761769 | 6 | 43787025 | 0.436 | 0.060 | 4.97E-04 | G | T | N | *MRPS18A* |
| rs949915 | 1 | 93142362 | 0.373 | 0.060 | 4.98E-04 | A | G | N | *FAM69A* |
| rs2520492 | 4 | 150144815 | 0.399 | 0.061 | 4.98E-04 | A | G | N | *NR3C2* |
| rs2277027 | 5 | 156864954 | 0.348 | -0.062 | 4.99E-04 | C | A | N | *ADAM19* |
| rs10078178 | 5 | 156865562 | 0.348 | -0.062 | 4.99E-04 | T | A | N | *ADAM19* |
| rs4856611 | 3 | 80463192 | 0.257 | -0.068 | 4.99E-04 | G | A | N | *ROBO1* |
| rs789531 | 11 | 131063678 | 0.192 | 0.077 | 4.99E-04 | T | C | N | *HNT* |
| rs1868017 | 12 | 126322523 | 0.140 | -0.086 | 4.99E-04 | C | T | N | *SLC15A4* |
| rs6938649 | 6 | 4130318 | 0.131 | 0.088 | 5.00E-04 | T | C | N | *PECI* |
| rs4236356 | 7 | 39234720 | 0.338 | 0.063 | 5.00E-04 | G | T | N | *POU6F2* |
| rs10887971 | 21 | 42062915 | 0.271 | -0.069 | 5.00E-04 | A | G | N | *RIPK4* |
| rs1150003 | 10 | 9396841 | 0.425 | -0.060 | 5.00E-04 | C | G | N | *GATA3* |
| rs6912707 | 6 | 4108636 | 0.130 | 0.089 | 5.01E-04 | T | C | N | *PECI* |
| rs1868018 | 12 | 126322571 | 0.139 | -0.086 | 5.01E-04 | T | C | N | *SLC15A4* |
| rs2068592 | 7 | 70494069 | 0.017 | -0.230 | 5.01E-04 | A | G | N | *WBSCR17* |
| rs1509051 | 12 | 126321993 | 0.140 | -0.086 | 5.02E-04 | C | T | N | *SLC15A4* |
| rs1351013 | 11 | 96965416 | 0.064 | -0.125 | 5.02E-04 | T | C | N | *JRKL* |
| rs12494389 | 3 | 80461655 | 0.257 | -0.068 | 5.02E-04 | T | A | N | *ROBO1* |
| rs1422795 | 5 | 156868942 | 0.348 | -0.062 | 5.03E-04 | C | T | Y | *ADAM19* |
| rs11134779 | 5 | 156869344 | 0.348 | -0.062 | 5.03E-04 | G | A | Y | *ADAM19* |
| rs1432076 | 18 | 68424021 | 0.098 | -0.099 | 5.04E-04 | A | G | N | *CBLN2* |
| rs10866659 | 5 | 156869621 | 0.348 | -0.062 | 5.04E-04 | G | A | N | *ADAM19* |
| rs6912138 | 6 | 4108302 | 0.130 | 0.089 | 5.05E-04 | T | C | N | *PECI* |
| rs13224417 | 7 | 32265118 | 0.282 | -0.066 | 5.06E-04 | A | G | N | *PDE1C* |
| rs10478138 | 5 | 113049912 | 0.140 | -0.084 | 5.06E-04 | G | T | Y | *YTHDC2* |
| rs654804 | 13 | 52299506 | 0.429 | -0.062 | 5.07E-04 | A | G | N | *PCDH8* |
| rs7141979 | 14 | 53533113 | 0.152 | -0.082 | 5.08E-04 | G | A | N | *BMP4* |
| rs1432075 | 18 | 68424058 | 0.098 | -0.099 | 5.08E-04 | T | C | N | *CBLN2* |
| rs4579242 | 5 | 156872076 | 0.348 | -0.062 | 5.08E-04 | G | T | Y | *ADAM19* |
| rs12830315 | 12 | 80152366 | 0.018 | 0.225 | 5.08E-04 | A | G | N | *FLJ21963* |
| rs816394 | 7 | 56221908 | 0.164 | 0.079 | 5.09E-04 | C | G | N | *CHCHD2* |
| rs2416329 | 5 | 113051018 | 0.138 | -0.085 | 5.09E-04 | T | G | Y | *YTHDC2* |
| rs7548383 | 1 | 153353603 | 0.068 | -0.117 | 5.10E-04 | T | C | Y | *EFNA1* |
| rs9439948 | 1 | 93143388 | 0.373 | 0.060 | 5.10E-04 | A | G | N | *FAM69A* |
| rs13221985 | 7 | 32259510 | 0.281 | -0.066 | 5.11E-04 | A | C | N | *PDE1C* |
| rs6948856 | 7 | 32268872 | 0.292 | -0.067 | 5.11E-04 | A | G | N | *PDE1C* |
| rs4587687 | 11 | 26017650 | 0.138 | 0.085 | 5.11E-04 | A | G | N | *TMEM16C* |
| rs9956893 | 18 | 68425966 | 0.098 | -0.099 | 5.11E-04 | A | G | N | *CBLN2* |
| rs10496708 | 2 | 134153826 | 0.026 | 0.186 | 5.11E-04 | T | C | Y | *NAP5* |
| rs11762194 | 7 | 32266694 | 0.282 | -0.066 | 5.11E-04 | A | G | N | *PDE1C* |
| rs2887830 | 10 | 32429430 | 0.472 | 0.058 | 5.13E-04 | T | C | N | *KIF5B* |
| rs2295841 | 6 | 80806812 | 0.324 | -0.065 | 5.13E-04 | G | C | N | *TTK* |
| rs4336988 | 11 | 26017740 | 0.138 | 0.084 | 5.14E-04 | T | C | N | *TMEM16C* |
| rs2181070 | 6 | 80805498 | 0.324 | -0.065 | 5.15E-04 | G | A | N | *TTK* |
| rs8034728 | 15 | 50652177 | 0.096 | 0.100 | 5.15E-04 | C | A | Y | *ARPP-19* |
| rs720084 | 5 | 58075398 | 0.134 | -0.090 | 5.15E-04 | G | A | N | *RAB3C* |
| rs4737754 | 8 | 55957862 | 0.330 | 0.062 | 5.15E-04 | A | G | N | *XKR4* |
| rs11773343 | 7 | 32258841 | 0.281 | -0.066 | 5.15E-04 | T | C | N | *PDE1C* |
| rs248431 | 5 | 150544597 | 0.506 | 0.062 | 5.16E-04 | G | A | N | *CCDC69* |
| rs4353231 | 11 | 26017811 | 0.138 | 0.084 | 5.18E-04 | G | A | N | *TMEM16C* |
| rs9844736 | 3 | 52907011 | 0.235 | 0.069 | 5.18E-04 | T | G | N | *TMEM110* |
| rs11949718 | 5 | 146890408 | 0.173 | 0.077 | 5.18E-04 | A | G | N | *JAKMIP2* |
| rs9643483 | 8 | 55960228 | 0.330 | 0.062 | 5.19E-04 | G | T | Y | *XKR4* |
| rs7194907 | 16 | 52560984 | 0.495 | 0.059 | 5.19E-04 | C | T | Y | *FTO* |
| rs7217278 | 17 | 65438031 | 0.071 | -0.116 | 5.19E-04 | G | A | N | *KCNJ16* |
| rs7517162 | 1 | 153353529 | 0.068 | -0.117 | 5.19E-04 | G | T | N | *EFNA1* |
| rs698629 | 7 | 56228170 | 0.165 | 0.079 | 5.20E-04 | A | T | N | *CHCHD2* |
| rs7789406 | 7 | 39254508 | 0.442 | 0.060 | 5.20E-04 | A | G | N | *POU6F2* |
| rs17036186 | 2 | 68965540 | 0.048 | -0.141 | 5.21E-04 | G | A | N | *BMP10* |
| rs11812364 | 10 | 14696190 | 0.111 | 0.096 | 5.21E-04 | A | G | N | *FAM107B* |
| rs13029129 | 2 | 239578942 | 0.179 | 0.080 | 5.21E-04 | A | G | N | *HDAC4* |
| rs1134546 | 3 | 52912814 | 0.235 | 0.069 | 5.21E-04 | G | A | N | *SFMBT1* |
| rs4535064 | 2 | 239576302 | 0.179 | 0.079 | 5.22E-04 | G | A | N | *HDAC4* |
| rs2066486 | 9 | 98046399 | 0.273 | -0.066 | 5.22E-04 | C | T | N | *HSD17B3* |
| rs17106850 | 5 | 146886959 | 0.173 | 0.077 | 5.22E-04 | T | C | Y | *JAKMIP2* |
| rs6544241 | 2 | 22916202 | 0.441 | 0.059 | 5.23E-04 | A | G | N | *ATAD2B* |
| rs2130234 | 11 | 26018016 | 0.138 | 0.084 | 5.23E-04 | A | C | N | *TMEM16C* |
| rs789245 | 3 | 130066270 | 0.232 | -0.071 | 5.23E-04 | A | C | N | *ACAD9* |
| rs12055156 | 5 | 146886180 | 0.173 | 0.077 | 5.23E-04 | G | C | N | *JAKMIP2* |
| rs7824078 | 8 | 55966296 | 0.330 | 0.062 | 5.24E-04 | A | G | N | *XKR4* |
| rs2415116 | 15 | 69460239 | 0.201 | -0.074 | 5.24E-04 | T | C | N | *LOC645296* |
| rs6681346 | 1 | 228877379 | 0.012 | -0.271 | 5.24E-04 | A | T | Y | *COG2* |
| rs789247 | 3 | 130063275 | 0.232 | -0.071 | 5.24E-04 | A | C | N | *ACAD9* |
| rs881004 | 11 | 131110378 | 0.189 | 0.075 | 5.25E-04 | G | A | N | *HNT* |
| rs17253452 | 14 | 53531911 | 0.152 | -0.082 | 5.25E-04 | C | A | N | *BMP4* |
| rs8190541 | 9 | 98053437 | 0.285 | -0.067 | 5.26E-04 | T | C | N | *HSD17B3* |
| rs9348290 | 6 | 170183096 | 0.246 | -0.069 | 5.26E-04 | T | C | N | *DLL1* |
| rs7293187 | 22 | 16812720 | 0.025 | 0.190 | 5.26E-04 | G | C | Y | *MICAL3* |
| rs12066638 | 1 | 93147979 | 0.373 | 0.060 | 5.26E-04 | G | C | N | *FAM69A* |
| rs12321592 | 12 | 115849213 | 0.012 | -0.267 | 5.28E-04 | A | G | N | *FBXW8* |
| rs9895280 | 17 | 358119 | 0.015 | 0.242 | 5.28E-04 | G | A | Y | *VPS53* |
| rs7178036 | 15 | 50911300 | 0.040 | -0.153 | 5.28E-04 | T | G | N | *ONECUT1* |
| rs6572825 | 14 | 51705382 | 0.024 | 0.192 | 5.29E-04 | G | A | N | *C14orf166* |
| rs729390 | 9 | 98052349 | 0.273 | -0.067 | 5.29E-04 | T | C | N | *HSD17B3* |
| rs2171734 | 11 | 26018381 | 0.138 | 0.084 | 5.29E-04 | C | T | N | *TMEM16C* |
| rs12156604 | 9 | 7864458 | 0.463 | -0.061 | 5.29E-04 | G | A | N | *C9orf123* |
| rs2476921 | 9 | 98049774 | 0.273 | -0.066 | 5.29E-04 | C | T | N | *HSD17B3* |
| rs371119 | 9 | 98050673 | 0.273 | -0.067 | 5.29E-04 | C | T | N | *HSD17B3* |
| rs10939557 | 4 | 14114650 | 0.446 | 0.059 | 5.30E-04 | T | C | N | *CPEB2* |
| rs12316811 | 12 | 115848948 | 0.012 | -0.267 | 5.30E-04 | A | T | N | *FBXW8* |
| rs6124123 | 20 | 37282921 | 0.268 | 0.069 | 5.30E-04 | A | T | N | *DHX35* |
| rs13178544 | 5 | 113049864 | 0.140 | -0.084 | 5.30E-04 | G | A | N | *YTHDC2* |
| rs407179 | 9 | 98052098 | 0.273 | -0.067 | 5.31E-04 | T | C | N | *HSD17B3* |
| rs867807 | 9 | 98053071 | 0.273 | -0.067 | 5.31E-04 | C | T | N | *HSD17B3* |
| rs13126633 | 4 | 14112282 | 0.446 | 0.059 | 5.32E-04 | C | T | N | *CPEB2* |
| rs11623284 | 14 | 51702368 | 0.024 | 0.192 | 5.32E-04 | G | A | N | *C14orf166* |
| rs1032285 | 11 | 26013730 | 0.139 | 0.084 | 5.33E-04 | C | T | N | *TMEM16C* |
| rs4704298 | 5 | 75549821 | 0.284 | 0.065 | 5.33E-04 | T | C | Y | *SV2C* |
| rs10272803 | 7 | 39252328 | 0.442 | 0.060 | 5.33E-04 | G | T | N | *POU6F2* |
| rs7926366 | 11 | 26018424 | 0.138 | 0.084 | 5.35E-04 | C | T | N | *TMEM16C* |
| rs7157602 | 14 | 94490393 | 0.375 | -0.061 | 5.35E-04 | A | T | Y | *DICER1* |
| rs8042899 | 15 | 50671196 | 0.091 | 0.105 | 5.36E-04 | T | G | N | *KIAA1370* |
| rs12127760 | 1 | 212578929 | 0.111 | 0.094 | 5.37E-04 | A | G | N | *SMYD2* |
| rs1928161 | 6 | 22104340 | 0.319 | 0.063 | 5.37E-04 | T | C | Y | *PRL* |
| rs7963012 | 12 | 115847052 | 0.012 | -0.267 | 5.37E-04 | T | C | N | *FBXW8* |
| rs16974638 | 17 | 65443022 | 0.071 | -0.116 | 5.38E-04 | C | T | N | *KCNJ16* |
| rs6439885 | 3 | 141103087 | 0.173 | -0.078 | 5.38E-04 | A | C | Y | *CLSTN2* |
| rs7301012 | 12 | 12198911 | 0.176 | -0.078 | 5.38E-04 | A | G | N | *LRP6* |
| rs7929362 | 11 | 26018594 | 0.138 | 0.084 | 5.39E-04 | C | T | N | *TMEM16C* |
| rs1410523 | 6 | 22104717 | 0.319 | 0.063 | 5.39E-04 | T | C | N | *PRL* |
| rs10994616 | 10 | 62455952 | 0.037 | 0.158 | 5.40E-04 | T | C | N | *RHOBTB1* |
| rs867393 | 12 | 61564734 | 0.483 | 0.059 | 5.41E-04 | T | C | Y | *PPM1H* |
| rs17036192 | 2 | 68968567 | 0.048 | -0.141 | 5.41E-04 | A | G | N | *BMP10* |
| rs168595 | 10 | 132137577 | 0.238 | 0.070 | 5.41E-04 | G | T | N | *GLRX3* |
| rs3094672 | 6 | 31101356 | 0.239 | 0.068 | 5.42E-04 | T | A | Y | *C6orf205* |
| rs8071899 | 17 | 65442541 | 0.071 | -0.115 | 5.42E-04 | G | A | N | *KCNJ16* |
| rs9995614 | 4 | 11244772 | 0.398 | 0.059 | 5.43E-04 | C | T | Y | *HS3ST1* |
| rs11723125 | 4 | 11252282 | 0.411 | 0.060 | 5.43E-04 | G | A | N | *HS3ST1* |
| rs1576618 | 1 | 230310954 | 0.440 | 0.059 | 5.43E-04 | T | C | Y | *DISC1* |
| rs4536165 | 11 | 26018648 | 0.138 | 0.084 | 5.43E-04 | C | A | N | *TMEM16C* |
| rs11679161 | 2 | 239557946 | 0.242 | 0.070 | 5.45E-04 | T | C | N | *HDAC4* |
| rs2831897 | 21 | 28840934 | 0.125 | -0.089 | 5.45E-04 | T | C | Y | *N6AMT1* |
| rs17747187 | 16 | 20712750 | 0.103 | -0.098 | 5.46E-04 | T | C | N | *ACSM3* |
| rs1149996 | 10 | 9395673 | 0.420 | -0.059 | 5.46E-04 | G | A | Y | *GATA3* |
| rs11933929 | 4 | 14109624 | 0.554 | -0.059 | 5.46E-04 | C | T | N | *CPEB2* |
| rs13148890 | 4 | 14108251 | 0.446 | 0.059 | 5.48E-04 | C | T | N | *CPEB2* |
| rs9981994 | 21 | 28782701 | 0.123 | -0.091 | 5.48E-04 | T | C | N | *N6AMT1* |
| rs17158486 | 7 | 29929691 | 0.016 | -0.233 | 5.49E-04 | T | C | Y | *SCRN1* |
| rs17158506 | 7 | 29933095 | 0.016 | -0.233 | 5.49E-04 | T | C | Y | *SCRN1* |
| rs4420930 | 4 | 145540687 | 0.300 | -0.065 | 5.49E-04 | C | T | N | *HHIP* |
| rs11115693 | 12 | 82201056 | 0.122 | 0.093 | 5.51E-04 | T | C | N | *TMTC2* |
| rs4698258 | 4 | 14107907 | 0.446 | 0.059 | 5.52E-04 | T | C | N | *CPEB2* |
| rs7796692 | 7 | 32271390 | 0.282 | -0.065 | 5.53E-04 | G | A | N | *PDE1C* |
| rs2497772 | 10 | 18352792 | 0.525 | -0.058 | 5.53E-04 | T | C | Y | *SLC39A12* |
| rs12169625 | 22 | 24974715 | 0.181 | 0.076 | 5.53E-04 | A | G | Y | *SEZ6L* |
| rs7789284 | 7 | 69551698 | 0.052 | 0.133 | 5.53E-04 | G | T | N | *AUTS2* |
| rs10994614 | 10 | 62451097 | 0.037 | 0.158 | 5.53E-04 | C | G | N | *RHOBTB1* |
| rs1005424 | 1 | 166564089 | 0.315 | -0.065 | 5.54E-04 | T | C | N | *TBX19* |
| rs6460547 | 7 | 69551326 | 0.052 | 0.133 | 5.54E-04 | C | A | N | *AUTS2* |
| rs4698257 | 4 | 14107509 | 0.446 | 0.059 | 5.55E-04 | A | G | N | *CPEB2* |
| rs17708301 | 15 | 50680438 | 0.095 | 0.101 | 5.55E-04 | T | C | N | *KIAA1370* |
| rs10253217 | 7 | 69550375 | 0.052 | 0.133 | 5.55E-04 | G | A | N | *AUTS2* |
| rs12218739 | 10 | 62450584 | 0.037 | 0.158 | 5.55E-04 | A | G | N | *RHOBTB1* |
| rs10994613 | 10 | 62450133 | 0.037 | 0.158 | 5.55E-04 | A | G | N | *RHOBTB1* |
| rs4359162 | 11 | 26018784 | 0.138 | 0.084 | 5.56E-04 | G | T | N | *TMEM16C* |
| rs10230101 | 7 | 69564025 | 0.052 | 0.133 | 5.56E-04 | T | A | N | *AUTS2* |
| rs9851068 | 3 | 23814888 | 0.327 | -0.065 | 5.57E-04 | G | A | N | *UBE2E1* |
| rs10230510 | 7 | 69564352 | 0.052 | 0.133 | 5.57E-04 | T | A | Y | *AUTS2* |
| rs2295840 | 6 | 80780875 | 0.324 | -0.064 | 5.57E-04 | C | T | N | *TTK* |
| rs12113216 | 7 | 69543802 | 0.052 | 0.133 | 5.57E-04 | A | G | N | *AUTS2* |
| rs10175410 | 2 | 15804291 | 0.420 | 0.060 | 5.57E-04 | T | C | N | *DDX1* |
| rs934340 | 17 | 68304152 | 0.037 | -0.155 | 5.57E-04 | T | C | Y | *SLC39A11* |
| rs7563413 | 2 | 22915765 | 0.441 | 0.059 | 5.57E-04 | T | A | N | *ATAD2B* |
| rs10994612 | 10 | 62446948 | 0.037 | 0.158 | 5.58E-04 | G | A | N | *RHOBTB1* |
| rs10994611 | 10 | 62446794 | 0.037 | 0.158 | 5.58E-04 | A | T | N | *RHOBTB1* |
| rs414947 | 17 | 27915374 | 0.413 | 0.061 | 5.58E-04 | G | A | N | *MYO1D* |
| rs12111684 | 7 | 69541703 | 0.052 | 0.133 | 5.58E-04 | T | C | N | *AUTS2* |
| rs10746514 | 1 | 230312650 | 0.440 | 0.059 | 5.58E-04 | A | G | N | *DISC1* |
| rs1154122 | 14 | 36418566 | 0.411 | 0.060 | 5.59E-04 | A | G | N | *SLC25A21* |
| rs8041311 | 15 | 50674512 | 0.095 | 0.101 | 5.59E-04 | C | T | N | *KIAA1370* |
| rs2440375 | 15 | 50880552 | 0.118 | -0.093 | 5.60E-04 | A | C | N | *ONECUT1* |
| rs11606726 | 11 | 74182656 | 0.067 | 0.118 | 5.61E-04 | G | A | N | *RNF169* |
| rs881003 | 11 | 131110537 | 0.189 | 0.075 | 5.61E-04 | A | G | Y | *HNT* |
| rs10843649 | 12 | 30247503 | 0.172 | 0.078 | 5.61E-04 | A | G | Y | *TMTC1* |
| rs11728747 | 4 | 11239967 | 0.361 | 0.063 | 5.62E-04 | T | C | N | *HS3ST1* |
| rs1897870 | 6 | 80777005 | 0.324 | -0.064 | 5.62E-04 | T | C | N | *TTK* |
| rs4515923 | 11 | 26018841 | 0.138 | 0.084 | 5.63E-04 | C | G | N | *TMEM16C* |
| rs10894431 | 11 | 131112397 | 0.189 | 0.075 | 5.63E-04 | G | A | N | *HNT* |
| rs9983632 | 21 | 28846417 | 0.134 | -0.091 | 5.63E-04 | G | T | N | *N6AMT1* |
| rs1150000 | 10 | 9396301 | 0.420 | -0.059 | 5.64E-04 | C | T | N | *GATA3* |
| rs9958598 | 18 | 68431579 | 0.098 | -0.098 | 5.64E-04 | G | A | N | *CBLN2* |
| rs11505524 | 7 | 39247403 | 0.442 | 0.059 | 5.64E-04 | T | G | N | *POU6F2* |
| rs10009895 | 4 | 14105675 | 0.446 | 0.058 | 5.65E-04 | T | G | N | *CPEB2* |
| rs17036198 | 2 | 68969433 | 0.048 | -0.140 | 5.65E-04 | C | T | N | *BMP10* |
| rs7364225 | 22 | 16806080 | 0.025 | 0.189 | 5.65E-04 | T | C | N | *MICAL3* |
| rs1466749 | 6 | 170189052 | 0.372 | -0.061 | 5.65E-04 | G | A | N | *DLL1* |
| rs11819970 | 11 | 131112561 | 0.189 | 0.075 | 5.66E-04 | C | T | N | *HNT* |
| rs3799497 | 6 | 80776055 | 0.324 | -0.064 | 5.66E-04 | C | T | N | *TTK* |
| rs11222757 | 11 | 131113035 | 0.190 | 0.075 | 5.66E-04 | A | G | N | *HNT* |
| rs11764181 | 7 | 39242632 | 0.416 | -0.060 | 5.66E-04 | C | T | N | *POU6F2* |
| rs7071003 | 10 | 17080005 | 0.196 | -0.075 | 5.67E-04 | G | C | N | *CUBN* |
| rs11222758 | 11 | 131113178 | 0.190 | 0.075 | 5.67E-04 | C | T | N | *HNT* |
| rs379123 | 17 | 27915927 | 0.413 | 0.061 | 5.67E-04 | C | T | N | *MYO1D* |
| rs1949063 | 10 | 66579837 | 0.393 | 0.060 | 5.68E-04 | A | G | N | *CTNNA3* |
| rs13381576 | 18 | 68431972 | 0.098 | -0.098 | 5.68E-04 | T | C | Y | *CBLN2* |
| rs6836647 | 4 | 11255419 | 0.374 | 0.061 | 5.68E-04 | T | C | N | *HS3ST1* |
| rs9503976 | 6 | 4150396 | 0.133 | 0.087 | 5.69E-04 | G | C | Y | *PECI* |
| rs9986464 | 6 | 4150058 | 0.133 | 0.087 | 5.69E-04 | A | G | N | *PECI* |
| rs6934876 | 6 | 4142275 | 0.133 | 0.087 | 5.69E-04 | T | G | N | *PECI* |
| rs9503958 | 6 | 4141405 | 0.133 | 0.087 | 5.69E-04 | G | A | N | *PECI* |
| rs7749536 | 6 | 4138337 | 0.133 | 0.087 | 5.69E-04 | T | C | Y | *PECI* |
| rs2180311 | 1 | 1738594 | 0.501 | 0.058 | 5.69E-04 | T | C | N | *GNB1* |
| rs10012071 | 4 | 14103221 | 0.447 | 0.058 | 5.69E-04 | A | T | N | *CPEB2* |
| rs11612748 | 12 | 57293938 | 0.046 | -0.141 | 5.70E-04 | T | C | Y | *LRIG3* |
| rs372501 | 17 | 27916467 | 0.413 | 0.061 | 5.70E-04 | T | C | N | *MYO1D* |
| rs17502484 | 1 | 166547227 | 0.330 | -0.063 | 5.70E-04 | T | C | N | *TBX19* |
| rs1150001 | 10 | 9396637 | 0.420 | -0.059 | 5.71E-04 | G | A | N | *GATA3* |
| rs4146573 | 4 | 14103079 | 0.447 | 0.058 | 5.71E-04 | T | C | N | *CPEB2* |
| rs13107272 | 4 | 11244386 | 0.399 | 0.059 | 5.75E-04 | C | A | Y | *HS3ST1* |
| rs2242801 | 21 | 36424820 | 0.478 | -0.058 | 5.75E-04 | G | T | N | *CBR3* |
| rs4329014 | 5 | 113057813 | 0.138 | -0.084 | 5.75E-04 | C | T | Y | *YTHDC2* |
| rs796676 | 3 | 193752340 | 0.412 | 0.059 | 5.75E-04 | A | C | Y | *FGF12* |
| rs4376087 | 4 | 145540805 | 0.292 | -0.064 | 5.76E-04 | C | T | N | *HHIP* |
| rs6664664 | 1 | 1741124 | 0.501 | 0.058 | 5.76E-04 | G | A | N | *GNB1* |
| rs1150002 | 10 | 9396735 | 0.420 | -0.059 | 5.76E-04 | G | A | Y | *GATA3* |
| rs2120440 | 15 | 50913076 | 0.040 | -0.152 | 5.76E-04 | G | A | N | *ONECUT1* |
| rs1111963 | 4 | 14100544 | 0.447 | 0.058 | 5.77E-04 | T | C | N | *CPEB2* |
| rs725854 | 11 | 26013376 | 0.139 | 0.084 | 5.77E-04 | A | G | N | *TMEM16C* |
| rs13137646 | 4 | 180242434 | 0.272 | -0.066 | 5.79E-04 | A | G | N | *LOC285501* |
| rs13137373 | 4 | 180242278 | 0.272 | -0.066 | 5.79E-04 | A | G | N | *LOC285501* |
| rs10283892 | 9 | 72357417 | 0.549 | 0.059 | 5.79E-04 | A | C | N | *TRPM3* |
| rs2784956 | 6 | 924803 | 0.390 | -0.063 | 5.80E-04 | T | C | N | *EXOC2* |
| rs2867716 | 4 | 14089390 | 0.447 | 0.058 | 5.80E-04 | G | A | N | *CPEB2* |
| rs2357782 | 10 | 18354073 | 0.271 | 0.065 | 5.80E-04 | T | C | Y | *SLC39A12* |
| rs7563283 | 2 | 22915635 | 0.440 | 0.059 | 5.83E-04 | T | C | Y | *ATAD2B* |
| rs10501420 | 11 | 74173685 | 0.066 | 0.118 | 5.83E-04 | T | C | N | *RNF169* |
| rs6062199 | 20 | 60305675 | 0.043 | -0.144 | 5.83E-04 | A | G | N | *OSBPL2* |
| rs10846880 | 12 | 124412668 | 0.413 | -0.060 | 5.85E-04 | G | C | N | *TMEM132B* |
| rs10014650 | 4 | 180241004 | 0.272 | -0.066 | 5.85E-04 | C | T | N | *LOC285501* |
| rs11792922 | 9 | 80364394 | 0.359 | 0.061 | 5.85E-04 | G | A | N | *PSAT1* |
| rs7676032 | 4 | 145540518 | 0.292 | -0.064 | 5.86E-04 | G | C | N | *HHIP* |
| rs4652301 | 1 | 176766422 | 0.376 | 0.061 | 5.86E-04 | C | T | N | *C1orf49* |
| rs1150008 | 10 | 9397614 | 0.420 | -0.059 | 5.86E-04 | A | T | N | *GATA3* |
| rs9552821 | 13 | 22566828 | 0.372 | 0.060 | 5.87E-04 | C | T | N | *SGCG* |
| rs4536166 | 11 | 26019050 | 0.138 | 0.084 | 5.87E-04 | C | T | N | *TMEM16C* |
| rs2158521 | 17 | 61025204 | 0.159 | -0.080 | 5.87E-04 | A | C | Y | *CCDC46* |
| rs4143149 | 4 | 180241683 | 0.272 | -0.066 | 5.88E-04 | A | G | N | *LOC285501* |
| rs965627 | 20 | 16061273 | 0.014 | 0.245 | 5.89E-04 | G | A | Y | *MACROD2* |
| rs6841368 | 4 | 180240619 | 0.272 | -0.066 | 5.89E-04 | T | C | N | *LOC285501* |
| rs1542726 | 4 | 145735219 | 0.436 | -0.060 | 5.90E-04 | A | C | N | *HHIP* |
| rs7861457 | 9 | 80367022 | 0.359 | 0.061 | 5.90E-04 | C | G | N | *PSAT1* |
| rs17666267 | 18 | 58234198 | 0.231 | -0.070 | 5.90E-04 | A | C | Y | *TNFRSF11A* |
| rs10488988 | 4 | 11245261 | 0.356 | 0.061 | 5.91E-04 | T | C | Y | *HS3ST1* |
| rs17794470 | 15 | 69506060 | 0.058 | -0.123 | 5.91E-04 | T | G | Y | *LOC645296* |
| rs9981858 | 21 | 28789819 | 0.123 | -0.090 | 5.92E-04 | A | C | N | *N6AMT1* |
| rs10020675 | 4 | 14087374 | 0.447 | 0.058 | 5.92E-04 | T | A | N | *CPEB2* |
| rs10488083 | 7 | 29959939 | 0.016 | -0.234 | 5.92E-04 | C | T | N | *SCRN1* |
| rs8134362 | 21 | 28793272 | 0.123 | -0.090 | 5.92E-04 | A | G | N | *N6AMT1* |
| rs10488987 | 4 | 11245311 | 0.356 | 0.061 | 5.92E-04 | A | G | N | *HS3ST1* |
| rs1003749 | 7 | 29965249 | 0.015 | -0.234 | 5.93E-04 | G | A | Y | *SCRN1* |
| rs8098721 | 18 | 68446160 | 0.098 | -0.099 | 5.94E-04 | T | C | N | *CBLN2* |
| rs3975414 | 4 | 11245698 | 0.357 | 0.061 | 5.94E-04 | G | C | N | *HS3ST1* |
| rs4677337 | 3 | 73851210 | 0.357 | -0.061 | 5.94E-04 | A | G | N | *PDZRN3* |
| rs1501164 | 4 | 14086302 | 0.447 | 0.058 | 5.95E-04 | A | G | N | *CPEB2* |
| rs12467854 | 2 | 239553695 | 0.243 | 0.069 | 5.95E-04 | G | C | N | *HDAC4* |
| rs1155947 | 3 | 80460159 | 0.258 | -0.067 | 5.96E-04 | T | C | N | *ROBO1* |
| rs3960974 | 4 | 11245884 | 0.357 | 0.061 | 5.96E-04 | T | C | N | *HS3ST1* |
| rs10265739 | 7 | 39241463 | 0.555 | -0.059 | 5.96E-04 | A | G | Y | *POU6F2* |
| rs7592121 | 2 | 154663133 | 0.071 | -0.116 | 5.96E-04 | G | T | N | *GALNT13* |
| rs4814433 | 20 | 16075261 | 0.014 | 0.245 | 5.96E-04 | C | T | N | *MACROD2* |
| rs12817332 | 12 | 18898133 | 0.115 | 0.092 | 5.97E-04 | A | C | N | *CAPZA3* |
| rs9552818 | 13 | 22566142 | 0.371 | 0.060 | 5.97E-04 | G | C | N | *SGCG* |
| rs1360811 | 6 | 4153071 | 0.133 | 0.086 | 5.97E-04 | T | C | N | *PECI* |
| rs3960973 | 4 | 11245895 | 0.357 | 0.061 | 5.98E-04 | A | G | N | *HS3ST1* |
| rs3737628 | 1 | 1712792 | 0.504 | 0.058 | 5.98E-04 | C | T | Y | *GNB1* |
| rs2089271 | 5 | 90985090 | 0.526 | 0.059 | 5.98E-04 | C | T | N | *ARRDC3* |
| rs9979334 | 21 | 28797843 | 0.123 | -0.089 | 5.98E-04 | C | G | N | *N6AMT1* |
| rs1323303 | 20 | 16076300 | 0.014 | 0.245 | 5.98E-04 | A | C | N | *MACROD2* |
| rs4814434 | 20 | 16075428 | 0.014 | 0.245 | 5.99E-04 | C | A | N | *MACROD2* |
| rs17805818 | 4 | 146800819 | 0.069 | -0.115 | 5.99E-04 | G | A | Y | *MMAA* |
| rs9378848 | 6 | 4122526 | 0.270 | 0.066 | 5.99E-04 | C | T | N | *PECI* |
| rs9959310 | 18 | 58232847 | 0.231 | -0.070 | 5.99E-04 | C | T | N | *TNFRSF11A* |
| rs1990426 | 7 | 68960042 | 0.050 | 0.135 | 5.99E-04 | G | A | N | *AUTS2* |
| rs7749039 | 6 | 4138002 | 0.133 | 0.086 | 6.00E-04 | T | C | N | *PECI* |
| rs712382 | 14 | 36413502 | 0.403 | 0.059 | 6.00E-04 | A | G | N | *SLC25A21* |
| rs10929383 | 2 | 15805177 | 0.427 | 0.059 | 6.00E-04 | T | C | Y | *DDX1* |
| rs848038 | 14 | 36410203 | 0.403 | 0.059 | 6.00E-04 | G | C | N | *SLC25A21* |
| rs391062 | 21 | 39470137 | 0.065 | -0.119 | 6.00E-04 | C | T | Y | *DSCR2* |
| rs6517520 | 21 | 39470768 | 0.065 | -0.119 | 6.00E-04 | A | C | Y | *DSCR2* |
| rs848037 | 14 | 36410226 | 0.403 | 0.059 | 6.01E-04 | T | A | N | *SLC25A21* |
| rs6927465 | 6 | 170192495 | 0.370 | -0.060 | 6.01E-04 | A | C | N | *DLL1* |
| rs2662796 | 12 | 126187285 | 0.104 | -0.095 | 6.02E-04 | A | G | Y | *TMEM132B* |
| rs9978208 | 21 | 28802839 | 0.123 | -0.089 | 6.02E-04 | G | C | N | *N6AMT1* |
| rs6569798 | 6 | 132820231 | 0.441 | -0.058 | 6.02E-04 | T | C | N | *STX7* |
| rs9660180 | 1 | 1712891 | 0.496 | -0.058 | 6.03E-04 | A | G | N | *GNB1* |
| rs11164844 | 1 | 93189238 | 0.375 | 0.059 | 6.03E-04 | A | C | N | *FAM69A* |
| rs7963810 | 12 | 12200363 | 0.177 | -0.077 | 6.03E-04 | C | T | N | *LRP6* |
| rs9604809 | 22 | 16800096 | 0.025 | 0.189 | 6.04E-04 | C | T | N | *MICAL3* |
| rs4735558 | 8 | 99483750 | 0.146 | -0.083 | 6.04E-04 | G | A | N | *KCNS2* |
| rs1477033 | 7 | 77934415 | 0.454 | 0.060 | 6.04E-04 | C | G | N | *MAGI2* |
| rs218824 | 4 | 14081020 | 0.447 | 0.058 | 6.05E-04 | C | G | N | *CPEB2* |
| rs6560143 | 9 | 72358500 | 0.549 | 0.059 | 6.05E-04 | T | C | N | *TRPM3* |
| rs9367279 | 6 | 47556295 | 0.266 | -0.066 | 6.06E-04 | G | A | N | *CD2AP* |
| rs4734404 | 8 | 99485656 | 0.146 | -0.083 | 6.06E-04 | A | G | N | *KCNS2* |
| rs280659 | 9 | 98032498 | 0.268 | -0.065 | 6.07E-04 | G | C | N | *HSD17B3* |
| rs712377 | 14 | 36406723 | 0.402 | 0.059 | 6.07E-04 | C | A | Y | *SLC25A21* |
| rs9502132 | 6 | 4154131 | 0.133 | 0.086 | 6.07E-04 | T | G | N | *PECI* |
| rs12216886 | 9 | 2483751 | 0.199 | 0.075 | 6.07E-04 | G | T | N | *VLDLR* |
| rs6062140 | 20 | 60206436 | 0.356 | 0.061 | 6.08E-04 | C | G | N | *GTPBP5* |
| rs3739776 | 9 | 72341790 | 0.444 | -0.059 | 6.09E-04 | C | T | N | *TRPM3* |
| rs9982122 | 21 | 39469180 | 0.065 | -0.118 | 6.10E-04 | A | G | N | *DSCR2* |
| rs6560142 | 9 | 72340804 | 0.444 | -0.059 | 6.10E-04 | C | T | Y | *TRPM3* |
| rs712376 | 14 | 36406567 | 0.402 | 0.059 | 6.10E-04 | C | T | N | *SLC25A21* |
| rs13106971 | 4 | 11238980 | 0.368 | 0.063 | 6.11E-04 | G | A | N | *HS3ST1* |
| rs13433486 | 21 | 28807010 | 0.123 | -0.089 | 6.11E-04 | T | C | N | *N6AMT1* |
| rs8019150 | 14 | 51715553 | 0.024 | 0.191 | 6.11E-04 | T | C | N | *C14orf166* |
| rs986633 | 8 | 87704567 | 0.084 | -0.109 | 6.11E-04 | A | G | N | *CNGB3* |
| rs11728092 | 4 | 11240544 | 0.368 | 0.062 | 6.11E-04 | A | G | N | *HS3ST1* |
| rs712375 | 14 | 36406412 | 0.402 | 0.059 | 6.12E-04 | C | T | N | *SLC25A21* |
| rs2416325 | 5 | 113046807 | 0.139 | -0.083 | 6.12E-04 | C | A | N | *YTHDC2* |
| rs12320259 | 12 | 12191436 | 0.176 | -0.079 | 6.12E-04 | T | C | N | *LRP6* |
| rs6933147 | 6 | 4154534 | 0.133 | 0.086 | 6.12E-04 | T | G | N | *PECI* |
| rs4284942 | 3 | 80459834 | 0.258 | -0.067 | 6.13E-04 | C | G | N | *ROBO1* |
| rs765117 | 12 | 30300102 | 0.421 | 0.059 | 6.13E-04 | A | G | N | *IPO8* |
| rs848040 | 14 | 36406273 | 0.402 | 0.059 | 6.13E-04 | T | C | N | *SLC25A21* |
| rs1426387 | 11 | 115479382 | 0.218 | 0.070 | 6.14E-04 | C | G | Y | *CADM1* |
| rs1866907 | 8 | 87710519 | 0.084 | -0.108 | 6.14E-04 | T | G | N | *CNGB3* |
| rs10746848 | 9 | 72361833 | 0.548 | 0.058 | 6.16E-04 | T | G | Y | *TRPM3* |
| rs2134635 | 3 | 193758289 | 0.411 | 0.059 | 6.16E-04 | A | G | N | *FGF12* |
| rs218822 | 4 | 14080621 | 0.447 | 0.058 | 6.17E-04 | A | C | N | *CPEB2* |
| rs12233607 | 3 | 73835369 | 0.116 | -0.092 | 6.17E-04 | C | T | N | *PDZRN3* |
| rs4744603 | 9 | 72359556 | 0.548 | 0.058 | 6.17E-04 | G | A | N | *TRPM3* |
| rs17380908 | 1 | 93175139 | 0.374 | 0.059 | 6.18E-04 | T | C | Y | *FAM69A* |
| rs2171086 | 6 | 47698063 | 0.270 | -0.065 | 6.18E-04 | T | C | N | *CD2AP* |
| rs10948367 | 6 | 47693574 | 0.270 | -0.065 | 6.18E-04 | G | A | Y | *CD2AP* |
| rs13135711 | 4 | 11247955 | 0.399 | 0.059 | 6.19E-04 | C | G | N | *HS3ST1* |
| rs6604028 | 1 | 93150621 | 0.374 | 0.059 | 6.19E-04 | T | C | N | *FAM69A* |
| rs9395286 | 6 | 47683291 | 0.270 | -0.065 | 6.19E-04 | C | T | N | *CD2AP* |
| rs2396825 | 6 | 47676655 | 0.270 | -0.065 | 6.20E-04 | G | A | N | *CD2AP* |
| rs12074608 | 1 | 93184369 | 0.374 | 0.059 | 6.20E-04 | C | T | N | *FAM69A* |
| rs9381578 | 6 | 47664593 | 0.270 | -0.065 | 6.21E-04 | T | C | N | *CD2AP* |
| rs9369717 | 6 | 47662427 | 0.270 | -0.065 | 6.21E-04 | G | T | N | *CD2AP* |
| rs10994608 | 10 | 62432576 | 0.037 | 0.157 | 6.21E-04 | A | C | N | *RHOBTB1* |
| rs723162 | 4 | 14079691 | 0.447 | 0.058 | 6.21E-04 | A | G | N | *CPEB2* |
| rs9395285 | 6 | 47662136 | 0.270 | -0.065 | 6.21E-04 | A | G | N | *CD2AP* |
| rs9369716 | 6 | 47660139 | 0.270 | -0.065 | 6.21E-04 | T | A | N | *CD2AP* |
| rs9349416 | 6 | 47659897 | 0.270 | -0.065 | 6.22E-04 | A | G | N | *CD2AP* |
| rs11164843 | 1 | 93188730 | 0.374 | 0.059 | 6.22E-04 | C | A | N | *FAM69A* |
| rs9395283 | 6 | 47659403 | 0.270 | -0.065 | 6.22E-04 | C | T | N | *CD2AP* |
| rs9341801 | 6 | 80771882 | 0.323 | -0.064 | 6.22E-04 | A | G | N | *TTK* |
| rs1262398 | 10 | 76791088 | 0.346 | 0.063 | 6.22E-04 | C | T | N | *ZNF503* |
| rs359063 | 10 | 132140580 | 0.237 | 0.069 | 6.22E-04 | T | C | N | *GLRX3* |
| rs2309743 | 9 | 72363029 | 0.549 | 0.058 | 6.22E-04 | T | C | N | *TRPM3* |
| rs1991719 | 18 | 68437374 | 0.098 | -0.098 | 6.22E-04 | G | A | N | *CBLN2* |
| rs9974272 | 21 | 41443581 | 0.217 | -0.071 | 6.23E-04 | G | A | Y | *BACE2* |
| rs870118 | 1 | 212572312 | 0.114 | 0.091 | 6.23E-04 | C | G | N | *SMYD2* |
| rs10742128 | 11 | 26021844 | 0.139 | 0.083 | 6.24E-04 | G | A | Y | *TMEM16C* |
| rs6507051 | 18 | 29462451 | 0.499 | 0.058 | 6.25E-04 | A | G | N | *ASXL3* |
| rs1173215 | 5 | 4878798 | 0.085 | 0.104 | 6.25E-04 | C | T | N | *ADAMTS16* |
| rs2488696 | 10 | 76751854 | 0.448 | -0.060 | 6.25E-04 | T | C | N | *ZNF503* |
| rs7077421 | 10 | 76793163 | 0.346 | 0.063 | 6.25E-04 | T | C | N | *ZNF503* |
| rs8088700 | 18 | 68435477 | 0.098 | -0.097 | 6.26E-04 | A | C | N | *CBLN2* |
| rs16962432 | 16 | 82584682 | 0.029 | -0.181 | 6.26E-04 | T | C | N | *EFCBP2* |
| rs9975371 | 21 | 28817851 | 0.123 | -0.089 | 6.26E-04 | T | C | N | *N6AMT1* |
| rs17137823 | 6 | 4155169 | 0.133 | 0.086 | 6.26E-04 | C | G | N | *PECI* |
| rs525502 | 3 | 174692688 | 0.115 | -0.090 | 6.27E-04 | C | T | Y | *NLGN1* |
| rs11726412 | 4 | 145573473 | 0.298 | -0.064 | 6.27E-04 | A | C | N | *HHIP* |
| rs6484193 | 11 | 26020111 | 0.138 | 0.083 | 6.27E-04 | A | G | N | *TMEM16C* |
| rs988513 | 11 | 26021935 | 0.139 | 0.083 | 6.27E-04 | T | C | N | *TMEM16C* |
| rs11164845 | 1 | 93189273 | 0.374 | 0.059 | 6.28E-04 | G | A | N | *FAM69A* |
| rs6142990 | 20 | 60207430 | 0.356 | 0.061 | 6.28E-04 | G | A | Y | *GTPBP5* |
| rs9239 | 6 | 132822461 | 0.438 | -0.058 | 6.28E-04 | G | A | N | *STX7* |
| rs9319846 | 18 | 68434761 | 0.098 | -0.097 | 6.28E-04 | C | T | N | *CBLN2* |
| rs10213287 | 4 | 114139586 | 0.157 | 0.080 | 6.28E-04 | T | A | Y | *ANK2* |
| rs17086171 | 18 | 68375431 | 0.111 | -0.093 | 6.28E-04 | G | A | Y | *CBLN2* |
| rs9349413 | 6 | 47619450 | 0.270 | -0.065 | 6.29E-04 | G | A | N | *CD2AP* |
| rs9319845 | 18 | 68434345 | 0.098 | -0.097 | 6.29E-04 | C | T | Y | *CBLN2* |
| rs7872938 | 9 | 72367323 | 0.549 | 0.058 | 6.30E-04 | A | G | N | *TRPM3* |
| rs765315 | 3 | 179856570 | 0.171 | -0.078 | 6.30E-04 | C | G | N | *KCNMB2* |
| rs988514 | 11 | 26022038 | 0.139 | 0.083 | 6.31E-04 | A | G | N | *TMEM16C* |
| rs7754282 | 6 | 47609983 | 0.270 | -0.065 | 6.31E-04 | C | G | N | *CD2AP* |
| rs1934471 | 9 | 72369031 | 0.548 | 0.058 | 6.31E-04 | A | G | N | *TRPM3* |
| rs7996937 | 13 | 22566775 | 0.363 | 0.060 | 6.32E-04 | G | T | Y | *SGCG* |
| rs2871324 | 9 | 72369797 | 0.548 | 0.058 | 6.32E-04 | T | C | N | *TRPM3* |
| rs9552823 | 13 | 22567666 | 0.363 | 0.060 | 6.33E-04 | A | T | N | *SGCG* |
| rs6602171 | 10 | 17080243 | 0.204 | -0.073 | 6.33E-04 | A | G | N | *CUBN* |
| rs9827383 | 3 | 41363412 | 0.223 | 0.072 | 6.34E-04 | C | G | N | *ULK4* |
| rs988515 | 11 | 26022059 | 0.139 | 0.083 | 6.35E-04 | A | G | N | *TMEM16C* |
| rs7749167 | 6 | 47601899 | 0.269 | -0.065 | 6.35E-04 | A | G | N | *CD2AP* |
| rs12060952 | 1 | 93153861 | 0.374 | 0.059 | 6.36E-04 | C | T | N | *FAM69A* |
| rs12059883 | 1 | 93153696 | 0.374 | 0.059 | 6.36E-04 | C | T | N | *FAM69A* |
| rs9651257 | 1 | 93157724 | 0.374 | 0.059 | 6.36E-04 | C | T | Y | *FAM69A* |
| rs11164835 | 1 | 93151681 | 0.374 | 0.059 | 6.36E-04 | A | G | Y | *FAM69A* |
| rs10874751 | 1 | 93157128 | 0.374 | 0.059 | 6.36E-04 | A | T | N | *FAM69A* |
| rs17380789 | 1 | 93160107 | 0.374 | 0.059 | 6.36E-04 | T | C | N | *FAM69A* |
| rs3760849 | 19 | 61645397 | 0.502 | 0.058 | 6.36E-04 | T | C | Y | *ZNF667* |
| rs12745968 | 1 | 93174425 | 0.374 | 0.059 | 6.36E-04 | G | A | Y | *FAM69A* |
| rs7417198 | 1 | 93164033 | 0.374 | 0.059 | 6.36E-04 | C | T | N | *FAM69A* |
| rs6698233 | 1 | 93169888 | 0.374 | 0.059 | 6.36E-04 | T | C | N | *FAM69A* |
| rs7542608 | 1 | 93173782 | 0.374 | 0.059 | 6.36E-04 | G | A | N | *FAM69A* |
| rs7515257 | 1 | 93166306 | 0.374 | 0.059 | 6.36E-04 | A | G | N | *FAM69A* |
| rs6604030 | 1 | 93170924 | 0.374 | 0.059 | 6.36E-04 | G | A | N | *FAM69A* |
| rs7550347 | 1 | 93173745 | 0.374 | 0.059 | 6.36E-04 | C | G | N | *FAM69A* |
| rs6604029 | 1 | 93166713 | 0.374 | 0.059 | 6.36E-04 | G | C | N | *FAM69A* |
| rs10734366 | 11 | 26020535 | 0.138 | 0.083 | 6.36E-04 | C | T | N | *TMEM16C* |
| rs11164836 | 1 | 93154003 | 0.374 | 0.059 | 6.36E-04 | T | C | N | *FAM69A* |
| rs9267444 | 6 | 31591437 | 0.326 | -0.065 | 6.37E-04 | A | G | N | *MICB* |
| rs17152432 | 5 | 124575681 | 0.390 | -0.059 | 6.37E-04 | C | A | N | *ZNF608* |
| rs7133142 | 12 | 115835358 | 0.012 | -0.265 | 6.37E-04 | C | T | N | *FBXW8* |
| rs17068473 | 8 | 3842395 | 0.249 | -0.067 | 6.37E-04 | T | G | Y | *CSMD1* |
| rs12633552 | 3 | 73845979 | 0.116 | -0.092 | 6.38E-04 | A | G | N | *PDZRN3* |
| rs11701356 | 21 | 41444833 | 0.217 | -0.071 | 6.38E-04 | C | T | N | *BACE2* |
| rs988516 | 11 | 26022176 | 0.139 | 0.083 | 6.39E-04 | G | A | N | *TMEM16C* |
| rs9804898 | 12 | 27820624 | 0.431 | 0.061 | 6.39E-04 | G | A | N | *KLHDC5* |
| rs9503947 | 6 | 4134875 | 0.133 | 0.086 | 6.39E-04 | G | A | Y | *PECI* |
| rs718403 | 12 | 12188223 | 0.175 | -0.077 | 6.39E-04 | A | G | N | *LRP6* |
| rs9296561 | 6 | 47596897 | 0.269 | -0.065 | 6.39E-04 | A | G | N | *CD2AP* |
| rs10948363 | 6 | 47595721 | 0.269 | -0.065 | 6.41E-04 | G | A | N | *CD2AP* |
| rs13169195 | 5 | 43268299 | 0.060 | 0.122 | 6.41E-04 | G | C | Y | *MGC42105* |
| rs7761775 | 6 | 4158143 | 0.133 | 0.086 | 6.42E-04 | G | C | Y | *PECI* |
| rs413078 | 21 | 39464565 | 0.065 | -0.118 | 6.42E-04 | C | T | N | *DSCR2* |
| rs4715025 | 6 | 47591612 | 0.269 | -0.065 | 6.42E-04 | G | C | N | *CD2AP* |
| rs3096715 | 11 | 86133524 | 0.406 | -0.062 | 6.43E-04 | T | C | N | *PRSS23* |
| rs11701851 | 21 | 41445722 | 0.217 | -0.071 | 6.43E-04 | T | C | N | *BACE2* |
| rs789538 | 11 | 131049823 | 0.247 | 0.068 | 6.43E-04 | C | T | N | *HNT* |
| rs1336845 | 13 | 22569613 | 0.363 | 0.060 | 6.44E-04 | G | A | N | *SGCG* |
| rs7040145 | 9 | 72348383 | 0.450 | -0.058 | 6.44E-04 | G | A | N | *TRPM3* |
| rs10519987 | 4 | 149916855 | 0.322 | -0.062 | 6.44E-04 | T | A | N | *NR3C2* |
| rs1872505 | 6 | 47588934 | 0.269 | -0.065 | 6.45E-04 | G | C | N | *CD2AP* |
| rs12981146 | 19 | 61654276 | 0.498 | 0.058 | 6.45E-04 | C | G | N | *ZNF667* |
| rs1652148 | 12 | 30300742 | 0.422 | 0.059 | 6.46E-04 | A | G | Y | *IPO8* |
| rs17303482 | 5 | 43272465 | 0.060 | 0.122 | 6.46E-04 | A | G | N | *MGC42105* |
| rs11771925 | 7 | 39243478 | 0.443 | 0.059 | 6.46E-04 | G | A | Y | *POU6F2* |
| rs7738044 | 6 | 47577232 | 0.269 | -0.065 | 6.48E-04 | G | A | N | *CD2AP* |
| rs7328594 | 13 | 40151764 | 0.295 | 0.066 | 6.48E-04 | C | T | N | *FOXO1* |
| rs12667891 | 7 | 39241995 | 0.442 | 0.059 | 6.49E-04 | A | G | Y | *POU6F2* |
| rs16944974 | 18 | 24418623 | 0.260 | 0.066 | 6.50E-04 | A | G | N | *CDH2* |
| rs1548994 | 16 | 49416312 | 0.042 | -0.149 | 6.50E-04 | A | C | N | *CYLD* |
| rs12701158 | 7 | 31917684 | 0.287 | -0.064 | 6.50E-04 | A | T | N | *PDE1C* |
| rs1387503 | 11 | 26022994 | 0.139 | 0.083 | 6.50E-04 | A | G | N | *TMEM16C* |
| rs6812106 | 4 | 114140554 | 0.154 | 0.081 | 6.50E-04 | T | C | N | *ANK2* |
| rs2295838 | 6 | 80770680 | 0.323 | -0.064 | 6.50E-04 | T | C | N | *TTK* |
| rs307385 | 10 | 132140656 | 0.237 | 0.069 | 6.51E-04 | T | G | Y | *GLRX3* |
| rs10270969 | 7 | 96885565 | 0.045 | -0.147 | 6.51E-04 | A | T | N | *ACN9* |
| rs12667875 | 7 | 39241900 | 0.442 | 0.059 | 6.51E-04 | C | G | N | *POU6F2* |
| rs1806788 | 9 | 72365196 | 0.547 | 0.058 | 6.51E-04 | A | G | N | *TRPM3* |
| rs2658873 | 11 | 131106413 | 0.185 | 0.075 | 6.52E-04 | A | G | N | *HNT* |
| rs1589881 | 7 | 45455831 | 0.210 | -0.073 | 6.53E-04 | A | G | N | *ADCY1* |
| rs2358649 | 4 | 149861274 | 0.251 | -0.067 | 6.53E-04 | A | C | Y | *NR3C2* |
| rs12672115 | 7 | 39241775 | 0.558 | -0.059 | 6.54E-04 | A | G | N | *POU6F2* |
| rs9415797 | 10 | 66652220 | 0.282 | 0.065 | 6.54E-04 | T | A | N | *CTNNA3* |
| rs8033889 | 15 | 69467134 | 0.199 | -0.072 | 6.54E-04 | T | G | Y | *LOC645296* |
| rs789545 | 11 | 131101784 | 0.185 | 0.075 | 6.55E-04 | T | C | Y | *HNT* |
| rs1506666 | 11 | 131103370 | 0.185 | 0.075 | 6.55E-04 | T | C | Y | *HNT* |
| rs10834897 | 11 | 26023381 | 0.139 | 0.083 | 6.55E-04 | T | C | N | *TMEM16C* |
| rs4745016 | 9 | 72368482 | 0.547 | 0.058 | 6.56E-04 | A | G | N | *TRPM3* |
| rs10976619 | 9 | 7834368 | 0.489 | -0.057 | 6.56E-04 | A | T | Y | *C9orf123* |
| rs17121329 | 10 | 91178363 | 0.028 | 0.183 | 6.56E-04 | T | G | N | *SLC16A12* |
| rs13079502 | 3 | 66855115 | 0.100 | 0.098 | 6.57E-04 | T | C | N | *LRIG1* |
| rs9879990 | 3 | 66856285 | 0.100 | 0.098 | 6.59E-04 | T | C | N | *LRIG1* |
| rs9296558 | 6 | 47559842 | 0.268 | -0.065 | 6.59E-04 | T | C | Y | *CD2AP* |
| rs6604026 | 1 | 93076191 | 0.287 | 0.063 | 6.59E-04 | C | T | Y | *RPL5* |
| rs10943681 | 6 | 80769963 | 0.323 | -0.064 | 6.60E-04 | C | A | N | *TTK* |
| rs7105339 | 11 | 26021655 | 0.138 | 0.083 | 6.60E-04 | T | C | N | *TMEM16C* |
| rs10446793 | 4 | 61807126 | 0.240 | -0.068 | 6.60E-04 | G | A | Y | *LPHN3* |
| rs11133883 | 5 | 1776309 | 0.308 | 0.064 | 6.61E-04 | C | T | N | *MRPL36* |
| rs11949447 | 5 | 1776445 | 0.309 | 0.064 | 6.61E-04 | T | C | N | *MRPL36* |
| rs583697 | 11 | 125685230 | 0.302 | 0.065 | 6.62E-04 | C | T | N | *DCPS* |
| rs1731513 | 12 | 30299364 | 0.422 | 0.059 | 6.62E-04 | C | G | N | *IPO8* |
| rs7860549 | 9 | 79370561 | 0.088 | 0.106 | 6.63E-04 | G | A | N | *GNA14* |
| rs9502136 | 6 | 4165043 | 0.133 | 0.086 | 6.63E-04 | A | G | N | *PECI* |
| rs10742127 | 11 | 26020915 | 0.138 | 0.083 | 6.63E-04 | G | T | N | *TMEM16C* |
| rs4677315 | 3 | 73743431 | 0.518 | -0.057 | 6.63E-04 | T | G | N | *PDZRN3* |
| rs1895135 | 2 | 15798223 | 0.410 | 0.059 | 6.63E-04 | A | G | N | *DDX1* |
| rs9320787 | 6 | 121362384 | 0.208 | 0.071 | 6.64E-04 | A | G | N | *C6orf170* |
| rs165226 | 2 | 164545327 | 0.262 | 0.066 | 6.64E-04 | T | C | N | *FIGN* |
| rs1652153 | 12 | 30299250 | 0.422 | 0.059 | 6.65E-04 | T | G | N | *IPO8* |
| rs595760 | 11 | 125684872 | 0.302 | 0.065 | 6.65E-04 | T | C | N | *DCPS* |
| rs165224 | 2 | 164545879 | 0.262 | 0.066 | 6.65E-04 | T | G | N | *FIGN* |
| rs4479449 | 2 | 75934419 | 0.120 | 0.089 | 6.65E-04 | G | A | N | *C2orf3* |
| rs4715019 | 6 | 47555000 | 0.268 | -0.065 | 6.66E-04 | A | T | N | *CD2AP* |
| rs10201377 | 2 | 68116661 | 0.166 | 0.081 | 6.66E-04 | G | C | N | *C1D* |
| rs2904669 | 8 | 17097693 | 0.555 | -0.060 | 6.66E-04 | A | G | N | *ZDHHC2* |
| rs7733169 | 5 | 63394265 | 0.225 | -0.069 | 6.66E-04 | G | A | Y | *HTR1A* |
| rs1548506 | 19 | 41278649 | 0.441 | 0.058 | 6.66E-04 | C | G | N | *WDR62* |
| rs17837302 | 13 | 71544717 | 0.179 | 0.077 | 6.67E-04 | C | T | N | *DACH1* |
| rs9503980 | 6 | 4165282 | 0.133 | 0.086 | 6.67E-04 | G | A | N | *PECI* |
| rs7115939 | 11 | 26021025 | 0.138 | 0.083 | 6.67E-04 | C | A | N | *TMEM16C* |
| rs9503981 | 6 | 4165299 | 0.133 | 0.086 | 6.68E-04 | T | G | N | *PECI* |
| rs4787394 | 16 | 26874852 | 0.334 | -0.061 | 6.68E-04 | A | G | N | *C16orf82* |
| rs736780 | 15 | 50891469 | 0.172 | -0.079 | 6.68E-04 | G | A | N | *ONECUT1* |
| rs17583864 | 4 | 149925302 | 0.322 | -0.062 | 6.69E-04 | C | A | Y | *NR3C2* |
| rs732648 | 6 | 53230100 | 0.396 | -0.060 | 6.69E-04 | T | C | N | *ELOVL5* |
| rs17034864 | 2 | 68116207 | 0.166 | 0.081 | 6.69E-04 | T | C | N | *C1D* |
| rs7116083 | 11 | 26021208 | 0.138 | 0.083 | 6.70E-04 | A | G | N | *TMEM16C* |
| rs1582815 | 13 | 87597244 | 0.387 | 0.059 | 6.71E-04 | A | G | Y | *SLITRK5* |
| rs17087341 | 6 | 156732172 | 0.177 | -0.076 | 6.71E-04 | C | T | Y | *ARID1B* |
| rs12859192 | 13 | 87597825 | 0.387 | 0.059 | 6.71E-04 | A | G | N | *SLITRK5* |
| rs10976618 | 9 | 7833141 | 0.487 | -0.057 | 6.72E-04 | C | T | Y | *C9orf123* |
| rs1387507 | 11 | 26023848 | 0.139 | 0.083 | 6.72E-04 | C | G | N | *TMEM16C* |
| rs7101817 | 11 | 26021245 | 0.138 | 0.083 | 6.74E-04 | T | C | N | *TMEM16C* |
| rs704631 | 11 | 131049524 | 0.247 | 0.068 | 6.74E-04 | T | G | N | *HNT* |
| rs6473956 | 8 | 55983159 | 0.267 | 0.066 | 6.75E-04 | G | A | N | *XKR4* |
| rs1485436 | 12 | 30298479 | 0.422 | 0.058 | 6.75E-04 | T | C | N | *IPO8* |
| rs2695811 | 11 | 131052018 | 0.256 | 0.068 | 6.75E-04 | A | G | N | *HNT* |
| rs6929872 | 6 | 170184112 | 0.257 | -0.065 | 6.76E-04 | C | T | Y | *DLL1* |
| rs550495 | 3 | 174693746 | 0.115 | -0.090 | 6.76E-04 | G | A | Y | *NLGN1* |
| rs495244 | 3 | 174693714 | 0.115 | -0.090 | 6.76E-04 | G | T | Y | *NLGN1* |
| rs4814194 | 20 | 12988254 | 0.191 | -0.073 | 6.77E-04 | T | G | Y | *SPTLC3* |
| rs7119696 | 11 | 26021488 | 0.138 | 0.083 | 6.78E-04 | G | C | N | *TMEM16C* |
| rs11926413 | 3 | 73738679 | 0.518 | -0.057 | 6.79E-04 | T | C | Y | *PDZRN3* |
| rs7950045 | 11 | 26024170 | 0.139 | 0.083 | 6.80E-04 | C | T | N | *TMEM16C* |
| rs10889142 | 1 | 59749697 | 0.336 | 0.061 | 6.80E-04 | C | T | Y | *FLJ10986* |
| rs17583935 | 4 | 149927505 | 0.323 | -0.062 | 6.80E-04 | C | T | N | *NR3C2* |
| rs813393 | 20 | 21399139 | 0.088 | -0.106 | 6.81E-04 | A | G | N | *NKX2-2* |
| rs2236437 | 21 | 39478465 | 0.064 | -0.118 | 6.81E-04 | C | T | N | *BRWD1* |
| rs4814183 | 20 | 12972112 | 0.369 | -0.061 | 6.82E-04 | C | A | N | *SPTLC3* |
| rs6569801 | 6 | 132840789 | 0.437 | -0.058 | 6.82E-04 | T | C | N | *STX7* |
| rs2063024 | 11 | 131118832 | 0.207 | 0.072 | 6.83E-04 | A | G | N | *HNT* |
| rs12617354 | 2 | 238342353 | 0.067 | 0.118 | 6.83E-04 | G | A | N | *LRRFIP1* |
| rs1004173 | 6 | 47552976 | 0.271 | -0.068 | 6.84E-04 | T | C | N | *CD2AP* |
| rs9366172 | 6 | 170184308 | 0.257 | -0.065 | 6.84E-04 | A | T | N | *DLL1* |
| rs7571496 | 2 | 6086802 | 0.264 | 0.066 | 6.85E-04 | G | A | N | *SOX11* |
| rs6478106 | 9 | 116585487 | 0.273 | 0.067 | 6.85E-04 | T | C | N | *TNFSF15* |
| rs7084165 | 10 | 17082169 | 0.208 | -0.071 | 6.86E-04 | T | C | N | *CUBN* |
| rs10889143 | 1 | 59749725 | 0.336 | 0.061 | 6.86E-04 | T | C | N | *FLJ10986* |
| rs7105188 | 11 | 26021506 | 0.138 | 0.083 | 6.86E-04 | T | A | Y | *TMEM16C* |
| rs10742134 | 11 | 26040588 | 0.109 | 0.092 | 6.86E-04 | T | A | N | *TMEM16C* |
| rs6603803 | 1 | 1802548 | 0.501 | 0.057 | 6.87E-04 | A | G | Y | *GNB1* |
| rs9366174 | 6 | 170184494 | 0.257 | -0.065 | 6.87E-04 | T | C | N | *DLL1* |
| rs11597179 | 10 | 17081911 | 0.208 | -0.071 | 6.87E-04 | T | G | N | *CUBN* |
| rs4710764 | 6 | 170190039 | 0.257 | -0.065 | 6.87E-04 | G | A | Y | *DLL1* |
| rs2695809 | 11 | 131051304 | 0.256 | 0.068 | 6.88E-04 | A | G | N | *HNT* |
| rs10184151 | 2 | 68151928 | 0.180 | 0.076 | 6.88E-04 | G | C | N | *C1D* |
| rs2373444 | 12 | 100267178 | 0.384 | -0.060 | 6.89E-04 | C | T | N | *UTP20* |
| rs2399789 | 10 | 12280105 | 0.424 | 0.060 | 6.89E-04 | G | C | N | *NUDT5* |
| rs11222764 | 11 | 131118140 | 0.207 | 0.072 | 6.89E-04 | C | T | N | *HNT* |
| rs9660106 | 1 | 1787807 | 0.501 | 0.057 | 6.90E-04 | A | G | N | *GNB1* |
| rs6456175 | 6 | 170190801 | 0.257 | -0.065 | 6.90E-04 | T | G | N | *DLL1* |
| rs9503950 | 6 | 4137134 | 0.133 | 0.085 | 6.91E-04 | C | T | Y | *PECI* |
| rs2051800 | 19 | 41271456 | 0.441 | 0.058 | 6.91E-04 | G | A | N | *WDR62* |
| rs7900486 | 10 | 17080855 | 0.208 | -0.071 | 6.91E-04 | T | C | N | *CUBN* |
| rs11222762 | 11 | 131116683 | 0.207 | 0.072 | 6.92E-04 | G | A | N | *HNT* |
| rs7900377 | 10 | 17080789 | 0.208 | -0.071 | 6.92E-04 | T | C | Y | *CUBN* |
| rs2658825 | 11 | 131050847 | 0.256 | 0.068 | 6.94E-04 | T | C | N | *HNT* |
| rs9807974 | 2 | 68141582 | 0.180 | 0.075 | 6.94E-04 | C | G | N | *C1D* |
| rs6687065 | 1 | 1768329 | 0.500 | 0.057 | 6.94E-04 | A | G | N | *GNB1* |
| rs2293058 | 2 | 170079222 | 0.077 | -0.108 | 6.94E-04 | C | T | N | *KBTBD10* |
| rs1412788 | 1 | 168475049 | 0.342 | -0.061 | 6.95E-04 | G | A | N | *KIFAP3* |
| rs7679262 | 4 | 14106615 | 0.441 | 0.058 | 6.96E-04 | G | A | N | *CPEB2* |
| rs4710763 | 6 | 170190001 | 0.257 | -0.065 | 6.98E-04 | T | C | N | *DLL1* |
| rs10767507 | 11 | 26040766 | 0.109 | 0.092 | 6.98E-04 | T | G | N | *TMEM16C* |
| rs9503979 | 6 | 4157958 | 0.133 | 0.085 | 6.99E-04 | C | T | N | *PECI* |
| rs2180504 | 14 | 51678649 | 0.025 | 0.186 | 7.00E-04 | A | G | Y | *C14orf166* |
| rs6679025 | 1 | 84534335 | 0.014 | -0.251 | 7.00E-04 | A | G | N | *SAMD13* |
| rs789543 | 11 | 131100478 | 0.185 | 0.074 | 7.00E-04 | G | A | Y | *HNT* |
| rs9299995 | 11 | 26024615 | 0.139 | 0.082 | 7.01E-04 | T | C | N | *TMEM16C* |
| rs704629 | 11 | 131100243 | 0.185 | 0.074 | 7.01E-04 | G | A | Y | *HNT* |
| rs171408 | 3 | 8583920 | 0.212 | 0.074 | 7.01E-04 | G | A | N | *LMCD1* |
| rs10439493 | 2 | 68114519 | 0.165 | 0.082 | 7.01E-04 | C | A | N | *C1D* |
| rs9502134 | 6 | 4156533 | 0.133 | 0.085 | 7.02E-04 | G | A | Y | *PECI* |
| rs789541 | 11 | 131099383 | 0.185 | 0.074 | 7.02E-04 | T | G | N | *HNT* |
| rs9426000 | 1 | 34375137 | 0.425 | -0.061 | 7.02E-04 | C | T | N | *CSMD2* |
| rs2242595 | 17 | 18000179 | 0.116 | 0.090 | 7.03E-04 | A | G | Y | *MYO15A* |
| rs1391466 | 8 | 55980283 | 0.267 | 0.065 | 7.04E-04 | A | C | Y | *XKR4* |
| rs7791782 | 7 | 96878667 | 0.045 | -0.141 | 7.04E-04 | A | G | N | *ACN9* |
| rs12465387 | 2 | 170058313 | 0.077 | -0.108 | 7.05E-04 | A | G | Y | *BBS5* |
| rs3812151 | 6 | 80832457 | 0.324 | -0.063 | 7.05E-04 | G | C | N | *TTK* |
| rs10808106 | 7 | 96880051 | 0.045 | -0.141 | 7.06E-04 | T | C | N | *ACN9* |
| rs11015138 | 10 | 26835841 | 0.175 | -0.076 | 7.06E-04 | A | G | N | *APBB1IP* |
| rs6900233 | 6 | 142758976 | 0.213 | 0.070 | 7.06E-04 | G | C | N | *GPR126* |
| rs757223 | 19 | 41266638 | 0.441 | 0.058 | 7.07E-04 | C | T | Y | *WDR62* |
| rs3105605 | 11 | 131097719 | 0.185 | 0.074 | 7.07E-04 | A | G | N | *HNT* |
| rs10770410 | 12 | 18994349 | 0.399 | 0.058 | 7.07E-04 | G | A | N | *PLEKHA5* |
| rs1506663 | 11 | 131096150 | 0.185 | 0.074 | 7.08E-04 | T | C | N | *HNT* |
| rs16856981 | 2 | 170049956 | 0.077 | -0.108 | 7.08E-04 | G | A | N | *BBS5* |
| rs11222761 | 11 | 131114915 | 0.207 | 0.071 | 7.09E-04 | T | G | N | *HNT* |
| rs17521570 | 5 | 34793423 | 0.121 | 0.089 | 7.09E-04 | A | G | Y | *RAI14* |
| rs789553 | 11 | 131095733 | 0.185 | 0.074 | 7.09E-04 | G | A | N | *HNT* |
| rs9519839 | 13 | 105354562 | 0.148 | -0.083 | 7.10E-04 | A | G | N | *DAOA* |
| rs7174876 | 15 | 53406853 | 0.186 | -0.074 | 7.11E-04 | A | G | N | *PIGB* |
| rs7776438 | 6 | 170189808 | 0.257 | -0.065 | 7.11E-04 | A | G | N | *DLL1* |
| rs789552 | 11 | 131095508 | 0.185 | 0.074 | 7.11E-04 | G | T | N | *HNT* |
| rs16823068 | 2 | 170047232 | 0.077 | -0.109 | 7.12E-04 | A | C | N | *BBS5* |
| rs4398807 | 7 | 39238955 | 0.538 | -0.058 | 7.12E-04 | T | G | Y | *POU6F2* |
| rs789550 | 11 | 131094636 | 0.185 | 0.074 | 7.12E-04 | A | C | N | *HNT* |
| rs11071180 | 15 | 53409591 | 0.186 | -0.074 | 7.13E-04 | A | G | N | *PIGB* |
| rs12999941 | 2 | 60433128 | 0.437 | -0.058 | 7.13E-04 | T | C | Y | *BCL11A* |
| rs12232305 | 15 | 50915987 | 0.040 | -0.148 | 7.14E-04 | T | C | N | *ONECUT1* |
| rs17071756 | 6 | 142756888 | 0.213 | 0.070 | 7.14E-04 | C | T | N | *GPR126* |
| rs6143009 | 20 | 60274724 | 0.533 | -0.058 | 7.15E-04 | G | C | N | *OSBPL2* |
| rs10253095 | 7 | 39221184 | 0.540 | -0.058 | 7.15E-04 | G | A | N | *POU6F2* |
| rs11015141 | 10 | 26838469 | 0.175 | -0.076 | 7.16E-04 | T | C | Y | *APBB1IP* |
| rs6935785 | 6 | 132830016 | 0.438 | -0.057 | 7.16E-04 | C | A | Y | *STX7* |
| rs4813428 | 20 | 21399848 | 0.088 | -0.106 | 7.16E-04 | T | C | N | *NKX2-2* |
| rs16856977 | 2 | 170046069 | 0.077 | -0.109 | 7.16E-04 | C | G | N | *BBS5* |
| rs6971177 | 7 | 96868701 | 0.045 | -0.140 | 7.17E-04 | A | G | Y | *ACN9* |
| rs3923506 | 7 | 39227433 | 0.540 | -0.058 | 7.17E-04 | C | T | N | *POU6F2* |
| rs11636687 | 15 | 53392444 | 0.186 | -0.074 | 7.17E-04 | C | T | N | *PIGB* |
| rs231966 | 6 | 155772504 | 0.011 | 0.280 | 7.18E-04 | T | C | Y | *NOX3* |
| rs990630 | 4 | 11259329 | 0.408 | 0.058 | 7.18E-04 | G | A | N | *HS3ST1* |
| rs10894432 | 11 | 131114707 | 0.207 | 0.071 | 7.18E-04 | A | G | Y | *HNT* |
| rs1397423 | 4 | 35799419 | 0.451 | 0.059 | 7.18E-04 | G | A | N | *CENTD1* |
| rs1537482 | 20 | 60278224 | 0.533 | -0.058 | 7.19E-04 | C | G | N | *OSBPL2* |
| rs10743294 | 12 | 18990463 | 0.399 | 0.058 | 7.21E-04 | C | G | Y | *PLEKHA5* |
| rs16856972 | 2 | 170045043 | 0.077 | -0.109 | 7.22E-04 | A | G | N | *BBS5* |
| rs6976613 | 7 | 31922992 | 0.287 | -0.064 | 7.22E-04 | A | G | N | *PDE1C* |
| rs2437831 | 11 | 131966826 | 0.311 | -0.062 | 7.23E-04 | C | T | Y | *OPCML* |
| rs10857237 | 4 | 150165984 | 0.399 | 0.059 | 7.23E-04 | G | A | N | *NR3C2* |
| rs3806503 | 2 | 238341695 | 0.064 | 0.117 | 7.24E-04 | G | A | N | *LRRFIP1* |
| rs231965 | 6 | 155771457 | 0.011 | 0.280 | 7.24E-04 | A | G | N | *NOX3* |
| rs2236525 | 20 | 60282230 | 0.533 | -0.058 | 7.24E-04 | T | C | N | *OSBPL2* |
| rs968919 | 2 | 60433492 | 0.437 | -0.058 | 7.24E-04 | T | C | N | *BCL11A* |
| rs10236201 | 7 | 96866757 | 0.045 | -0.140 | 7.25E-04 | C | T | N | *ACN9* |
| rs7786019 | 7 | 69530814 | 0.052 | 0.129 | 7.26E-04 | A | C | N | *AUTS2* |
| rs10249496 | 7 | 96866360 | 0.045 | -0.140 | 7.27E-04 | G | A | N | *ACN9* |
| rs2311178 | 6 | 170188980 | 0.257 | -0.065 | 7.27E-04 | A | G | N | *DLL1* |
| rs165507 | 10 | 132128731 | 0.263 | 0.066 | 7.27E-04 | T | G | N | *GLRX3* |
| rs6121975 | 20 | 60284974 | 0.468 | 0.058 | 7.28E-04 | A | G | N | *OSBPL2* |
| rs17566551 | 7 | 69529962 | 0.052 | 0.129 | 7.28E-04 | A | G | N | *AUTS2* |
| rs848041 | 14 | 36405339 | 0.407 | 0.060 | 7.28E-04 | T | C | N | *SLC25A21* |
| rs231934 | 6 | 155773892 | 0.011 | 0.281 | 7.29E-04 | C | G | N | *NOX3* |
| rs1860293 | 4 | 11247041 | 0.405 | 0.058 | 7.30E-04 | G | T | N | *HS3ST1* |
| rs9321346 | 6 | 132836597 | 0.438 | -0.057 | 7.30E-04 | G | A | N | *STX7* |
| rs231964 | 6 | 155771288 | 0.011 | 0.280 | 7.30E-04 | A | C | N | *NOX3* |
| rs11759653 | 6 | 142762047 | 0.212 | 0.070 | 7.31E-04 | A | G | N | *GPR126* |
| rs11776252 | 8 | 53497487 | 0.223 | 0.069 | 7.31E-04 | A | T | N | *ST18* |
| rs3787434 | 20 | 60267295 | 0.533 | -0.058 | 7.32E-04 | T | G | N | *OSBPL2* |
| rs11215795 | 11 | 115482463 | 0.226 | 0.068 | 7.32E-04 | C | G | N | *CADM1* |
| rs433961 | 12 | 124439428 | 0.411 | -0.058 | 7.33E-04 | T | C | N | *TMEM132B* |
| rs2429432 | 17 | 58768135 | 0.478 | 0.058 | 7.33E-04 | A | G | N | *TANC2* |
| rs4774760 | 15 | 53376504 | 0.186 | -0.073 | 7.33E-04 | A | T | Y | *RAB27A* |
| rs13116963 | 4 | 145644217 | 0.301 | -0.063 | 7.34E-04 | T | C | N | *HHIP* |
| rs2216034 | 7 | 96862813 | 0.045 | -0.140 | 7.34E-04 | G | A | N | *ACN9* |
| rs10486878 | 7 | 69517850 | 0.052 | 0.129 | 7.34E-04 | G | A | N | *AUTS2* |
| rs1062840 | 20 | 47938873 | 0.098 | 0.098 | 7.35E-04 | T | C | N | *SLC9A8* |
| rs1466748 | 6 | 170188060 | 0.257 | -0.065 | 7.35E-04 | G | T | N | *DLL1* |
| rs4925368 | 20 | 60294339 | 0.468 | 0.058 | 7.35E-04 | T | C | N | *OSBPL2* |
| rs7375384 | 4 | 21335775 | 0.479 | -0.058 | 7.36E-04 | T | C | N | *KCNIP4* |
| rs2410776 | 3 | 153198914 | 0.198 | -0.072 | 7.36E-04 | C | G | Y | *SUCNR1* |
| rs231935 | 6 | 155774784 | 0.011 | 0.281 | 7.37E-04 | C | T | N | *NOX3* |
| rs2297592 | 20 | 60295177 | 0.532 | -0.058 | 7.37E-04 | A | G | N | *OSBPL2* |
| rs2399796 | 10 | 12296565 | 0.386 | -0.060 | 7.38E-04 | A | G | N | *NUDT5* |
| rs16967662 | 13 | 105352545 | 0.147 | -0.081 | 7.38E-04 | A | G | Y | *DAOA* |
| rs17141576 | 7 | 69516471 | 0.052 | 0.129 | 7.38E-04 | T | G | N | *AUTS2* |
| rs1149995 | 10 | 9395307 | 0.430 | -0.058 | 7.38E-04 | G | A | N | *GATA3* |
| rs7702304 | 5 | 96057851 | 0.110 | -0.093 | 7.39E-04 | A | G | N | *CAST* |
| rs7794910 | 7 | 96861567 | 0.045 | -0.140 | 7.39E-04 | A | G | N | *ACN9* |
| rs4130405 | 8 | 99489951 | 0.148 | -0.081 | 7.41E-04 | C | A | Y | *KCNS2* |
| rs1906841 | 11 | 26025122 | 0.139 | 0.082 | 7.42E-04 | T | C | Y | *TMEM16C* |
| rs2379130 | 20 | 60300905 | 0.532 | -0.058 | 7.42E-04 | G | A | N | *OSBPL2* |
| rs13232442 | 7 | 31809538 | 0.186 | -0.076 | 7.42E-04 | A | T | N | *PDE1C* |
| rs1075702 | 11 | 86138828 | 0.255 | -0.066 | 7.42E-04 | A | G | N | *PRSS23* |
| rs2614176 | 11 | 37079412 | 0.111 | -0.091 | 7.43E-04 | G | A | N | *C11orf74* |
| rs325095 | 12 | 124451257 | 0.402 | -0.058 | 7.43E-04 | T | C | Y | *TMEM132B* |
| rs345800 | 15 | 31169949 | 0.354 | -0.059 | 7.43E-04 | G | A | Y | *FMN1* |
| rs7224059 | 17 | 58518479 | 0.494 | 0.057 | 7.44E-04 | A | C | N | *TANC2* |
| rs6504149 | 17 | 58521206 | 0.494 | 0.057 | 7.44E-04 | C | G | N | *TANC2* |
| rs12701159 | 7 | 31924539 | 0.287 | -0.064 | 7.45E-04 | T | C | N | *PDE1C* |
| rs2438130 | 15 | 44517472 | 0.315 | 0.061 | 7.45E-04 | T | C | N | *SQRDL* |
| rs9683403 | 4 | 149898860 | 0.332 | -0.061 | 7.46E-04 | A | G | N | *NR3C2* |
| rs764005 | 4 | 150127583 | 0.387 | 0.060 | 7.47E-04 | A | G | N | *NR3C2* |
| rs725853 | 11 | 26013191 | 0.139 | 0.082 | 7.48E-04 | T | C | N | *TMEM16C* |
| rs7300418 | 12 | 115469447 | 0.060 | -0.122 | 7.48E-04 | G | C | N | *FLJ42957* |
| rs10767508 | 11 | 26040783 | 0.109 | 0.092 | 7.49E-04 | G | A | N | *TMEM16C* |
| rs6082455 | 20 | 21500504 | 0.356 | -0.060 | 7.49E-04 | G | A | Y | *NKX2-2* |
| rs10275742 | 7 | 96860046 | 0.045 | -0.140 | 7.50E-04 | T | C | N | *ACN9* |
| rs9309325 | 2 | 60434335 | 0.440 | -0.057 | 7.51E-04 | G | A | Y | *BCL11A* |
| rs12585559 | 13 | 105352259 | 0.147 | -0.081 | 7.51E-04 | C | A | Y | *DAOA* |
| rs1086221 | 11 | 131093052 | 0.184 | 0.074 | 7.51E-04 | T | C | N | *HNT* |
| rs6082981 | 20 | 22959278 | 0.424 | 0.058 | 7.52E-04 | C | T | N | *SSTR4* |
| rs1086220 | 11 | 131091890 | 0.184 | 0.074 | 7.52E-04 | A | G | N | *HNT* |
| rs1653243 | 15 | 44519110 | 0.315 | 0.061 | 7.52E-04 | G | T | N | *SQRDL* |
| rs428215 | 11 | 37082765 | 0.111 | -0.091 | 7.53E-04 | C | T | Y | *C11orf74* |
| rs10862882 | 12 | 83442455 | 0.411 | 0.059 | 7.53E-04 | T | C | N | *SLC6A15* |
| rs1410521 | 6 | 80838324 | 0.324 | -0.062 | 7.54E-04 | G | A | N | *TTK* |
| rs2865793 | 20 | 58232796 | 0.222 | -0.071 | 7.54E-04 | G | T | N | *C20orf197* |
| rs1739584 | 20 | 60242061 | 0.071 | -0.112 | 7.54E-04 | A | G | N | *OSBPL2* |
| rs16967660 | 13 | 105352358 | 0.147 | -0.081 | 7.55E-04 | G | A | Y | *DAOA* |
| rs1086219 | 11 | 131091816 | 0.184 | 0.074 | 7.55E-04 | A | C | N | *HNT* |
| rs2034237 | 5 | 54027024 | 0.148 | -0.080 | 7.56E-04 | A | C | Y | *SNAG1* |
| rs231936 | 6 | 155775314 | 0.011 | 0.282 | 7.56E-04 | A | G | N | *NOX3* |
| rs11115687 | 12 | 82198838 | 0.112 | 0.090 | 7.57E-04 | T | G | N | *TMTC2* |
| rs17614940 | 16 | 46621256 | 0.069 | -0.112 | 7.57E-04 | G | A | Y | *ABCC12* |
| rs796873 | 11 | 131091392 | 0.184 | 0.074 | 7.58E-04 | T | C | N | *HNT* |
| rs1484192 | 15 | 44495566 | 0.315 | 0.061 | 7.58E-04 | T | C | N | *SQRDL* |
| rs1886617 | 10 | 9397549 | 0.403 | -0.059 | 7.58E-04 | A | C | N | *GATA3* |
| rs7641488 | 3 | 73748936 | 0.483 | -0.058 | 7.58E-04 | T | C | N | *PDZRN3* |
| rs6100796 | 20 | 58234268 | 0.222 | -0.071 | 7.58E-04 | T | C | N | *C20orf197* |
| rs1197382 | 14 | 80216529 | 0.102 | 0.095 | 7.58E-04 | C | T | N | *C14orf145* |
| rs7958175 | 12 | 115946321 | 0.018 | -0.215 | 7.59E-04 | T | C | N | *FBXW8* |
| rs7580181 | 2 | 164703093 | 0.176 | 0.078 | 7.59E-04 | T | C | N | *GRB14* |
| rs1848082 | 11 | 131090196 | 0.184 | 0.074 | 7.59E-04 | A | C | N | *HNT* |
| rs1906843 | 11 | 26025402 | 0.139 | 0.082 | 7.59E-04 | G | T | N | *TMEM16C* |
| rs9320788 | 6 | 121362974 | 0.209 | 0.070 | 7.59E-04 | T | C | N | *C6orf170* |
| rs2581810 | 3 | 52935899 | 0.233 | 0.067 | 7.59E-04 | C | T | N | *SFMBT1* |
| rs1000104 | 6 | 170195237 | 0.257 | -0.067 | 7.60E-04 | T | G | N | *DLL1* |
| rs2132307 | 6 | 170194142 | 0.257 | -0.067 | 7.60E-04 | C | T | N | *DLL1* |
| rs1410522 | 6 | 80838511 | 0.323 | -0.062 | 7.60E-04 | A | T | N | *TTK* |
| rs9829694 | 3 | 80432352 | 0.488 | 0.059 | 7.60E-04 | A | G | N | *ROBO1* |
| rs4627118 | 11 | 110423599 | 0.352 | 0.062 | 7.60E-04 | A | T | N | *C11orf53* |
| rs10251859 | 7 | 69501039 | 0.052 | 0.129 | 7.61E-04 | A | G | N | *AUTS2* |
| rs1555331 | 20 | 25196854 | 0.101 | 0.098 | 7.61E-04 | C | G | N | *PYGB* |
| rs3749991 | 6 | 170196193 | 0.257 | -0.067 | 7.62E-04 | G | A | N | *DLL1* |
| rs7719534 | 5 | 166837622 | 0.217 | -0.069 | 7.63E-04 | A | G | N | *ODZ2* |
| rs12634318 | 3 | 176569238 | 0.342 | -0.063 | 7.63E-04 | C | T | N | *NAALADL2* |
| rs12579299 | 12 | 93966373 | 0.194 | -0.072 | 7.63E-04 | G | A | Y | *NR2C1* |
| rs12504241 | 4 | 27148862 | 0.166 | 0.076 | 7.63E-04 | G | A | N | *STIM2* |
| rs17651808 | 15 | 50659613 | 0.097 | 0.097 | 7.64E-04 | A | G | N | *KIAA1370* |
| rs7107646 | 11 | 131962299 | 0.312 | -0.062 | 7.65E-04 | G | A | N | *OPCML* |
| rs2512875 | 11 | 131087468 | 0.184 | 0.074 | 7.65E-04 | C | A | N | *HNT* |
| rs1512103 | 4 | 25630125 | 0.198 | 0.074 | 7.65E-04 | T | C | N | *KIAA0746* |
| rs765654 | 15 | 44496261 | 0.315 | 0.061 | 7.65E-04 | G | C | N | *SQRDL* |
| rs350052 | 5 | 40290787 | 0.186 | 0.073 | 7.65E-04 | C | A | N | *PTGER4* |
| rs2505949 | 6 | 80861133 | 0.378 | -0.059 | 7.65E-04 | G | A | Y | *BCKDHB* |
| rs2474762 | 20 | 25189083 | 0.101 | 0.098 | 7.66E-04 | A | G | N | *PYGB* |
| rs7719299 | 5 | 166837738 | 0.217 | -0.069 | 7.67E-04 | G | A | Y | *ODZ2* |
| rs1512104 | 4 | 25630408 | 0.198 | 0.074 | 7.67E-04 | A | G | N | *KIAA0746* |
| rs350050 | 5 | 40289659 | 0.186 | 0.073 | 7.67E-04 | C | G | Y | *PTGER4* |
| rs12346322 | 9 | 79372214 | 0.087 | 0.106 | 7.67E-04 | C | A | N | *GNA14* |
| rs789539 | 11 | 131083311 | 0.184 | 0.074 | 7.68E-04 | C | T | N | *HNT* |
| rs7755171 | 6 | 121363704 | 0.209 | 0.070 | 7.68E-04 | A | G | N | *C6orf170* |
| rs11115688 | 12 | 82198905 | 0.112 | 0.090 | 7.68E-04 | A | G | N | *TMTC2* |
| rs1406977 | 6 | 88941540 | 0.216 | 0.072 | 7.69E-04 | C | T | N | *CNR1* |
| rs4305304 | 2 | 22916437 | 0.456 | 0.057 | 7.69E-04 | G | C | Y | *ATAD2B* |
| rs9351137 | 6 | 88940725 | 0.216 | 0.072 | 7.70E-04 | A | G | N | *CNR1* |
| rs1086217 | 11 | 131080365 | 0.184 | 0.074 | 7.70E-04 | C | T | N | *HNT* |
| rs2223041 | 21 | 15344474 | 0.352 | -0.062 | 7.71E-04 | T | C | N | *NRIP1* |
| rs789528 | 11 | 131080068 | 0.184 | 0.074 | 7.72E-04 | G | C | N | *HNT* |
| rs765653 | 15 | 44496529 | 0.315 | 0.061 | 7.73E-04 | G | A | N | *SQRDL* |
| rs17685056 | 4 | 27151213 | 0.166 | 0.076 | 7.74E-04 | G | A | N | *STIM2* |
| rs789525 | 11 | 131079109 | 0.184 | 0.074 | 7.74E-04 | C | T | N | *HNT* |
| rs2581830 | 3 | 53109138 | 0.416 | -0.058 | 7.74E-04 | T | C | Y | *RFT1* |
| rs648710 | 11 | 125681586 | 0.256 | 0.066 | 7.74E-04 | A | T | N | *DCPS* |
| rs8078424 | 17 | 55305247 | 0.265 | 0.064 | 7.74E-04 | G | A | N | *TUBD1* |
| rs6817272 | 4 | 11260371 | 0.381 | 0.060 | 7.75E-04 | A | G | N | *HS3ST1* |
| rs17542881 | 15 | 50736797 | 0.092 | 0.099 | 7.75E-04 | C | T | Y | *KIAA1370* |
| rs789524 | 11 | 131078797 | 0.184 | 0.074 | 7.76E-04 | C | A | Y | *HNT* |
| rs11774132 | 8 | 87715555 | 0.084 | -0.106 | 7.76E-04 | C | T | N | *CNGB3* |
| rs7663826 | 4 | 11242666 | 0.356 | 0.060 | 7.77E-04 | T | C | N | *HS3ST1* |
| rs7951037 | 11 | 92345823 | 0.435 | -0.058 | 7.77E-04 | G | A | Y | *MTNR1B* |
| rs6448800 | 4 | 11242680 | 0.356 | 0.060 | 7.77E-04 | A | G | N | *HS3ST1* |
| rs13106661 | 4 | 11242553 | 0.356 | 0.060 | 7.77E-04 | A | C | N | *HS3ST1* |
| rs6569171 | 6 | 121364230 | 0.209 | 0.070 | 7.77E-04 | G | A | N | *C6orf170* |
| rs3912874 | 10 | 66241463 | 0.164 | 0.078 | 7.78E-04 | T | C | Y | *CTNNA3* |
| rs7665516 | 4 | 11243690 | 0.356 | 0.060 | 7.78E-04 | A | G | Y | *HS3ST1* |
| rs11858454 | 15 | 69456169 | 0.360 | -0.059 | 7.79E-04 | A | G | Y | *LOC645296* |
| rs9291501 | 4 | 27145749 | 0.166 | 0.076 | 7.79E-04 | G | A | N | *STIM2* |
| rs10946495 | 6 | 22107279 | 0.380 | 0.059 | 7.79E-04 | C | T | Y | *PRL* |
| rs966240 | 4 | 11251632 | 0.405 | 0.058 | 7.79E-04 | C | T | N | *HS3ST1* |
| rs9975379 | 21 | 28847269 | 0.124 | -0.090 | 7.79E-04 | A | T | N | *N6AMT1* |
| rs1411860 | 9 | 95803753 | 0.515 | -0.057 | 7.80E-04 | G | T | N | *PTPDC1* |
| rs17449605 | 3 | 80092899 | 0.288 | -0.066 | 7.81E-04 | G | A | N | *ROBO1* |
| rs9905543 | 17 | 55312624 | 0.265 | 0.064 | 7.81E-04 | C | T | N | *TUBD1* |
| rs6903278 | 6 | 121364341 | 0.209 | 0.070 | 7.81E-04 | G | T | N | *C6orf170* |
| rs9790802 | 4 | 27145627 | 0.166 | 0.076 | 7.82E-04 | G | T | N | *STIM2* |
| rs4261605 | 17 | 58545061 | 0.493 | 0.057 | 7.82E-04 | T | C | N | *TANC2* |
| rs7821970 | 8 | 55977877 | 0.267 | 0.064 | 7.82E-04 | T | C | N | *XKR4* |
| rs7271202 | 20 | 58235038 | 0.223 | -0.071 | 7.83E-04 | A | G | N | *C20orf197* |
| rs17054868 | 13 | 57672932 | 0.445 | -0.057 | 7.83E-04 | T | C | N | *PCDH17* |
| rs1928167 | 6 | 22128045 | 0.463 | 0.057 | 7.83E-04 | T | C | N | *PRL* |
| rs789523 | 11 | 131078542 | 0.184 | 0.074 | 7.84E-04 | T | C | N | *HNT* |
| rs7757571 | 6 | 142744282 | 0.212 | 0.070 | 7.84E-04 | C | A | N | *GPR126* |
| rs16892087 | 6 | 9421263 | 0.096 | -0.096 | 7.85E-04 | T | C | Y | *OFCC1* |
| rs12308333 | 12 | 18991105 | 0.083 | 0.103 | 7.85E-04 | G | T | Y | *PLEKHA5* |
| rs9291500 | 4 | 27145506 | 0.166 | 0.076 | 7.86E-04 | T | A | N | *STIM2* |
| rs247983 | 3 | 174653917 | 0.119 | -0.088 | 7.86E-04 | T | C | N | *NLGN1* |
| rs10279061 | 7 | 96857231 | 0.045 | -0.140 | 7.87E-04 | G | T | N | *ACN9* |
| rs10862618 | 12 | 82199166 | 0.112 | 0.090 | 7.87E-04 | T | C | N | *TMTC2* |
| rs12213892 | 6 | 142743927 | 0.212 | 0.070 | 7.87E-04 | G | A | N | *GPR126* |
| rs16914904 | 12 | 18991369 | 0.083 | 0.103 | 7.88E-04 | C | T | N | *PLEKHA5* |
| rs9975189 | 21 | 28847842 | 0.124 | -0.090 | 7.88E-04 | G | A | N | *N6AMT1* |
| rs9291499 | 4 | 27145486 | 0.166 | 0.076 | 7.88E-04 | A | G | N | *STIM2* |
| rs10771657 | 12 | 30250561 | 0.438 | 0.058 | 7.88E-04 | T | C | Y | *TMTC1* |
| rs348609 | 5 | 40296686 | 0.186 | 0.073 | 7.88E-04 | A | G | N | *PTGER4* |
| rs9889828 | 17 | 58859232 | 0.453 | 0.058 | 7.88E-04 | T | C | N | *TANC2* |
| rs239360 | 17 | 58505762 | 0.489 | 0.058 | 7.89E-04 | T | G | N | *TANC2* |
| rs249842 | 12 | 97395488 | 0.190 | 0.073 | 7.89E-04 | C | T | Y | *TMPO* |
| rs7204072 | 16 | 12389761 | 0.150 | 0.082 | 7.89E-04 | A | T | N | *LOC92017* |
| rs11070520 | 15 | 44496697 | 0.315 | 0.061 | 7.89E-04 | A | C | N | *SQRDL* |
| rs7262838 | 20 | 58251965 | 0.218 | -0.072 | 7.89E-04 | G | T | N | *C20orf197* |
| rs9977893 | 21 | 28848119 | 0.123 | -0.090 | 7.89E-04 | A | G | N | *N6AMT1* |
| rs164317 | 5 | 34795220 | 0.122 | 0.088 | 7.90E-04 | A | G | N | *RAI14* |
| rs10758199 | 9 | 33206016 | 0.375 | 0.060 | 7.90E-04 | T | C | N | *SPINK4* |
| rs12231742 | 12 | 30249763 | 0.438 | 0.058 | 7.90E-04 | A | G | N | *TMTC1* |
| rs10220224 | 13 | 57674201 | 0.445 | -0.057 | 7.90E-04 | C | T | N | *PCDH17* |
| rs4771525 | 13 | 87736628 | 0.327 | 0.064 | 7.90E-04 | A | G | N | *SLITRK5* |
| rs1641882 | 16 | 12390290 | 0.150 | 0.082 | 7.91E-04 | C | A | Y | *LOC92017* |
| rs1880628 | 7 | 45413414 | 0.226 | -0.068 | 7.91E-04 | C | T | N | *ADCY1* |
| rs789522 | 11 | 131077753 | 0.184 | 0.074 | 7.91E-04 | C | T | N | *HNT* |
| rs11215797 | 11 | 115483990 | 0.218 | 0.069 | 7.92E-04 | C | T | N | *CADM1* |
| rs7603599 | 2 | 164702899 | 0.176 | 0.078 | 7.93E-04 | G | A | N | *GRB14* |
| rs9392573 | 6 | 425628 | 0.195 | 0.072 | 7.93E-04 | A | G | Y | *EXOC2* |
| rs7130121 | 11 | 131960741 | 0.325 | -0.061 | 7.93E-04 | T | C | N | *OPCML* |
| rs7209974 | 17 | 58542057 | 0.493 | 0.057 | 7.94E-04 | C | G | N | *TANC2* |
| rs9790659 | 4 | 27145119 | 0.166 | 0.076 | 7.94E-04 | A | C | N | *STIM2* |
| rs7775551 | 6 | 17155652 | 0.137 | -0.087 | 7.95E-04 | A | G | N | *RBM24* |
| rs11853359 | 15 | 69408578 | 0.319 | -0.061 | 7.96E-04 | A | G | N | *LOC645296* |
| rs8071475 | 17 | 55328702 | 0.265 | 0.064 | 7.96E-04 | C | T | N | *RPS6KB1* |
| rs7212556 | 17 | 58537672 | 0.494 | 0.057 | 7.96E-04 | T | G | N | *TANC2* |
| rs16967650 | 13 | 105351058 | 0.147 | -0.081 | 7.97E-04 | A | C | N | *DAOA* |
| rs7349615 | 4 | 27144546 | 0.166 | 0.076 | 7.98E-04 | G | A | N | *STIM2* |
| rs10268743 | 7 | 45409924 | 0.226 | -0.068 | 7.98E-04 | C | T | N | *ADCY1* |
| rs1848083 | 11 | 131076956 | 0.184 | 0.074 | 7.99E-04 | A | T | N | *HNT* |
| rs2413809 | 15 | 44497210 | 0.315 | 0.061 | 7.99E-04 | G | C | N | *SQRDL* |
| rs7258010 | 19 | 61597724 | 0.488 | -0.059 | 8.00E-04 | A | G | N | *ZNF582* |
| rs1331218 | 9 | 95806242 | 0.484 | 0.057 | 8.00E-04 | T | C | N | *PTPDC1* |
| rs17158546 | 7 | 29953269 | 0.017 | -0.222 | 8.00E-04 | G | A | N | *SCRN1* |
| rs247969 | 3 | 174657727 | 0.119 | -0.088 | 8.00E-04 | G | T | N | *NLGN1* |
| rs9994945 | 4 | 21405941 | 0.514 | 0.057 | 8.00E-04 | A | C | N | *KCNIP4* |
| rs7501430 | 17 | 58530859 | 0.494 | 0.057 | 8.00E-04 | T | C | N | *TANC2* |
| rs611151 | 11 | 125693676 | 0.252 | 0.067 | 8.00E-04 | T | C | N | *DCPS* |
| rs13203325 | 6 | 82839184 | 0.449 | 0.060 | 8.01E-04 | A | G | N | *IBTK* |
| rs2604169 | 15 | 31175322 | 0.352 | -0.060 | 8.01E-04 | G | A | N | *FMN1* |
| rs11647884 | 16 | 79845000 | 0.220 | -0.072 | 8.02E-04 | C | T | N | *BCMO1* |
| rs6733843 | 2 | 75903289 | 0.139 | 0.082 | 8.03E-04 | T | C | Y | *C2orf3* |
| rs9316977 | 13 | 57663532 | 0.445 | -0.057 | 8.04E-04 | A | G | N | *PCDH17* |
| rs6903424 | 6 | 142741641 | 0.212 | 0.070 | 8.04E-04 | C | A | N | *GPR126* |
| rs4385051 | 4 | 27144169 | 0.165 | 0.076 | 8.05E-04 | T | C | N | *STIM2* |
| rs1993423 | 4 | 155043562 | 0.447 | 0.058 | 8.05E-04 | T | C | N | *SFRP2* |
| rs2413810 | 15 | 44497250 | 0.315 | 0.061 | 8.06E-04 | T | C | N | *SQRDL* |
| rs9505942 | 6 | 169333718 | 0.151 | -0.082 | 8.06E-04 | C | T | N | *THBS2* |
| rs2658863 | 11 | 131076610 | 0.184 | 0.074 | 8.07E-04 | C | G | N | *HNT* |
| rs4234989 | 4 | 27144087 | 0.165 | 0.076 | 8.09E-04 | A | G | N | *STIM2* |
| rs307387 | 10 | 132133183 | 0.253 | 0.067 | 8.10E-04 | T | C | N | *GLRX3* |
| rs1993425 | 4 | 155043952 | 0.447 | 0.058 | 8.10E-04 | A | G | Y | *SFRP2* |
| rs1009381 | 19 | 41229799 | 0.406 | 0.057 | 8.10E-04 | G | A | N | *THAP8* |
| rs8062640 | 16 | 82493533 | 0.093 | -0.097 | 8.11E-04 | G | A | Y | *MLYCD* |
| rs10778958 | 12 | 82199274 | 0.111 | 0.091 | 8.12E-04 | T | A | N | *TMTC2* |
| rs2297257 | 21 | 39484035 | 0.064 | -0.117 | 8.13E-04 | A | T | N | *BRWD1* |
| rs4234988 | 4 | 27144026 | 0.165 | 0.076 | 8.13E-04 | C | T | N | *STIM2* |
| rs12659815 | 5 | 4128178 | 0.294 | -0.063 | 8.14E-04 | A | G | N | *IRX1* |
| rs1032732 | 4 | 14078513 | 0.466 | 0.058 | 8.14E-04 | T | G | N | *CPEB2* |
| rs10778961 | 12 | 82199826 | 0.111 | 0.091 | 8.14E-04 | T | A | N | *TMTC2* |
| rs1111483 | 16 | 52558408 | 0.469 | 0.057 | 8.15E-04 | C | A | Y | *FTO* |
| rs11641446 | 16 | 79845438 | 0.220 | -0.072 | 8.16E-04 | A | G | N | *BCMO1* |
| rs11012811 | 10 | 18478462 | 0.315 | -0.063 | 8.17E-04 | T | G | N | *CACNB2* |
| rs3087776 | 17 | 58864010 | 0.453 | 0.058 | 8.17E-04 | T | C | N | *CYB561* |
| rs17718377 | 9 | 4844472 | 0.257 | -0.065 | 8.17E-04 | G | C | N | *RCL1* |
| rs6682884 | 1 | 55246913 | 0.415 | -0.058 | 8.17E-04 | C | A | Y | *BSND* |
| rs6922607 | 6 | 142745176 | 0.212 | 0.070 | 8.17E-04 | G | A | N | *GPR126* |
| rs1344502 | 16 | 52558293 | 0.469 | 0.057 | 8.17E-04 | G | A | N | *FTO* |
| rs9605447 | 22 | 16814399 | 0.024 | 0.189 | 8.18E-04 | C | T | N | *MICAL3* |
| rs8043034 | 15 | 37356627 | 0.034 | 0.159 | 8.18E-04 | C | A | N | *FLJ39531* |
| rs2695834 | 11 | 131076235 | 0.184 | 0.074 | 8.18E-04 | C | A | N | *HNT* |
| rs4750539 | 10 | 14703191 | 0.101 | 0.098 | 8.18E-04 | A | T | N | *FAM107B* |
| rs417436 | 12 | 124442885 | 0.405 | -0.057 | 8.19E-04 | A | C | N | *TMEM132B* |
| rs1905556 | 15 | 44498281 | 0.315 | 0.061 | 8.19E-04 | G | A | N | *SQRDL* |
| rs11115690 | 12 | 82200580 | 0.111 | 0.091 | 8.20E-04 | C | T | N | *TMTC2* |
| rs2358166 | 6 | 121372082 | 0.209 | 0.070 | 8.20E-04 | T | G | N | *C6orf170* |
| rs1375758 | 4 | 27143877 | 0.165 | 0.076 | 8.20E-04 | C | T | N | *STIM2* |
| rs348615 | 5 | 40310881 | 0.186 | 0.073 | 8.21E-04 | G | A | Y | *PTGER4* |
| rs17046388 | 2 | 117293609 | 0.165 | -0.077 | 8.21E-04 | T | G | Y | *DPP10* |
| rs11151800 | 18 | 68608275 | 0.397 | -0.059 | 8.22E-04 | A | G | N | *NETO1* |
| rs4875520 | 8 | 5196363 | 0.338 | 0.060 | 8.23E-04 | G | C | N | *CSMD1* |
| rs1385079 | 15 | 44498561 | 0.315 | 0.061 | 8.23E-04 | T | C | N | *SQRDL* |
| rs6876062 | 5 | 120253656 | 0.409 | -0.058 | 8.23E-04 | A | G | N | *PRR16* |
| rs2410104 | 21 | 39486334 | 0.064 | -0.117 | 8.24E-04 | T | C | N | *BRWD1* |
| rs2286569 | 17 | 58871737 | 0.453 | 0.058 | 8.24E-04 | C | T | N | *CYB561* |
| rs12659390 | 5 | 54025314 | 0.153 | -0.080 | 8.24E-04 | C | T | N | *SNAG1* |
| rs418105 | 12 | 124443445 | 0.405 | -0.057 | 8.24E-04 | T | C | N | *TMEM132B* |
| rs10780945 | 9 | 72348716 | 0.508 | 0.058 | 8.26E-04 | A | T | N | *TRPM3* |
| rs6028264 | 20 | 37136708 | 0.123 | 0.086 | 8.26E-04 | C | T | N | *DHX35* |
| rs936729 | 18 | 68610309 | 0.397 | -0.059 | 8.26E-04 | C | T | N | *NETO1* |
| rs7696696 | 4 | 185643632 | 0.241 | -0.069 | 8.26E-04 | G | A | N | *IRF2* |
| rs6539721 | 12 | 82201950 | 0.111 | 0.091 | 8.26E-04 | T | C | N | *TMTC2* |
| rs2658862 | 11 | 131076004 | 0.184 | 0.073 | 8.26E-04 | A | G | N | *HNT* |
| rs11050753 | 12 | 30303065 | 0.400 | 0.059 | 8.27E-04 | T | A | N | *IPO8* |
| rs6900087 | 6 | 142758996 | 0.211 | 0.070 | 8.28E-04 | T | A | N | *GPR126* |
| rs10122795 | 9 | 98020181 | 0.266 | -0.064 | 8.28E-04 | T | C | N | *HSD17B3* |
| rs3006651 | 10 | 32460380 | 0.369 | 0.060 | 8.28E-04 | G | A | N | *KIF5B* |
| rs754071 | 5 | 121890106 | 0.225 | -0.068 | 8.29E-04 | T | C | Y | *SNCAIP* |
| rs1878161 | 6 | 121372360 | 0.209 | 0.070 | 8.30E-04 | A | G | N | *C6orf170* |
| rs4778639 | 15 | 79387506 | 0.081 | 0.105 | 8.31E-04 | G | T | N | *STARD5* |
| rs11727583 | 4 | 145652452 | 0.300 | -0.062 | 8.31E-04 | A | G | N | *HHIP* |
| rs11075183 | 16 | 13154287 | 0.444 | 0.058 | 8.31E-04 | C | T | N | *FLJ11151* |
| rs920601 | 1 | 29994884 | 0.445 | 0.058 | 8.32E-04 | G | A | N | *PTPRU* |
| rs13224399 | 7 | 48799846 | 0.077 | -0.108 | 8.32E-04 | A | G | N | *ABCA13* |
| rs13308932 | 7 | 48803331 | 0.077 | -0.108 | 8.32E-04 | A | G | N | *ABCA13* |
| rs7350847 | 16 | 56545987 | 0.167 | -0.079 | 8.32E-04 | T | C | N | *CNGB1* |
| rs10778963 | 12 | 82203026 | 0.111 | 0.091 | 8.33E-04 | T | C | N | *TMTC2* |
| rs9374994 | 6 | 121374075 | 0.209 | 0.070 | 8.33E-04 | T | C | N | *C6orf170* |
| rs11083294 | 18 | 24461420 | 0.260 | 0.065 | 8.34E-04 | A | T | N | *CDH2* |
| rs1485780 | 6 | 47664589 | 0.267 | -0.064 | 8.34E-04 | C | A | N | *CD2AP* |
| rs6444193 | 3 | 188216882 | 0.224 | -0.068 | 8.35E-04 | G | A | N | *ST6GAL1* |
| rs4577037 | 15 | 79383715 | 0.081 | 0.104 | 8.35E-04 | G | T | N | *STARD5* |
| rs17684939 | 4 | 27141849 | 0.165 | 0.076 | 8.35E-04 | G | C | Y | *STIM2* |
| rs12509051 | 4 | 27140932 | 0.165 | 0.076 | 8.37E-04 | A | C | N | *STIM2* |
| rs1827608 | 18 | 24462509 | 0.260 | 0.065 | 8.37E-04 | A | G | Y | *CDH2* |
| rs2581801 | 3 | 52947947 | 0.233 | 0.067 | 8.37E-04 | G | T | N | *SFMBT1* |
| rs1387485 | 11 | 26012743 | 0.140 | 0.081 | 8.38E-04 | T | C | N | *TMEM16C* |
| rs2440354 | 15 | 50925436 | 0.040 | -0.145 | 8.38E-04 | C | T | Y | *ONECUT1* |
| rs16844275 | 2 | 160312625 | 0.033 | 0.158 | 8.38E-04 | T | C | Y | *7-Mar* |
| rs2658824 | 11 | 131075168 | 0.184 | 0.073 | 8.39E-04 | C | T | N | *HNT* |
| rs920600 | 1 | 29995112 | 0.445 | 0.058 | 8.39E-04 | A | G | N | *PTPRU* |
| rs4778636 | 15 | 79378694 | 0.081 | 0.104 | 8.39E-04 | A | G | Y | *IL16* |
| rs13428140 | 2 | 75910951 | 0.140 | 0.082 | 8.39E-04 | C | G | N | *C2orf3* |
| rs2357528 | 2 | 160302773 | 0.033 | 0.158 | 8.39E-04 | G | A | N | *7-Mar* |
| rs4557646 | 7 | 48821333 | 0.076 | -0.109 | 8.40E-04 | T | C | N | *ABCA13* |
| rs9401362 | 6 | 121374089 | 0.209 | 0.070 | 8.40E-04 | A | T | N | *C6orf170* |
| rs1825526 | 12 | 30295901 | 0.428 | 0.057 | 8.40E-04 | A | C | N | *IPO8* |
| rs1566460 | 16 | 83574476 | 0.253 | -0.068 | 8.40E-04 | T | C | N | *ZDHHC7* |
| rs9845479 | 3 | 60540185 | 0.341 | -0.063 | 8.41E-04 | T | C | N | *FHIT* |
| rs974541 | 4 | 61803943 | 0.240 | -0.067 | 8.41E-04 | A | G | Y | *LPHN3* |
| rs10774871 | 12 | 115467105 | 0.067 | -0.114 | 8.42E-04 | G | A | Y | *FLJ42957* |
| rs6971594 | 7 | 48822608 | 0.076 | -0.109 | 8.42E-04 | G | A | N | *ABCA13* |
| rs2479825 | 9 | 98055652 | 0.264 | -0.067 | 8.43E-04 | G | A | N | *HSD17B3* |
| rs2066475 | 9 | 98055080 | 0.264 | -0.066 | 8.43E-04 | T | C | N | *HSD17B3* |
| rs10422055 | 19 | 41271148 | 0.406 | 0.056 | 8.44E-04 | A | G | N | *WDR62* |
| rs17019408 | 4 | 145652902 | 0.299 | -0.062 | 8.44E-04 | A | C | Y | *HHIP* |
| rs1014165 | 4 | 14076576 | 0.466 | 0.058 | 8.44E-04 | T | C | N | *CPEB2* |
| rs2695833 | 11 | 131073585 | 0.184 | 0.073 | 8.44E-04 | T | C | N | *HNT* |
| rs4421134 | 5 | 120271601 | 0.408 | -0.058 | 8.45E-04 | A | G | N | *PRR16* |
| rs17783909 | 16 | 82502647 | 0.094 | -0.102 | 8.48E-04 | G | C | N | *MLYCD* |
| rs12068365 | 1 | 149282492 | 0.076 | 0.107 | 8.49E-04 | A | G | Y | *BNIPL* |
| rs16987791 | 20 | 37137226 | 0.123 | 0.085 | 8.50E-04 | G | A | Y | *DHX35* |
| rs7578485 | 2 | 229451253 | 0.228 | 0.068 | 8.50E-04 | A | G | Y | *PID1* |
| rs4895287 | 5 | 120272394 | 0.408 | -0.058 | 8.51E-04 | T | C | N | *PRR16* |
| rs2170226 | 9 | 115503981 | 0.186 | 0.073 | 8.52E-04 | T | C | N | *RGS3* |
| rs13160133 | 5 | 43261391 | 0.063 | 0.122 | 8.52E-04 | G | C | N | *MGC42105* |
| rs765119 | 12 | 30300567 | 0.396 | 0.058 | 8.53E-04 | A | C | Y | *IPO8* |
| rs12185747 | 2 | 229453078 | 0.228 | 0.068 | 8.55E-04 | G | A | N | *PID1* |
| rs10484733 | 6 | 142752681 | 0.211 | 0.069 | 8.55E-04 | G | C | N | *GPR126* |
| rs348617 | 5 | 40316826 | 0.186 | 0.073 | 8.56E-04 | A | G | N | *PTGER4* |
| rs1827591 | 4 | 21341284 | 0.486 | -0.058 | 8.56E-04 | C | T | N | *KCNIP4* |
| rs163827 | 5 | 14168074 | 0.498 | 0.057 | 8.57E-04 | C | G | Y | *TRIO* |
| rs12522188 | 5 | 90961714 | 0.503 | 0.056 | 8.58E-04 | C | T | N | *ARRDC3* |
| rs12125664 | 1 | 20082612 | 0.046 | 0.134 | 8.58E-04 | T | A | N | *OTUD3* |
| rs6895675 | 5 | 4127287 | 0.295 | -0.062 | 8.58E-04 | C | G | N | *IRX1* |
| rs2074435 | 19 | 41286499 | 0.405 | 0.056 | 8.59E-04 | A | T | N | *WDR62* |
| rs6960713 | 7 | 45403759 | 0.226 | -0.068 | 8.59E-04 | T | G | N | *ADCY1* |
| rs4862555 | 4 | 186791517 | 0.364 | 0.059 | 8.60E-04 | A | G | N | *SORBS2* |
| rs17669257 | 3 | 60541201 | 0.348 | -0.062 | 8.60E-04 | C | T | N | *FHIT* |
| rs2146244 | 14 | 80856031 | 0.186 | 0.073 | 8.60E-04 | G | A | Y | *STON2* |
| rs2336668 | 3 | 52894136 | 0.366 | 0.058 | 8.60E-04 | G | T | N | *TMEM110* |
| rs17381591 | 10 | 17284054 | 0.330 | -0.060 | 8.60E-04 | C | T | Y | *TRDMT1* |
| rs4687682 | 3 | 52891979 | 0.366 | 0.058 | 8.60E-04 | T | C | N | *TMEM110* |
| rs9860296 | 3 | 52888821 | 0.366 | 0.058 | 8.61E-04 | C | T | N | *TMEM110* |
| rs6795646 | 3 | 52897661 | 0.366 | 0.058 | 8.61E-04 | C | T | Y | *TMEM110* |
| rs955955 | 1 | 149278167 | 0.076 | 0.107 | 8.61E-04 | A | G | N | *BNIPL* |
| rs3821873 | 3 | 52888046 | 0.366 | 0.058 | 8.61E-04 | C | T | N | *TMEM110* |
| rs724176 | 20 | 37142841 | 0.123 | 0.085 | 8.62E-04 | G | A | N | *DHX35* |
| rs3758418 | 10 | 17285644 | 0.330 | -0.060 | 8.62E-04 | C | G | N | *TRDMT1* |
| rs12492391 | 3 | 52887336 | 0.366 | 0.058 | 8.63E-04 | A | C | N | *TMEM110* |
| rs9850563 | 3 | 52887254 | 0.366 | 0.058 | 8.63E-04 | G | A | N | *TMEM110* |
| rs4687680 | 3 | 52882412 | 0.367 | 0.058 | 8.63E-04 | A | G | N | *TMEM110* |
| rs4741718 | 9 | 2496790 | 0.283 | 0.065 | 8.64E-04 | T | A | N | *VLDLR* |
| rs7597279 | 2 | 229456590 | 0.228 | 0.068 | 8.64E-04 | T | C | N | *PID1* |
| rs11702752 | 21 | 41446611 | 0.214 | -0.069 | 8.64E-04 | C | T | N | *BACE2* |
| rs1395389 | 11 | 131073312 | 0.184 | 0.073 | 8.64E-04 | A | G | Y | *HNT* |
| rs10877323 | 12 | 58315221 | 0.184 | 0.073 | 8.64E-04 | C | T | Y | *SLC16A7* |
| rs12116837 | 1 | 20102615 | 0.046 | 0.134 | 8.64E-04 | A | C | N | *OTUD3* |
| rs2336664 | 3 | 52880253 | 0.367 | 0.059 | 8.65E-04 | G | A | N | *TMEM110* |
| rs9836499 | 3 | 52879564 | 0.367 | 0.059 | 8.66E-04 | G | T | N | *TMEM110* |
| rs4741719 | 9 | 2496794 | 0.283 | 0.065 | 8.66E-04 | C | T | N | *VLDLR* |
| rs12506982 | 4 | 27139272 | 0.165 | 0.076 | 8.67E-04 | T | C | N | *STIM2* |
| rs9880978 | 3 | 52898291 | 0.366 | 0.058 | 8.67E-04 | G | C | N | *TMEM110* |
| rs17122765 | 12 | 58320575 | 0.184 | 0.073 | 8.67E-04 | A | G | N | *SLC16A7* |
| rs10075861 | 5 | 63606612 | 0.170 | -0.075 | 8.68E-04 | G | A | N | *RNF180* |
| rs323846 | 12 | 124445806 | 0.405 | -0.057 | 8.68E-04 | T | C | Y | *TMEM132B* |
| rs957403 | 11 | 131073212 | 0.184 | 0.073 | 8.68E-04 | T | C | N | *HNT* |
| rs16892609 | 5 | 63636980 | 0.170 | -0.075 | 8.68E-04 | G | C | Y | *RNF180* |
| rs8126273 | 20 | 15096637 | 0.081 | 0.107 | 8.68E-04 | A | G | N | *MACROD2* |
| rs4931299 | 12 | 30294655 | 0.428 | 0.057 | 8.69E-04 | T | A | N | *IPO8* |
| rs643975 | 6 | 142885944 | 0.192 | 0.071 | 8.69E-04 | G | C | Y | *GPR126* |
| rs10843682 | 12 | 30301523 | 0.396 | 0.058 | 8.69E-04 | A | G | N | *IPO8* |
| rs2581828 | 3 | 53108189 | 0.420 | -0.058 | 8.69E-04 | C | G | N | *RFT1* |
| rs2933343 | 3 | 130141182 | 0.222 | -0.068 | 8.70E-04 | A | G | N | *ACAD9* |
| rs9810807 | 3 | 52899267 | 0.366 | 0.058 | 8.70E-04 | C | T | N | *TMEM110* |
| rs1731512 | 12 | 30299601 | 0.396 | 0.058 | 8.71E-04 | G | A | N | *IPO8* |
| rs4692036 | 4 | 27137299 | 0.165 | 0.076 | 8.72E-04 | C | T | Y | *STIM2* |
| rs2474460 | 1 | 1833906 | 0.518 | 0.057 | 8.72E-04 | C | T | N | *CALML6* |
| rs4692235 | 4 | 27138125 | 0.165 | 0.076 | 8.72E-04 | A | T | Y | *STIM2* |
| rs4780075 | 15 | 31154750 | 0.355 | -0.059 | 8.72E-04 | C | T | N | *FMN1* |
| rs2581815 | 3 | 52957199 | 0.233 | 0.066 | 8.72E-04 | G | C | Y | *SFMBT1* |
| rs11623690 | 14 | 97952411 | 0.195 | 0.074 | 8.73E-04 | G | T | N | *C14orf177* |
| rs11058153 | 12 | 124414492 | 0.406 | -0.057 | 8.73E-04 | G | A | N | *TMEM132B* |
| rs4012257 | 3 | 188213090 | 0.223 | -0.068 | 8.73E-04 | A | C | N | *ST6GAL1* |
| rs7123678 | 11 | 26022477 | 0.150 | 0.082 | 8.74E-04 | A | G | N | *TMEM16C* |
| rs956013 | 11 | 131072926 | 0.184 | 0.073 | 8.74E-04 | G | C | N | *HNT* |
| rs2581780 | 3 | 53034000 | 0.233 | 0.067 | 8.75E-04 | C | T | N | *SFMBT1* |
| rs13131713 | 4 | 11242526 | 0.364 | 0.060 | 8.75E-04 | C | G | N | *HS3ST1* |
| rs10843685 | 12 | 30303122 | 0.396 | 0.058 | 8.77E-04 | A | G | N | *IPO8* |
| rs12232252 | 15 | 37379830 | 0.034 | 0.158 | 8.77E-04 | T | G | N | *FLJ39531* |
| rs4692228 | 4 | 27137280 | 0.165 | 0.075 | 8.78E-04 | G | A | N | *STIM2* |
| rs6137358 | 20 | 21413380 | 0.330 | -0.060 | 8.78E-04 | T | A | N | *NKX2-2* |
| rs11050755 | 12 | 30303251 | 0.396 | 0.058 | 8.80E-04 | A | G | N | *IPO8* |
| rs1148448 | 12 | 100187516 | 0.381 | -0.059 | 8.81E-04 | C | T | N | *UTP20* |
| rs2025142 | 20 | 15102045 | 0.081 | 0.105 | 8.81E-04 | C | T | N | *MACROD2* |
| rs290179 | 11 | 85077711 | 0.406 | 0.059 | 8.81E-04 | A | G | N | *CCDC89* |
| rs6110538 | 20 | 15101532 | 0.081 | 0.105 | 8.82E-04 | G | A | N | *MACROD2* |
| rs3872721 | 3 | 188208185 | 0.226 | -0.068 | 8.82E-04 | G | A | N | *ST6GAL1* |
| rs16967647 | 13 | 105350547 | 0.147 | -0.080 | 8.82E-04 | A | G | Y | *DAOA* |
| rs7728035 | 5 | 997298 | 0.104 | 0.091 | 8.82E-04 | G | A | Y | *TRIP13* |
| rs11726196 | 4 | 123025678 | 0.350 | 0.059 | 8.82E-04 | A | C | Y | *TRPC3* |
| rs2564947 | 3 | 53021256 | 0.233 | 0.067 | 8.82E-04 | C | T | N | *SFMBT1* |
| rs10804905 | 3 | 187569572 | 0.230 | 0.070 | 8.82E-04 | T | C | N | *DGKG* |
| rs1683778 | 3 | 130127285 | 0.223 | -0.068 | 8.83E-04 | G | A | N | *ACAD9* |
| rs11857697 | 15 | 50657272 | 0.101 | 0.097 | 8.83E-04 | A | G | N | *KIAA1370* |
| rs10846881 | 12 | 124412850 | 0.406 | -0.058 | 8.84E-04 | T | G | N | *TMEM132B* |
| rs1353102 | 5 | 4122616 | 0.295 | -0.062 | 8.84E-04 | C | G | N | *IRX1* |
| rs12232251 | 15 | 37379846 | 0.034 | 0.158 | 8.85E-04 | T | C | N | *FLJ39531* |
| rs245243 | 5 | 109258634 | 0.374 | 0.058 | 8.85E-04 | A | G | Y | *MAN2A1* |
| rs3006644 | 10 | 32458802 | 0.351 | 0.060 | 8.85E-04 | G | C | N | *KIF5B* |
| rs12868476 | 13 | 104417447 | 0.120 | -0.086 | 8.85E-04 | T | G | Y | *DAOA* |
| rs2327916 | 20 | 15104733 | 0.081 | 0.104 | 8.86E-04 | G | A | N | *MACROD2* |
| rs17158481 | 7 | 29927823 | 0.039 | -0.144 | 8.86E-04 | C | G | Y | *SCRN1* |
| rs2564942 | 3 | 53114546 | 0.406 | -0.059 | 8.86E-04 | C | T | N | *RFT1* |
| rs17158473 | 7 | 29926204 | 0.039 | -0.144 | 8.86E-04 | T | C | Y | *SCRN1* |
| rs4492142 | 5 | 90962109 | 0.503 | 0.056 | 8.87E-04 | T | G | N | *ARRDC3* |
| rs1441838 | 1 | 29989826 | 0.446 | 0.058 | 8.88E-04 | T | C | N | *PTPRU* |
| rs9338477 | 10 | 32459221 | 0.351 | 0.060 | 8.89E-04 | G | T | N | *KIF5B* |
| rs7268079 | 20 | 15105914 | 0.081 | 0.104 | 8.89E-04 | G | A | Y | *MACROD2* |
| rs7648514 | 3 | 52958286 | 0.232 | 0.066 | 8.89E-04 | G | C | N | *SFMBT1* |
| rs2058203 | 17 | 58876736 | 0.454 | 0.058 | 8.89E-04 | G | C | N | *CYB561* |
| rs7813508 | 8 | 87177043 | 0.358 | 0.060 | 8.89E-04 | T | C | N | *ATP6V0D2* |
| rs11050749 | 12 | 30298369 | 0.396 | 0.058 | 8.90E-04 | C | G | N | *IPO8* |
| rs6079691 | 20 | 15102338 | 0.081 | 0.105 | 8.90E-04 | C | T | N | *MACROD2* |
| rs8069445 | 17 | 376995 | 0.017 | 0.227 | 8.91E-04 | T | G | N | *VPS53* |
| rs2215231 | 4 | 11243225 | 0.362 | 0.059 | 8.92E-04 | C | G | N | *HS3ST1* |
| rs876755 | 3 | 130110623 | 0.223 | -0.068 | 8.92E-04 | T | C | N | *ACAD9* |
| rs10203061 | 2 | 68135263 | 0.179 | 0.074 | 8.92E-04 | G | A | N | *C1D* |
| rs2271064 | 15 | 37379987 | 0.034 | 0.158 | 8.92E-04 | A | G | N | *FLJ39531* |
| rs2047074 | 5 | 4133229 | 0.286 | -0.064 | 8.92E-04 | C | T | N | *IRX1* |
| rs2124370 | 17 | 18050053 | 0.109 | 0.091 | 8.93E-04 | A | C | N | *ALKBH5* |
| rs2864124 | 1 | 55247267 | 0.411 | -0.059 | 8.94E-04 | G | A | N | *BSND* |
| rs12472073 | 2 | 229450837 | 0.231 | 0.067 | 8.94E-04 | C | T | N | *PID1* |
| rs2884038 | 10 | 17292164 | 0.354 | -0.061 | 8.94E-04 | C | T | N | *TRDMT1* |
| rs3006649 | 10 | 32460080 | 0.351 | 0.060 | 8.95E-04 | A | G | N | *KIF5B* |
| rs7501868 | 17 | 7862776 | 0.484 | -0.056 | 8.95E-04 | T | C | Y | *GUCY2D* |
| rs4623914 | 11 | 131943904 | 0.312 | -0.061 | 8.96E-04 | C | T | N | *OPCML* |
| rs4331881 | 5 | 156877053 | 0.350 | -0.059 | 8.96E-04 | T | G | Y | *ADAM19* |
| rs789237 | 3 | 130119651 | 0.223 | -0.068 | 8.96E-04 | C | A | Y | *ACAD9* |
| rs6445550 | 3 | 52958474 | 0.232 | 0.066 | 8.98E-04 | A | G | N | *SFMBT1* |
| rs2835293 | 21 | 36464288 | 0.471 | -0.056 | 8.98E-04 | A | G | Y | *DOPEY2* |
| rs4274344 | 14 | 56042391 | 0.450 | 0.057 | 8.99E-04 | C | T | Y | *C14orf101* |
| rs8045890 | 16 | 79847927 | 0.220 | -0.071 | 8.99E-04 | C | G | N | *BCMO1* |
| rs7940010 | 11 | 26026489 | 0.139 | 0.081 | 9.00E-04 | C | G | N | *TMEM16C* |
| rs1477782 | 3 | 179846808 | 0.162 | -0.079 | 9.00E-04 | G | A | N | *KCNMB2* |
| rs10255669 | 7 | 69424678 | 0.052 | 0.130 | 9.01E-04 | C | T | N | *AUTS2* |
| rs13142540 | 4 | 48478344 | 0.488 | -0.057 | 9.01E-04 | C | T | N | *FRYL* |
| rs6799972 | 3 | 38844365 | 0.272 | -0.063 | 9.01E-04 | G | C | Y | *SCN11A* |
| rs11257600 | 10 | 12295663 | 0.243 | -0.067 | 9.02E-04 | T | G | N | *NUDT5* |
| rs7531583 | 1 | 1696020 | 0.246 | 0.067 | 9.02E-04 | A | G | N | *NADK* |
| rs912180 | 20 | 37144213 | 0.124 | 0.085 | 9.02E-04 | T | G | N | *DHX35* |
| rs943645 | 1 | 55248476 | 0.411 | -0.059 | 9.02E-04 | A | G | N | *BSND* |
| rs2495511 | 1 | 55248766 | 0.411 | -0.059 | 9.02E-04 | G | A | N | *BSND* |
| rs7779192 | 7 | 53457655 | 0.289 | 0.065 | 9.03E-04 | C | T | N | *DKFZp564N24* |
| rs7775215 | 6 | 82856591 | 0.377 | 0.058 | 9.03E-04 | C | T | Y | *IBTK* |
| rs17051768 | 13 | 34436841 | 0.059 | -0.119 | 9.03E-04 | C | G | N | *NBEA* |
| rs12505179 | 4 | 27118509 | 0.161 | 0.076 | 9.04E-04 | C | T | N | *STIM2* |
| rs3800204 | 6 | 132823165 | 0.214 | 0.069 | 9.04E-04 | T | G | N | *STX7* |
| rs9483454 | 6 | 132825558 | 0.187 | 0.073 | 9.04E-04 | A | C | Y | *STX7* |
| rs9865094 | 3 | 52960359 | 0.232 | 0.066 | 9.05E-04 | G | C | N | *SFMBT1* |
| rs12505247 | 4 | 27118719 | 0.161 | 0.076 | 9.05E-04 | C | T | Y | *STIM2* |
| rs1959060 | 14 | 56042218 | 0.449 | 0.057 | 9.06E-04 | A | G | Y | *C14orf101* |
| rs7805779 | 7 | 45416938 | 0.228 | -0.067 | 9.07E-04 | C | A | Y | *ADCY1* |
| rs1465787 | 20 | 57234620 | 0.099 | 0.094 | 9.07E-04 | G | C | Y | *C20orf174* |
| rs4810162 | 20 | 57233719 | 0.099 | 0.094 | 9.08E-04 | C | T | N | *C20orf174* |
| rs4692223 | 4 | 27118204 | 0.161 | 0.076 | 9.09E-04 | C | A | N | *STIM2* |
| rs11155242 | 6 | 142733242 | 0.211 | 0.069 | 9.09E-04 | C | A | Y | *GPR126* |
| rs2143277 | 20 | 47909411 | 0.088 | 0.100 | 9.10E-04 | T | C | N | *SLC9A8* |
| rs17638800 | 16 | 27089322 | 0.278 | 0.061 | 9.10E-04 | A | G | Y | *JMJD5* |
| rs17668955 | 10 | 26866912 | 0.140 | 0.084 | 9.11E-04 | C | G | N | *APBB1IP* |
| rs1465786 | 20 | 57234690 | 0.099 | 0.094 | 9.11E-04 | C | A | N | *C20orf174* |
| rs7566951 | 2 | 229448761 | 0.232 | 0.067 | 9.12E-04 | C | T | N | *PID1* |
| rs10791236 | 11 | 131942046 | 0.325 | -0.061 | 9.12E-04 | G | A | N | *OPCML* |
| rs6938215 | 6 | 49162724 | 0.369 | 0.058 | 9.12E-04 | G | A | Y | *MUT* |
| rs1680790 | 3 | 130108543 | 0.223 | -0.068 | 9.13E-04 | T | G | N | *ACAD9* |
| rs7321833 | 13 | 34471362 | 0.059 | -0.119 | 9.13E-04 | C | A | N | *NBEA* |
| rs17051492 | 13 | 34469323 | 0.059 | -0.119 | 9.13E-04 | A | T | Y | *NBEA* |
| rs6486919 | 12 | 18859873 | 0.262 | 0.064 | 9.13E-04 | C | G | N | *CAPZA3* |
| rs6537285 | 4 | 145529597 | 0.298 | -0.062 | 9.13E-04 | A | G | N | *GYPA* |
| rs3793249 | 7 | 29937122 | 0.017 | -0.216 | 9.13E-04 | A | G | Y | *SCRN1* |
| rs17158518 | 7 | 29938482 | 0.017 | -0.216 | 9.13E-04 | G | T | Y | *SCRN1* |
| rs17774785 | 13 | 34477756 | 0.059 | -0.119 | 9.13E-04 | C | T | Y | *NBEA* |
| rs17158520 | 7 | 29940174 | 0.017 | -0.216 | 9.13E-04 | A | G | N | *SCRN1* |
| rs1935941 | 6 | 121382441 | 0.210 | 0.069 | 9.14E-04 | T | C | N | *C6orf170* |
| rs10490037 | 2 | 229449576 | 0.232 | 0.067 | 9.14E-04 | C | T | Y | *PID1* |
| rs9372634 | 6 | 121382754 | 0.210 | 0.069 | 9.14E-04 | T | C | Y | *C6orf170* |
| rs2358167 | 6 | 121378412 | 0.210 | 0.069 | 9.15E-04 | A | G | N | *C6orf170* |
| rs1884706 | 20 | 15396092 | 0.436 | 0.058 | 9.15E-04 | T | G | N | *MACROD2* |
| rs1418921 | 6 | 121377335 | 0.210 | 0.069 | 9.15E-04 | T | C | Y | *C6orf170* |
| rs8024232 | 15 | 31153574 | 0.354 | -0.058 | 9.16E-04 | G | T | N | *FMN1* |
| rs569009 | 3 | 174637969 | 0.122 | -0.087 | 9.16E-04 | T | C | N | *NLGN1* |
| rs617968 | 3 | 174639275 | 0.122 | -0.086 | 9.17E-04 | A | G | N | *NLGN1* |
| rs1502254 | 4 | 27128256 | 0.161 | 0.076 | 9.17E-04 | T | A | N | *STIM2* |
| rs9311485 | 3 | 52962685 | 0.232 | 0.066 | 9.17E-04 | T | G | N | *SFMBT1* |
| rs6768844 | 3 | 52979660 | 0.233 | 0.066 | 9.17E-04 | T | C | N | *SFMBT1* |
| rs580025 | 11 | 115484563 | 0.225 | 0.068 | 9.17E-04 | G | T | N | *CADM1* |
| rs566054 | 3 | 174638319 | 0.122 | -0.087 | 9.18E-04 | T | C | N | *NLGN1* |
| rs1502255 | 4 | 27128287 | 0.161 | 0.076 | 9.18E-04 | G | C | N | *STIM2* |
| rs13049454 | 21 | 41448390 | 0.213 | -0.069 | 9.18E-04 | A | G | N | *BACE2* |
| rs7645845 | 3 | 20741288 | 0.082 | 0.103 | 9.19E-04 | C | G | N | *SGOL1* |
| rs1349964 | 15 | 31140281 | 0.355 | -0.058 | 9.20E-04 | C | T | Y | *FMN1* |
| rs2358133 | 6 | 121383296 | 0.210 | 0.069 | 9.20E-04 | T | C | N | *C6orf170* |
| rs6792578 | 3 | 52979644 | 0.233 | 0.066 | 9.20E-04 | A | G | N | *SFMBT1* |
| rs17188132 | 13 | 34402203 | 0.060 | -0.119 | 9.21E-04 | A | G | N | *NBEA* |
| rs2336669 | 3 | 52969178 | 0.232 | 0.066 | 9.22E-04 | G | A | Y | *SFMBT1* |
| rs4519686 | 3 | 52972911 | 0.232 | 0.066 | 9.22E-04 | T | C | Y | *SFMBT1* |
| rs17158488 | 7 | 29929772 | 0.039 | -0.144 | 9.22E-04 | C | T | N | *SCRN1* |
| rs17141545 | 7 | 69493109 | 0.051 | 0.128 | 9.23E-04 | G | A | Y | *AUTS2* |
| rs4361636 | 6 | 132826161 | 0.214 | 0.069 | 9.23E-04 | C | T | N | *STX7* |
| rs262120 | 6 | 142884053 | 0.196 | 0.071 | 9.24E-04 | A | C | N | *GPR126* |
| rs11070516 | 15 | 44489285 | 0.325 | 0.061 | 9.24E-04 | C | T | N | *SQRDL* |
| rs1390240 | 3 | 65747737 | 0.504 | 0.057 | 9.24E-04 | C | G | N | *MAGI1* |
| rs11662440 | 18 | 68578290 | 0.380 | -0.058 | 9.24E-04 | T | C | N | *NETO1* |
| rs8084619 | 18 | 29462658 | 0.491 | 0.056 | 9.25E-04 | A | T | N | *ASXL3* |
| rs12642044 | 4 | 27153484 | 0.167 | 0.076 | 9.25E-04 | T | C | N | *STIM2* |
| rs17773682 | 13 | 34392027 | 0.060 | -0.119 | 9.26E-04 | G | A | N | *NBEA* |
| rs17158499 | 7 | 29932377 | 0.039 | -0.144 | 9.27E-04 | G | C | N | *SCRN1* |
| rs17051506 | 13 | 34394751 | 0.060 | -0.119 | 9.28E-04 | A | G | N | *NBEA* |
| rs789532 | 11 | 131067588 | 0.184 | 0.074 | 9.28E-04 | T | C | N | *HNT* |
| rs9305726 | 21 | 41447142 | 0.213 | -0.069 | 9.30E-04 | A | G | N | *BACE2* |
| rs9636612 | 21 | 15318862 | 0.352 | -0.061 | 9.31E-04 | G | A | N | *NRIP1* |
| rs17051511 | 13 | 34398167 | 0.060 | -0.119 | 9.32E-04 | C | T | N | *NBEA* |
| rs355130 | 3 | 8587086 | 0.217 | 0.068 | 9.32E-04 | G | T | N | *LMCD1* |
| rs685464 | 15 | 51170130 | 0.019 | -0.208 | 9.32E-04 | C | G | N | *ONECUT1* |
| rs6027429 | 20 | 58236992 | 0.224 | -0.070 | 9.32E-04 | C | G | N | *C20orf197* |
| rs1480964 | 4 | 34825779 | 0.421 | -0.058 | 9.33E-04 | A | T | N | *CENTD1* |
| rs2816 | 17 | 7864289 | 0.484 | -0.056 | 9.34E-04 | T | C | Y | *GUCY2D* |
| rs10041997 | 5 | 120226210 | 0.424 | -0.057 | 9.34E-04 | A | G | N | *PRR16* |
| rs7667097 | 4 | 14078492 | 0.463 | 0.057 | 9.35E-04 | A | G | N | *CPEB2* |
| rs2630252 | 3 | 130107462 | 0.222 | -0.067 | 9.35E-04 | C | T | N | *ACAD9* |
| rs311499 | 20 | 61688033 | 0.087 | 0.099 | 9.35E-04 | T | C | N | *GMEB2* |
| rs17158502 | 7 | 29932819 | 0.039 | -0.144 | 9.35E-04 | T | C | N | *SCRN1* |
| rs2174717 | 10 | 55499743 | 0.068 | -0.117 | 9.36E-04 | C | G | N | *PCDH15* |
| rs2349795 | 11 | 26026879 | 0.096 | 0.094 | 9.37E-04 | C | T | N | *TMEM16C* |
| rs1564720 | 1 | 34343171 | 0.383 | -0.060 | 9.37E-04 | A | G | N | *CSMD2* |
| rs1953261 | 14 | 56043337 | 0.450 | 0.057 | 9.38E-04 | C | T | N | *C14orf101* |
| rs311498 | 20 | 61688058 | 0.087 | 0.099 | 9.38E-04 | A | G | N | *GMEB2* |
| rs9350405 | 6 | 22103055 | 0.416 | 0.059 | 9.39E-04 | G | A | N | *PRL* |
| rs613485 | 7 | 47554402 | 0.303 | -0.061 | 9.39E-04 | C | T | Y | *TNS3* |
| rs10220329 | 14 | 77830994 | 0.437 | -0.057 | 9.39E-04 | C | T | N | *NRXN3* |
| rs1683776 | 3 | 130105450 | 0.222 | -0.067 | 9.39E-04 | T | G | N | *ACAD9* |
| rs11761585 | 7 | 47553874 | 0.303 | -0.061 | 9.39E-04 | G | A | N | *TNS3* |
| rs10132942 | 14 | 90321721 | 0.120 | 0.086 | 9.40E-04 | T | G | Y | *TTC7B* |
| rs7566116 | 2 | 164669628 | 0.160 | 0.078 | 9.41E-04 | A | G | N | *FIGN* |
| rs10974671 | 9 | 4620713 | 0.032 | 0.159 | 9.41E-04 | G | A | Y | *PPAPDC2* |
| rs2830172 | 21 | 26613770 | 0.450 | -0.056 | 9.41E-04 | C | A | N | *CYYR1* |
| rs160396 | 20 | 61688972 | 0.087 | 0.099 | 9.41E-04 | A | G | N | *GMEB2* |
| rs6764111 | 3 | 53182441 | 0.208 | -0.070 | 9.42E-04 | A | G | N | *PRKCD* |
| rs10464238 | 7 | 47557299 | 0.303 | -0.061 | 9.42E-04 | C | G | N | *TNS3* |
| rs1949804 | 7 | 69406762 | 0.052 | 0.129 | 9.42E-04 | G | T | N | *AUTS2* |
| rs1151621 | 20 | 61690281 | 0.087 | 0.099 | 9.42E-04 | A | G | Y | *GMEB2* |
| rs2133823 | 3 | 20740115 | 0.082 | 0.103 | 9.42E-04 | G | A | N | *SGOL1* |
| rs2366312 | 3 | 20741054 | 0.082 | 0.103 | 9.42E-04 | A | G | N | *SGOL1* |
| rs8003935 | 14 | 77830268 | 0.437 | -0.057 | 9.42E-04 | T | C | N | *NRXN3* |
| rs2401913 | 14 | 90304874 | 0.121 | 0.086 | 9.43E-04 | G | A | N | *TTC7B* |
| rs662698 | 7 | 47556841 | 0.303 | -0.061 | 9.44E-04 | G | A | N | *TNS3* |
| rs17758206 | 11 | 86131971 | 0.120 | -0.086 | 9.45E-04 | T | C | Y | *PRSS23* |
| rs10974672 | 9 | 4620762 | 0.032 | 0.159 | 9.45E-04 | T | C | N | *PPAPDC2* |
| rs6580532 | 5 | 147541109 | 0.424 | 0.058 | 9.48E-04 | C | T | N | *SPINK5L2* |
| rs2073328 | 14 | 90321823 | 0.120 | 0.086 | 9.48E-04 | C | T | N | *TTC7B* |
| rs311496 | 20 | 61692054 | 0.087 | 0.099 | 9.48E-04 | T | C | N | *GMEB2* |
| rs2401915 | 14 | 90305057 | 0.121 | 0.086 | 9.49E-04 | T | C | N | *TTC7B* |
| rs2040280 | 21 | 26611712 | 0.450 | -0.056 | 9.50E-04 | G | T | N | *APP* |
| rs1511530 | 3 | 73852548 | 0.413 | -0.057 | 9.50E-04 | G | A | Y | *PDZRN3* |
| rs6884944 | 5 | 147541429 | 0.424 | 0.058 | 9.52E-04 | T | C | N | *SPINK5L2* |
| rs6873352 | 5 | 120256295 | 0.421 | -0.057 | 9.52E-04 | C | T | N | *PRR16* |
| rs10154234 | 21 | 41446877 | 0.213 | -0.069 | 9.52E-04 | A | G | N | *BACE2* |
| rs6015696 | 20 | 58238100 | 0.224 | -0.070 | 9.53E-04 | T | C | N | *C20orf197* |
| rs10767502 | 11 | 26010866 | 0.140 | 0.080 | 9.53E-04 | A | C | N | *TMEM16C* |
| rs10059441 | 5 | 147543409 | 0.425 | 0.058 | 9.55E-04 | A | G | N | *SPINK5L2* |
| rs10238006 | 7 | 32343478 | 0.253 | -0.066 | 9.56E-04 | A | C | N | *LSM5* |
| rs218804 | 4 | 14069481 | 0.450 | 0.056 | 9.56E-04 | A | G | N | *CPEB2* |
| rs311494 | 20 | 61693815 | 0.087 | 0.099 | 9.56E-04 | A | G | N | *GMEB2* |
| rs6888945 | 5 | 120264436 | 0.421 | -0.057 | 9.56E-04 | C | T | N | *PRR16* |
| rs6896988 | 5 | 147543670 | 0.425 | 0.058 | 9.57E-04 | T | C | N | *SPINK5L2* |
| rs9908345 | 17 | 379221 | 0.017 | 0.226 | 9.57E-04 | C | T | N | *VPS53* |
| rs9593057 | 13 | 34766207 | 0.090 | -0.098 | 9.58E-04 | T | C | N | *MAB21L1* |
| rs4884986 | 13 | 71551546 | 0.180 | 0.073 | 9.58E-04 | A | G | N | *DACH1* |
| rs11610226 | 12 | 115470017 | 0.067 | -0.113 | 9.59E-04 | G | T | N | *KRTHB5* |
| rs395791 | 20 | 61700551 | 0.087 | 0.100 | 9.60E-04 | C | G | N | *GMEB2* |
| rs613862 | 3 | 174638361 | 0.123 | -0.086 | 9.61E-04 | T | C | N | *NLGN1* |
| rs16981092 | 20 | 19693011 | 0.363 | -0.060 | 9.61E-04 | G | A | N | *SLC24A3* |
| rs10041939 | 5 | 147545190 | 0.425 | 0.058 | 9.61E-04 | G | T | N | *SPINK5L2* |
| rs7980795 | 12 | 112886950 | 0.412 | 0.056 | 9.61E-04 | A | G | Y | *RBM19* |
| rs2830175 | 21 | 26620430 | 0.450 | -0.056 | 9.61E-04 | G | A | N | *CYYR1* |
| rs8013195 | 14 | 90310700 | 0.121 | 0.086 | 9.61E-04 | G | A | N | *TTC7B* |
| rs17045337 | 2 | 53958119 | 0.124 | 0.086 | 9.62E-04 | C | T | N | *PSME4* |
| rs4499819 | 5 | 147538739 | 0.443 | 0.057 | 9.63E-04 | A | G | Y | *SPINK5L2* |
| rs2413815 | 15 | 44502435 | 0.303 | 0.061 | 9.63E-04 | G | A | N | *SQRDL* |
| rs11263771 | 17 | 32040112 | 0.390 | 0.057 | 9.63E-04 | C | T | Y | *MRM1* |
| rs218800 | 4 | 14067672 | 0.450 | 0.056 | 9.63E-04 | G | T | N | *CPEB2* |
| rs9325087 | 5 | 147546051 | 0.425 | 0.058 | 9.63E-04 | C | T | N | *SPINK5L2* |
| rs6868569 | 5 | 147546007 | 0.425 | 0.058 | 9.63E-04 | T | G | N | *SPINK5L2* |
| rs6878559 | 5 | 120236091 | 0.435 | -0.056 | 9.64E-04 | G | A | Y | *PRR16* |
| rs6506672 | 18 | 9446055 | 0.199 | 0.074 | 9.64E-04 | A | G | N | *RALBP1* |
| rs6100803 | 20 | 58240441 | 0.224 | -0.070 | 9.65E-04 | C | A | N | *C20orf197* |
| rs10974673 | 9 | 4620926 | 0.032 | 0.158 | 9.65E-04 | C | T | N | *PPAPDC2* |
| rs2721047 | 13 | 40025682 | 0.096 | 0.095 | 9.65E-04 | T | C | Y | *FOXO1* |
| rs6517654 | 21 | 41456198 | 0.212 | -0.069 | 9.65E-04 | C | G | Y | *BACE2* |
| rs7153346 | 14 | 90314449 | 0.121 | 0.086 | 9.66E-04 | T | C | N | *TTC7B* |
| rs2073338 | 10 | 105632793 | 0.338 | -0.059 | 9.66E-04 | C | A | N | *OBFC1* |
| rs355132 | 3 | 8587576 | 0.217 | 0.068 | 9.67E-04 | G | A | N | *LMCD1* |
| rs981331 | 8 | 55966980 | 0.268 | 0.063 | 9.68E-04 | T | A | N | *XKR4* |
| rs17436310 | 2 | 164710071 | 0.159 | 0.077 | 9.68E-04 | C | T | N | *GRB14* |
| rs13203060 | 6 | 82846398 | 0.374 | 0.058 | 9.68E-04 | A | C | N | *IBTK* |
| rs870411 | 21 | 26621680 | 0.450 | -0.056 | 9.68E-04 | G | A | N | *CYYR1* |
| rs4939885 | 18 | 45426301 | 0.033 | 0.164 | 9.68E-04 | T | C | N | *LIPG* |
| rs8011601 | 14 | 90316777 | 0.121 | 0.086 | 9.69E-04 | A | G | N | *TTC7B* |
| rs2040668 | 7 | 27925298 | 0.217 | -0.069 | 9.69E-04 | G | T | N | *JAZF1* |
| rs4899777 | 14 | 80268984 | 0.086 | 0.100 | 9.70E-04 | A | G | Y | *C14orf145* |
| rs242078 | 22 | 31558692 | 0.502 | -0.057 | 9.70E-04 | A | G | N | *TIMP3* |
| rs2164166 | 6 | 80667558 | 0.401 | 0.057 | 9.70E-04 | G | A | N | *ELOVL4* |
| rs10517365 | 4 | 56481588 | 0.170 | -0.075 | 9.70E-04 | T | C | Y | *EXOC1* |
| rs1592153 | 9 | 95785657 | 0.504 | -0.056 | 9.71E-04 | C | A | Y | *BARX1* |
| rs2153058 | 9 | 95785947 | 0.504 | -0.056 | 9.71E-04 | A | G | Y | *BARX1* |
| rs987511 | 9 | 95797689 | 0.508 | -0.056 | 9.72E-04 | A | G | Y | *PTPDC1* |
| rs2058979 | 10 | 107411488 | 0.152 | 0.079 | 9.72E-04 | G | A | N | *SORCS3* |
| rs12645910 | 4 | 145526871 | 0.299 | -0.061 | 9.72E-04 | T | G | N | *GYPA* |
| rs8003457 | 14 | 90303587 | 0.121 | 0.086 | 9.73E-04 | A | G | N | *TTC7B* |
| rs17034884 | 2 | 68132547 | 0.179 | 0.074 | 9.73E-04 | C | T | N | *C1D* |
| rs179256 | 14 | 80507823 | 0.106 | 0.091 | 9.74E-04 | T | G | N | *TSHR* |
| rs1590969 | 9 | 95796257 | 0.508 | -0.056 | 9.74E-04 | T | G | N | *PTPDC1* |
| rs10815040 | 9 | 4621605 | 0.032 | 0.158 | 9.74E-04 | G | C | N | *PPAPDC2* |
| rs13202517 | 6 | 82845765 | 0.374 | 0.058 | 9.75E-04 | A | G | N | *IBTK* |
| rs10090710 | 8 | 55970246 | 0.268 | 0.063 | 9.75E-04 | G | C | Y | *XKR4* |
| rs1801041 | 10 | 69844713 | 0.280 | -0.062 | 9.75E-04 | A | T | Y | *DNA2L* |
| rs10761309 | 9 | 95793543 | 0.508 | -0.056 | 9.75E-04 | C | T | N | *BARX1* |
| rs690031 | 15 | 51189247 | 0.020 | -0.203 | 9.75E-04 | A | C | N | *ONECUT1* |
| rs17429150 | 11 | 70259589 | 0.083 | -0.105 | 9.76E-04 | C | T | N | *SHANK2* |
| rs2373038 | 14 | 85484033 | 0.358 | -0.060 | 9.76E-04 | A | G | N | *FLRT2* |
| rs1316814 | 9 | 95782539 | 0.504 | -0.056 | 9.76E-04 | G | A | N | *BARX1* |
| rs10041968 | 5 | 120247239 | 0.435 | -0.056 | 9.76E-04 | C | T | N | *PRR16* |
| rs6109666 | 20 | 12971875 | 0.185 | -0.074 | 9.77E-04 | G | T | N | *SPTLC3* |
| rs17775456 | 13 | 34531030 | 0.059 | -0.119 | 9.77E-04 | T | A | N | *NBEA* |
| rs7192417 | 16 | 83591618 | 0.278 | -0.062 | 9.78E-04 | G | T | N | *ZDHHC7* |
| rs11082770 | 18 | 45429668 | 0.033 | 0.165 | 9.79E-04 | C | A | N | *LIPG* |
| rs870542 | 9 | 80368298 | 0.195 | 0.071 | 9.79E-04 | T | C | N | *PSAT1* |
| rs7085460 | 10 | 17288673 | 0.331 | -0.059 | 9.80E-04 | C | G | N | *TRDMT1* |
| rs789529 | 11 | 131071146 | 0.185 | 0.072 | 9.81E-04 | T | C | Y | *HNT* |
| rs906493 | 4 | 123025071 | 0.349 | 0.059 | 9.81E-04 | C | T | N | *TRPC3* |
| rs17011477 | 3 | 73871633 | 0.115 | -0.088 | 9.81E-04 | A | G | N | *PDZRN3* |
| rs10774872 | 12 | 115470278 | 0.067 | -0.113 | 9.81E-04 | C | G | N | *KRTHB5* |
| rs3862082 | 8 | 17514969 | 0.400 | -0.059 | 9.81E-04 | T | C | N | *PDGFRL* |
| rs13272473 | 8 | 23234924 | 0.275 | 0.062 | 9.82E-04 | C | T | N | *LOXL2* |
| rs12512911 | 4 | 34825047 | 0.421 | -0.057 | 9.82E-04 | C | T | N | *CENTD1* |
| rs7950942 | 11 | 131939578 | 0.314 | -0.061 | 9.83E-04 | A | G | N | *OPCML* |
| rs1360429 | 9 | 95802135 | 0.509 | -0.056 | 9.83E-04 | T | C | N | *PTPDC1* |
| rs11875246 | 18 | 65640955 | 0.118 | -0.087 | 9.84E-04 | T | C | N | *DOK6* |
| rs13309098 | 7 | 48782052 | 0.076 | -0.106 | 9.84E-04 | A | G | N | *ABCA13* |
| rs10828550 | 10 | 24129864 | 0.178 | -0.074 | 9.84E-04 | T | G | N | *KIAA1217* |
| rs13088125 | 3 | 80403153 | 0.274 | -0.064 | 9.85E-04 | A | G | N | *ROBO1* |
| rs17583598 | 4 | 149897829 | 0.327 | -0.060 | 9.85E-04 | T | G | N | *NR3C2* |
| rs412147 | 9 | 98025142 | 0.266 | -0.063 | 9.85E-04 | G | A | Y | *HSD17B3* |
| rs7240235 | 18 | 68621559 | 0.423 | 0.056 | 9.85E-04 | C | G | Y | *NETO1* |
| rs1848266 | 18 | 71953761 | 0.092 | 0.099 | 9.85E-04 | T | A | N | *ZNF516* |
| rs4706815 | 6 | 80667311 | 0.401 | 0.057 | 9.86E-04 | A | G | N | *ELOVL4* |
| rs12674192 | 7 | 48780748 | 0.076 | -0.106 | 9.86E-04 | A | G | Y | *ABCA13* |
| rs12522694 | 5 | 147541492 | 0.451 | 0.058 | 9.87E-04 | C | A | N | *SPINK5L2* |
| rs17051983 | 13 | 34754622 | 0.090 | -0.098 | 9.87E-04 | C | G | N | *MAB21L1* |
| rs10834904 | 11 | 26041080 | 0.111 | 0.092 | 9.87E-04 | T | C | N | *TMEM16C* |
| rs8085412 | 18 | 71955486 | 0.092 | 0.099 | 9.87E-04 | C | T | N | *ZNF516* |
| rs4570851 | 16 | 26869080 | 0.313 | -0.061 | 9.87E-04 | C | G | N | *C16orf82* |
| rs9548545 | 13 | 38405823 | 0.056 | 0.126 | 9.89E-04 | T | C | N | *STOML3* |
| rs2055159 | 15 | 36790396 | 0.237 | -0.068 | 9.90E-04 | T | A | N | *FLJ35695* |
| rs6109667 | 20 | 12972130 | 0.185 | -0.074 | 9.90E-04 | C | T | N | *SPTLC3* |
| rs11702604 | 21 | 41446740 | 0.213 | -0.068 | 9.90E-04 | T | C | Y | *BACE2* |
| rs10786773 | 10 | 105634265 | 0.338 | -0.059 | 9.91E-04 | T | A | N | *OBFC1* |
| rs9493327 | 6 | 132827261 | 0.214 | 0.068 | 9.91E-04 | A | T | N | *STX7* |
| rs4891124 | 18 | 71955914 | 0.092 | 0.100 | 9.91E-04 | T | C | N | *ZNF516* |
| rs2291830 | 1 | 212570112 | 0.430 | -0.057 | 9.92E-04 | T | G | N | *SMYD2* |
| rs12684427 | 9 | 95763624 | 0.504 | -0.056 | 9.92E-04 | C | T | N | *BARX1* |
| rs8085208 | 18 | 71956611 | 0.092 | 0.100 | 9.92E-04 | A | G | N | *ZNF516* |
| rs4891125 | 18 | 71956018 | 0.092 | 0.100 | 9.92E-04 | G | A | N | *ZNF516* |
| rs10207196 | 2 | 68123762 | 0.189 | 0.074 | 9.92E-04 | T | C | N | *C1D* |
| rs4891126 | 18 | 71956147 | 0.092 | 0.100 | 9.93E-04 | C | T | N | *ZNF516* |
| rs4282631 | 9 | 95804231 | 0.509 | -0.056 | 9.93E-04 | C | T | N | *PTPDC1* |
| rs4394881 | 12 | 97436159 | 0.447 | 0.057 | 9.94E-04 | A | T | Y | *TMPO* |
| rs1320547 | 9 | 95759545 | 0.504 | -0.056 | 9.94E-04 | T | C | N | *BARX1* |
| rs12232322 | 15 | 37380031 | 0.034 | 0.157 | 9.94E-04 | C | T | N | *FLJ39531* |
| rs4801316 | 19 | 61597831 | 0.265 | 0.065 | 9.94E-04 | A | G | N | *ZNF582* |
| rs12197866 | 6 | 142747756 | 0.210 | 0.069 | 9.94E-04 | T | C | N | *GPR126* |
| rs10008853 | 4 | 170772533 | 0.062 | -0.120 | 9.95E-04 | A | G | N | *NEK1* |
| rs2094927 | 9 | 95805207 | 0.509 | -0.056 | 9.95E-04 | A | C | N | *PTPDC1* |
| rs6074854 | 20 | 15116497 | 0.081 | 0.104 | 9.96E-04 | A | G | N | *MACROD2* |
| rs8085745 | 18 | 71956978 | 0.091 | 0.101 | 9.96E-04 | A | G | N | *ZNF516* |
| rs17007502 | 3 | 20738139 | 0.082 | 0.102 | 9.96E-04 | G | A | Y | *SGOL1* |
| rs1112549 | 12 | 83450589 | 0.424 | 0.056 | 9.97E-04 | T | A | N | *SLC6A15* |
| rs11982953 | 7 | 45426258 | 0.227 | -0.066 | 9.97E-04 | A | G | Y | *ADCY1* |
| rs4458329 | 3 | 80402243 | 0.271 | -0.064 | 9.98E-04 | T | C | N | *ROBO1* |
| rs4479553 | 3 | 80402166 | 0.271 | -0.064 | 9.98E-04 | T | C | N | *ROBO1* |
| rs12313200 | 12 | 12175857 | 0.174 | -0.074 | 9.98E-04 | A | G | Y | *LRP6* |
| rs6811667 | 4 | 145526629 | 0.299 | -0.061 | 9.98E-04 | C | G | N | *GYPA* |
| rs4457535 | 1 | 176763809 | 0.383 | 0.057 | 9.98E-04 | G | A | Y | *C1orf49* |
| rs4418790 | 11 | 15403390 | 0.469 | 0.057 | 9.99E-04 | T | C | N | *INSC* |
| rs7095324 | 10 | 17290906 | 0.331 | -0.059 | 9.99E-04 | T | G | N | *TRDMT1* |
| rs6718664 | 2 | 229468485 | 0.238 | 0.069 | 9.99E-04 | A | C | N | *PID1* |
| rs9807970 | 2 | 68127601 | 0.179 | 0.073 | 9.99E-04 | C | T | N | *C1D* |
| rs4787884 | 16 | 26869578 | 0.313 | -0.061 | 9.99E-04 | T | C | N | *C16orf82* |
